# Correction: Import options for chemical energy carriers from renewable sources to Germany

**DOI:** 10.1371/journal.pone.0314578

**Published:** 2025-01-24

**Authors:** Johannes Hampp, Michael Düren, Tom Brown

S3 Table is incorrect. There are adjustments in the technology assumption used. Please see the correct S3 Table here.

## Supporting information

S3 Table S9 Table Technology assumptions. (PDF)

## References

[1] Hampp J, Düren M, Brown T (2023) Import options for chemical energy carriers from renewable sources to Germany. PLoS ONE 18(2): e0262340. doi: 10.1371/journal.pone.0281380 36757915 PMC9910710

