# Supplementary material for: Import options for chemical energy carriers from renewable sources to Germany
Source: PLoS One. 2023 Feb 9;18(2):e0262340. doi: 10.1371/journal.pone.0281380 (PMC9910710; doi:10.1371/journal.pone.0281380)
Supplement: S1 Appendix — (PDF) [file pone.0281380.s001.pdf]

S 1 Appendix ESF, LCoE and curtailment

Additional characteristics of the ESCs can become apparent by looking into the energy required for each ESC for synthesis, conversion and transport. We use the energy surplus factor (ESF) (Input electricity required per unit of energy delivered) instead of the energy efficiency (Share of energy delivered per unit of electricity generated) for this purpose. In Fig 12 the ESFs are shown on the left versus the LCoE for each ESC and exporter.

The ESFs for imports range from 1.4 (H_2 (g) pipeline and HVDC with subsequent electrolysis) to 5.4 (CH_4 (g) pipeline to H_2 from AU). The simpler and lower cost ESCs using HVDC and hydrogen pipelines have as a rule of thumb lower ESFs. The majority of shipping options are between 100 EUR/MWh_{th} to 200 EUR/MWh_{th} at ESFs ranging from 1.8 to 3.8. FT fuel ship-based imports are clustered around an ESF of 3.2. Notable outliers are the domestic production of methanol and FT fuel in DE which we will discuss further below. Comparing the ESFs can provide insight into the necessary of RES capacities and thus e.g. land requirements involved between different ESCs.

The right side of Fig 12 shows the ESFs versus the share of electricity curtailed. Curtailment in islanded system as in our scenarios may only be avoided with investments into storage capacities or increasing the flexibility of involved synthesis processes (CH_4, NH_3, methanol and FT fuel). We find a wide range of curtailment levels with the majority being below 20 % and no apparent correlation with ESCs and exporter.

Looking at the curtailment we again find the ESCs for methanol, methanol to H_2 and FT fuel with domestic production in DE as outliers. Closer inspection of these ESCs links the high curtailment rate to the available RES potentials for DE where, after domestic electricity demand was given priority in the supply curve, only onshore and offshore wind remain as RES and no PV is available to the ESCs. The high temporal correlation of the wind quality classes cause in combination with the inflexible synthesis processes for these chemical energy carriers the need to deploy high volumes of feedstock storage (H_2 and CO_2) as well as electrolysis capacities with low CF to be build. This in turn causes the investment costs and therefore LCoE to increase dramatically and significant curtailment on days of high wind speeds. This issue does not appear with any other exporter, as all other exporters have some PV potentials available, see S 5 Figs.

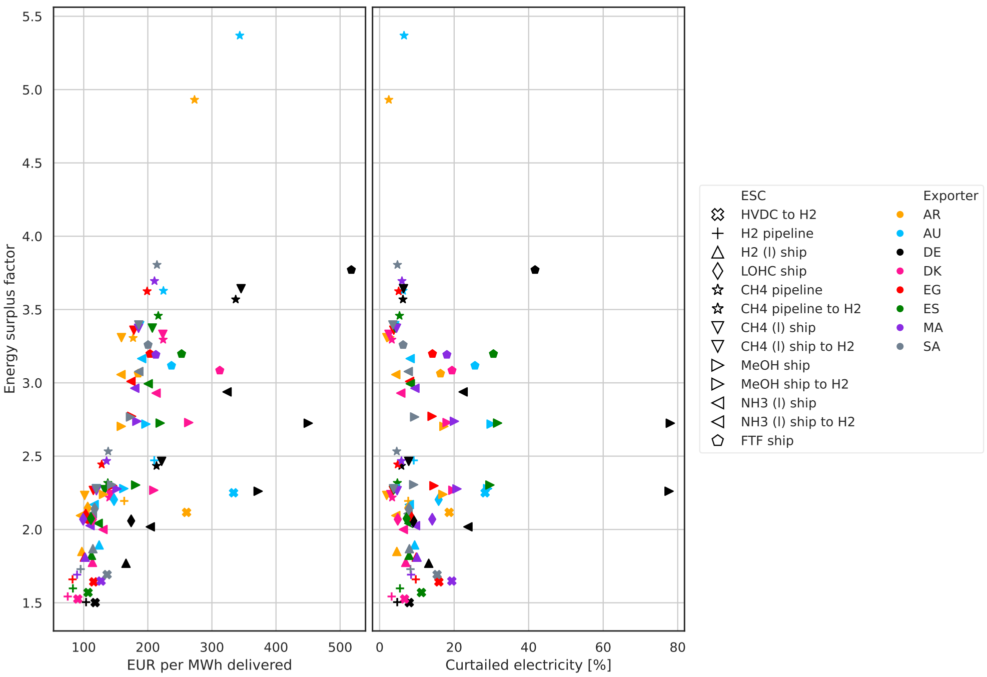

Fig 12. ESFs for ESCs and exporters displayed against LCoE (left) and electricity curtailment (right). Values shown are for 2030 and 10 % p.a. WACC scenarios. Simplicity (lower ESF) and costs of ESCs increase to some extent together while curtailment varies greatly between exporter and ESC driven by the available RES, inflexibility of synthesis processes and storage deployment.

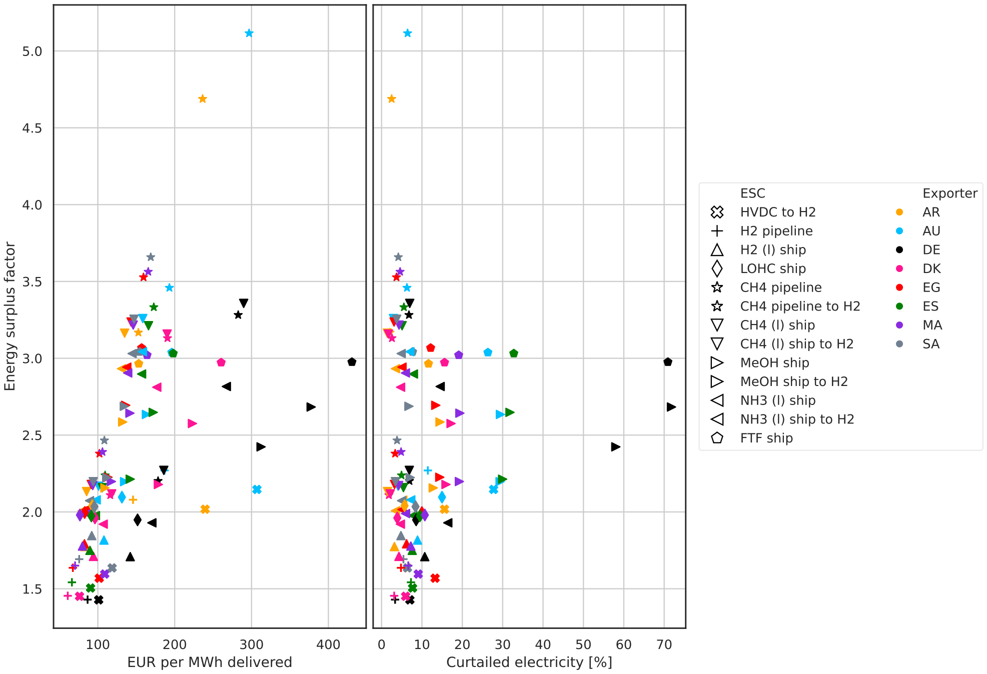

Fig 13. ESFs vs. LCoE and electricity curtailment for 2040 and 10 % p.a. WACC scenarios.

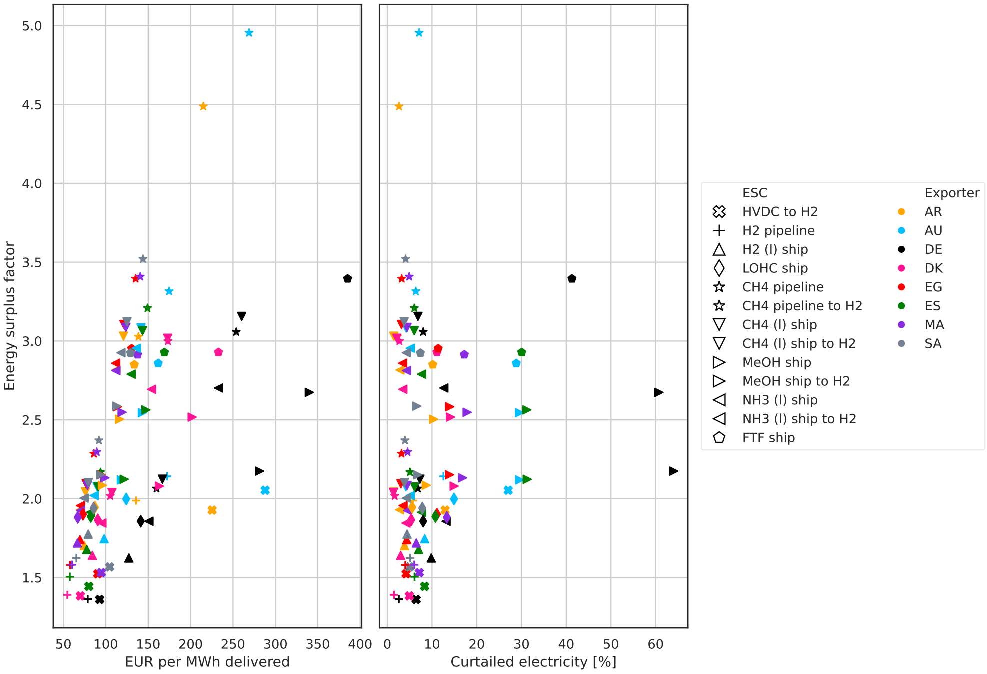

Fig 14. ESFs vs. LCoE and electricity curtailment for 2050 and 10 % p.a. WACC scenarios.

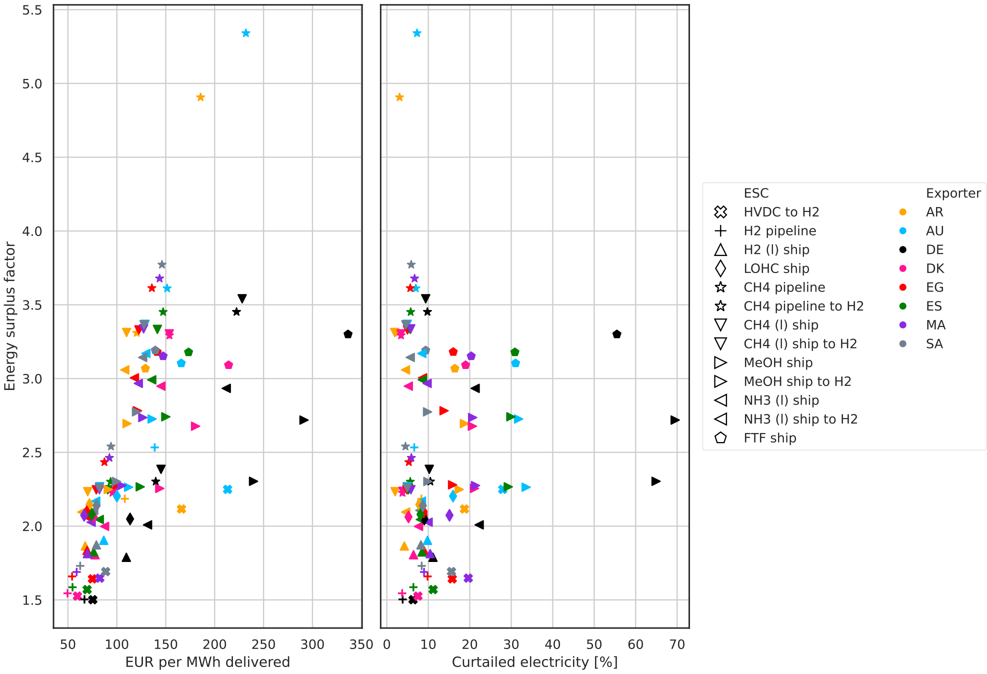

Fig 15. ESFs vs. LCoE and electricity curtailment for 2030 and 5 % p.a. WACC scenarios.

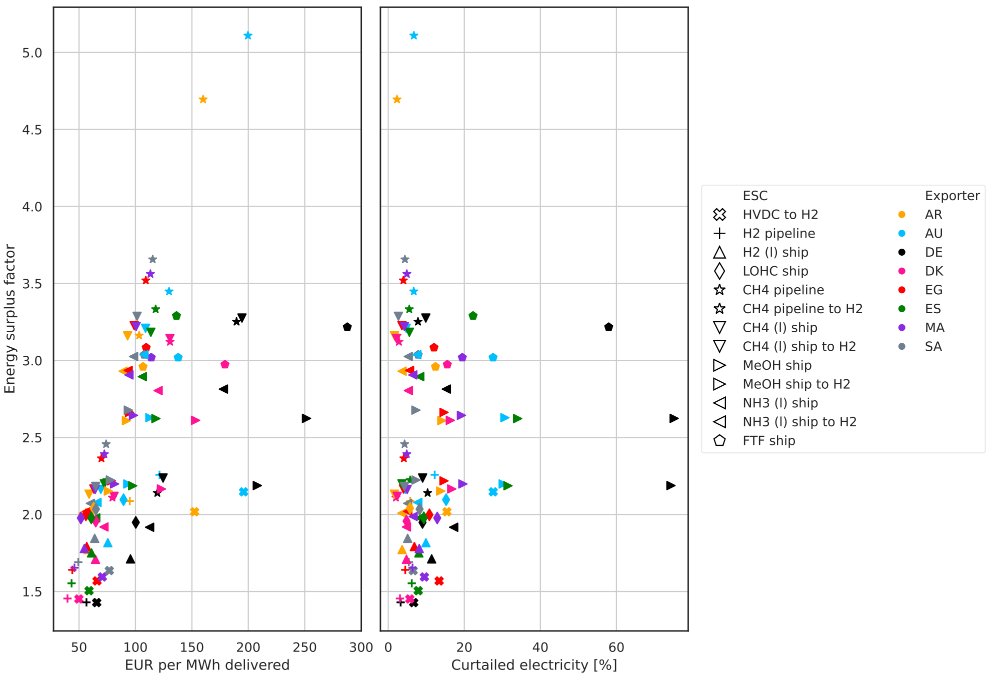

Fig 16. ESFs vs. LCoE and electricity curtailment for 2040 and 5 % p.a. WACC scenarios.

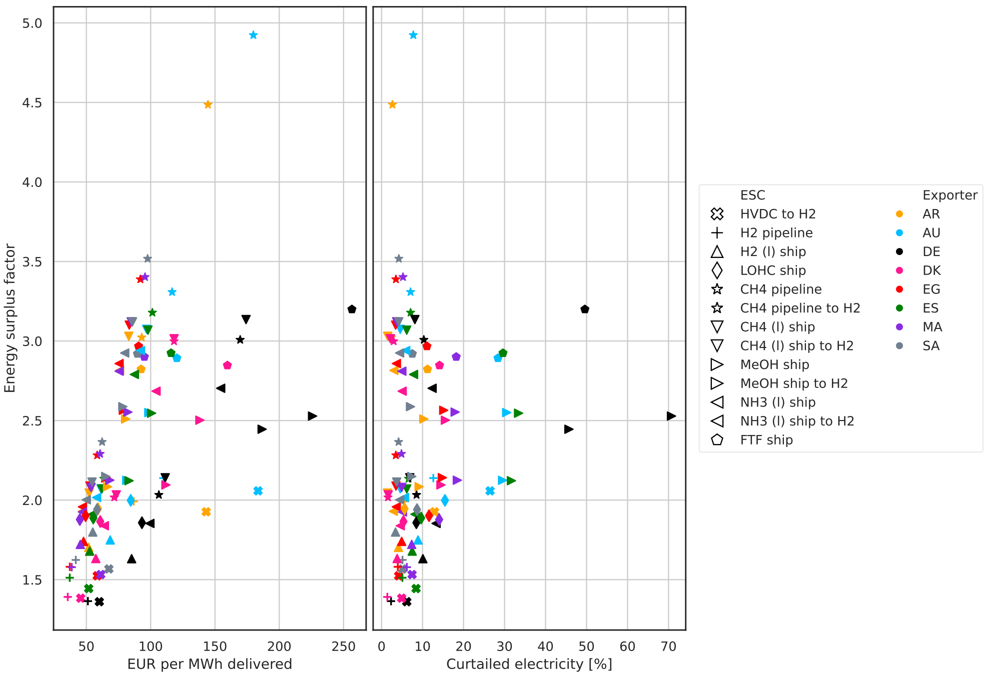

Fig 17. ESFs vs. LCoE and electricity curtailment for 2050 and 5 % p.a. WACC scenarios.
