# Supplementary material for: Import options for chemical energy carriers from renewable sources to Germany
Source: PLoS One. 2023 Feb 9;18(2):e0262340. doi: 10.1371/journal.pone.0281380 (PMC9910710; doi:10.1371/journal.pone.0281380)
Supplement: S2 Appendix — (PDF) [file pone.0281380.s002.pdf]

S 2 Appendix Technical model structure

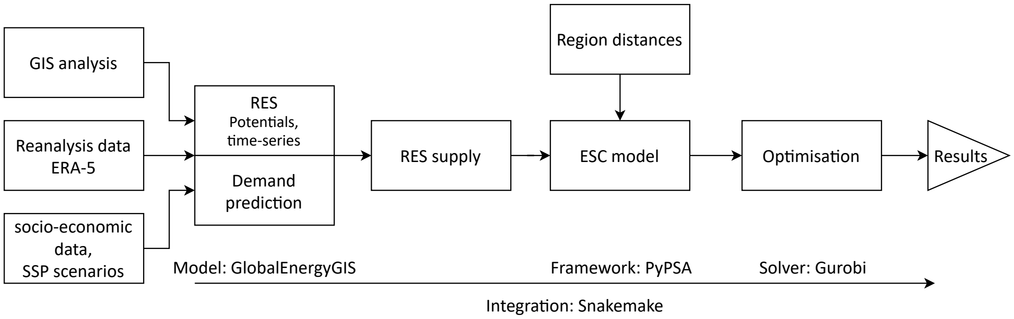

Fig 18. Model structure, underlying workflow and software used for this study.
