# Supplementary material for: Import options for chemical energy carriers from renewable sources to Germany
Source: PLoS One. 2023 Feb 9;18(2):e0262340. doi: 10.1371/journal.pone.0281380 (PMC9910710; doi:10.1371/journal.pone.0281380)
Supplement: S3 Appendix — (PDF) [file pone.0281380.s003.pdf]

S 3 Appendix Model equations

The underlying model equations for each scenario are constructed through the open source framework PyPSA. PyPSA is a framework for building power and energy system models for running capacity expansion optimisation among other features. A detailed description of the mathematical background can be found in [1] and in the online documentation at pypsa.readthedocs.io.

PyPSA models are built using elemental components, such as generators, links, loads, stores and buses. Buses are the fundamental nodes to which all other components attach. In this study buses represent intermediary steps in the conversion between materials and energy carriers and different locations during the transport of materials or energy. Each bus is associated with one specific type of energy or material. Energy and materials enter a model through generators or links with efficiencies $\eta > 1$. Links connect different buses and represent in this study conversion steps or the transport of materials or energy. Each link's inflows and outflows are linked to individual effficiencies. Loads represents sinks for energy and materials where they are consumed, i.e. they leave a model.

From the interconnected components PyPSA generates a network (graph) model for which then energy and material flows are conserved [1], i.e. for at each bus n for each timestep t

$$\sum_r g_{n,r,t} + \sum_s h_{n,s,t} + \sum_l \alpha_{l,n,t} f_{l,t} = d_{n,t} \qquad \forall i, t \tag{9}$$

where $g_{n,r,t}$ is the inflow from generator r at bus n at timestep t, $h_{n,s,t}$ is the flow from or into store s attached to bus n at timestep t, $f_{l,t}$ incoming or outgoing flows through link l, $\alpha_{l,n,t}$ the incidence matrix and $d_{i,t}$ the outflow through loads attached to the bus. The incidence matrix takes on the values

$$\alpha_{l,n,t} = \begin{cases} -1 & \text{if } l \text{ starts at } n \\ \eta_{l,n,t} & \text{if } l \text{ ends at } n \end{cases} \tag{10}$$

with $\eta_{l,n,t}$ being the specific efficiency for the link t ending in n.

The pipeline efficiency η^{pipe} is determined from the pipeline efficiency per 1000 km η_0^{pipe} (losses and energy consumption for pressure boosting compressors) and the pipeline length d in [km]

$$\eta^{\text{pipe}} = 1 - \eta_0^{\text{pipe}\,\frac{d^{\text{pipe}}}{1000}} \tag{11}$$

The efficiencies for HVDC connections are calculated analogously.

The shipping efficiency η^{ship} is determined by the boil-off and the propulsion energy demand as ships are assumed to use their cargoed energy carrier also as propulsion fuel. The lower efficiency, i.e. higher losses due to propulsion or boil off, are used as total efficiency. The total propulsion energy demand for outbound and return journey is determined by the shipping distance d^{ship} in [km], the ship's specific energy demand e in [MW h/km] and the ship's total cargo capacity c in [MW h].

Boil-off is only considered for the outbound journey as we assume the absolute boil-off during the return journey with near empty cargo hold to be minimal. The boil-off for the outbound journey is determined by the specific boil-off rate b^{ship} in [%/h] and the adjusted outbound journey travel time $t^{\text{ship}}_{\text{outbound, adjusted}}$ in [h].

The total shipping efficiency is thus calculated as

$$\eta^{\text{ship}} = \min\left\{1 - 2d^{\text{ship}} \cdot \frac{e}{c}, \left(1 - b^{\text{ship}}\right)^{t^{\text{ship}}_{\text{outbound, adjusted}}}\right\} \tag{12}$$

The outbound journey travel time $t^{\text{ship}}_{\text{outbound}}$ is determined by the ship's average speed v^{ship} and shipping distance d^{ship}

$$t^{\text{ship}}_{\text{outbound}} = \frac{d^{\text{ship}}}{v^{\text{ship}}} \tag{13}$$

The round-trip time for shipping is further affected by the time required for loading and unloading the ship $t^{\text{ship}}_{\text{(un-)loading}}$

$$t^{\text{ship}}_{\text{round-trip}} = 2t^{textship}_{\text{outbound}} + 2t^{\text{ship}}_{\text{(un-)loading}} \tag{14}$$

The adjusted outbound journey time $t^{\text{ship}}_{\text{outbound, adjusted}}$ is then determined by stretching the round-trip journey time. Stretching the journey time keeps the number of trips per year the same while reducing the number of hours a ship is not engaged in transporting cargo to a minimum.

$$t^{\text{ship}}_{\text{outbound, adjusted}} = t^{\text{ship}}_{\text{outbound}} + \text{round}\left(\frac{1}{2}\left\lceil \frac{t^{\text{ship}}_{\text{gap}}}{n^{\text{ship}}_{\text{journeys}}} \right\rceil\right) \tag{15}$$

Here round$(\dots)$ is rounding to the next integer,

$$t^{\text{ship}}_{\text{gap}} = 8760\,\text{h} \cdot \text{mod}\, t^{\text{ship}}_{\text{round-trip}} \tag{16}$$

the time a ship would be idle per year (8760 h) if the travel time was not adjusted and

$$n^{\text{ship}}_{\text{journeys}} = \left\lceil \frac{8760\,\text{h}}{t^{\text{ship}}_{\text{round-trip}}} \right\rceil \tag{17}$$

the number of journeys a ship can undertake per year. The shipping process also suffers from cargo losses during the loading and unloading process of the ship l in %. These losses are accounted for separately and are not part of the shipping efficiency. Technical shipping parameters are listed in S 11 Table.

For specific synthesis processes (methanation, Haber-Bosch synthesis, methanol synthesis, Fischer-Tropsch synthesis) a must-run constraint is implemented in the model which forces a minimum capacity of synthesis capacitiy of each ESC to be online at all times. The constraint is

$$p_{\text{nom}}(t, \text{plant}) \geq p_{\text{nom, min}}(t, \text{plant}) \qquad \forall t \tag{18}$$

with p_{nom} being the normalised output of a synthesis in timestep t of a specific synthesis plant and $p_{\text{nom, min}}$ the normalised lower limit must-run availability, e.g. 0.9425.

References

1. Brown T, Hörsch J, Schlachtberger D. PyPSA: Python for Power System Analysis. Journal of Open Research Software. 2018;6(1). doi:10.5334/jors.188.
