# Supplementary material for: Import options for chemical energy carriers from renewable sources to Germany
Source: PLoS One. 2023 Feb 9;18(2):e0262340. doi: 10.1371/journal.pone.0281380 (PMC9910710; doi:10.1371/journal.pone.0281380)
Supplement: S1 Fig — (PDF) [file pone.0281380.s004.pdf]

S 4 Figs Energy Supply Chains visualisations

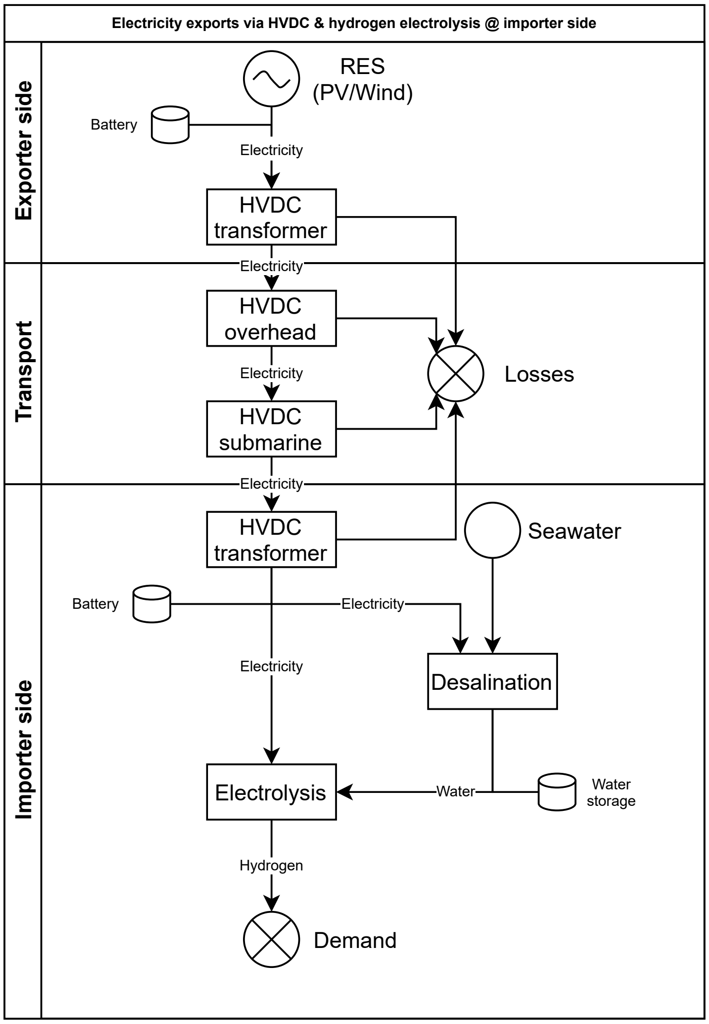

Fig 19. ESC schematic for HVDC electricity imports and domestic hydrogen electrolysis.

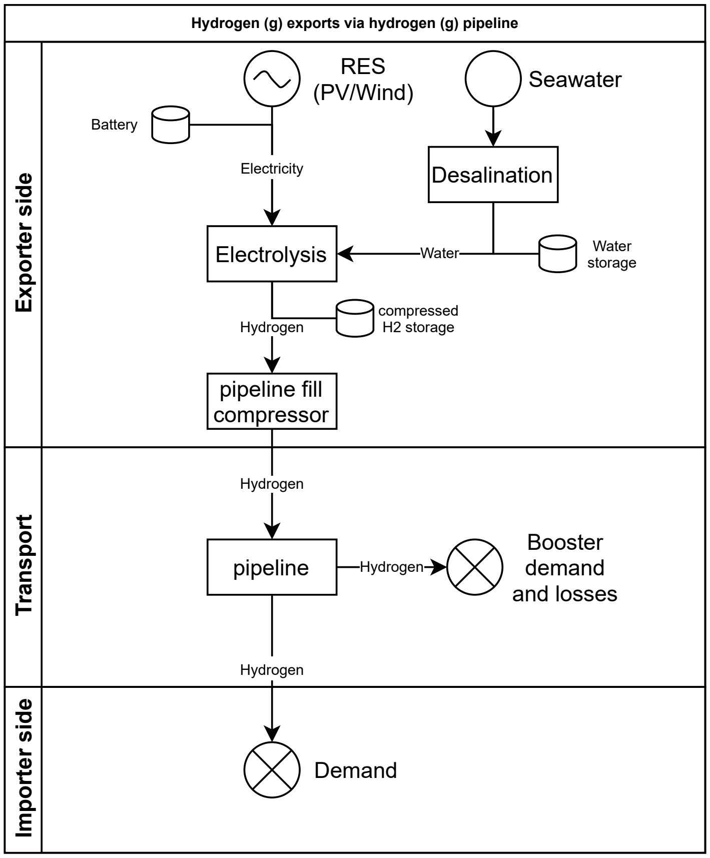

Fig 20. ESC schematic for imports of hydrogen gas by pipeline.

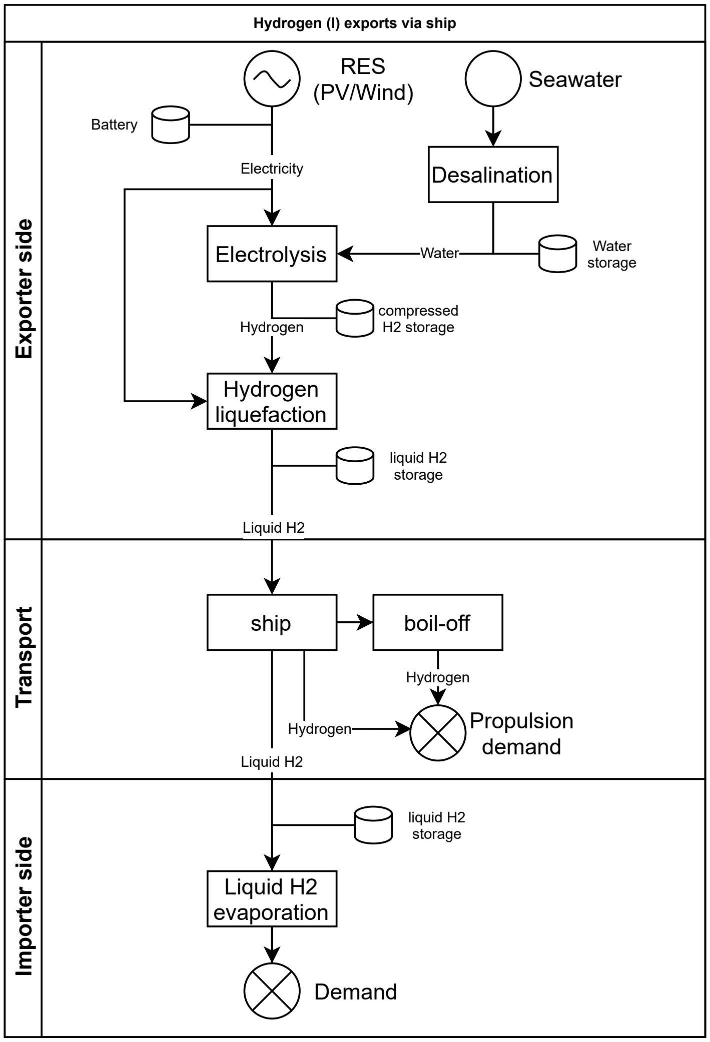

Fig 21. ESC schematic for liquid hydrogen imports by ship.

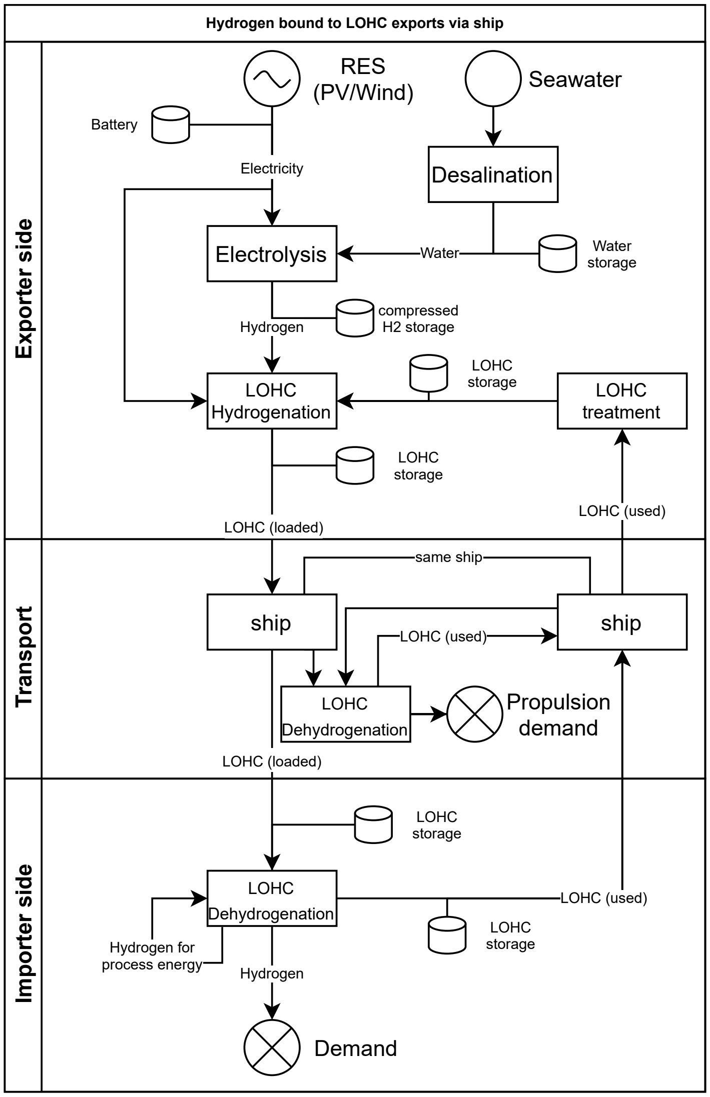

Fig 22. ESC schematic for hydrogen imports using LOHC by ship.

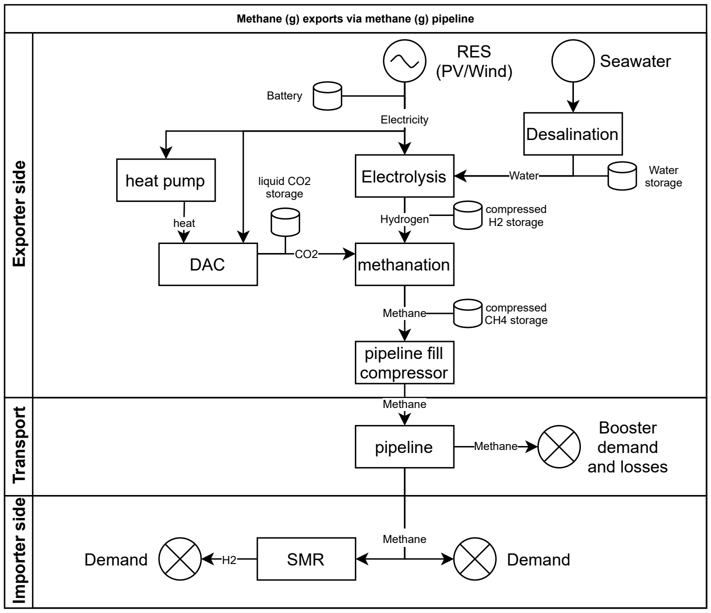

Fig 23. ESC schematic for imports of methane gas by pipeline. To serve an optional demand of hydrogen, methane may be split via steam methane reforming.

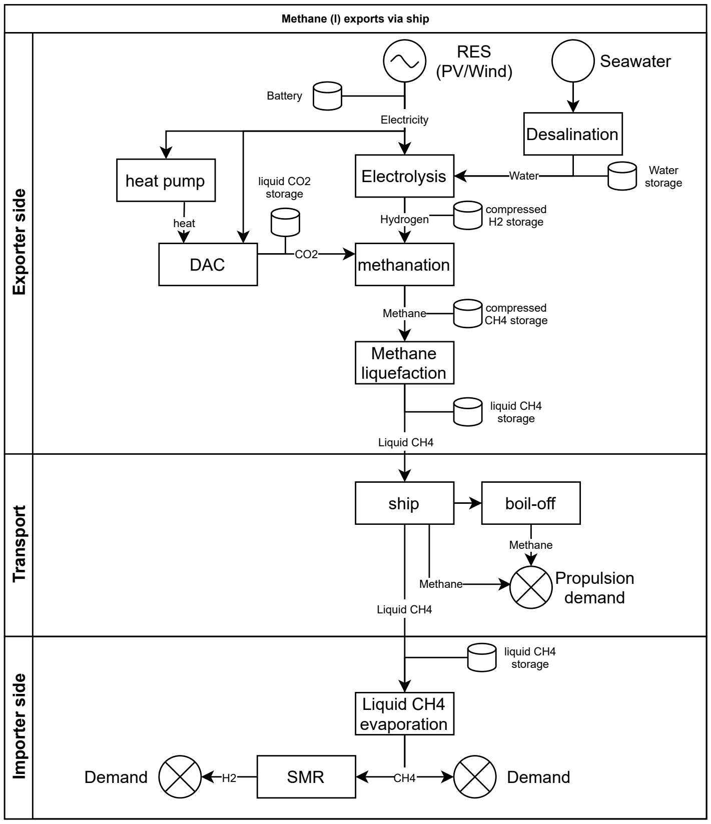

Fig 24. ESC schematic for liquid methane imports by ship. To serve an optional demand of hydrogen, methane may be split via steam methane reforming.

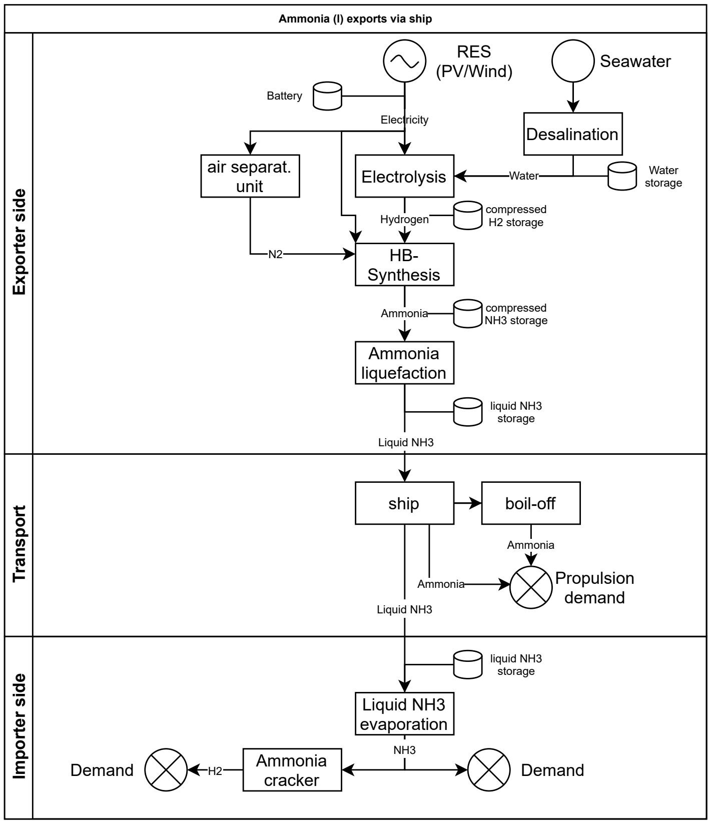

Fig 25. ESC schematic for liquid ammonia imports by ship. The ASU is assumed to provide nitrogen on demand without a dedicated nitrogen gas feedstock storage following [1]. To serve an optional demand of hydrogen, ammonia may be cracked in an ammonia cracker into hydrogen and nitrogen.

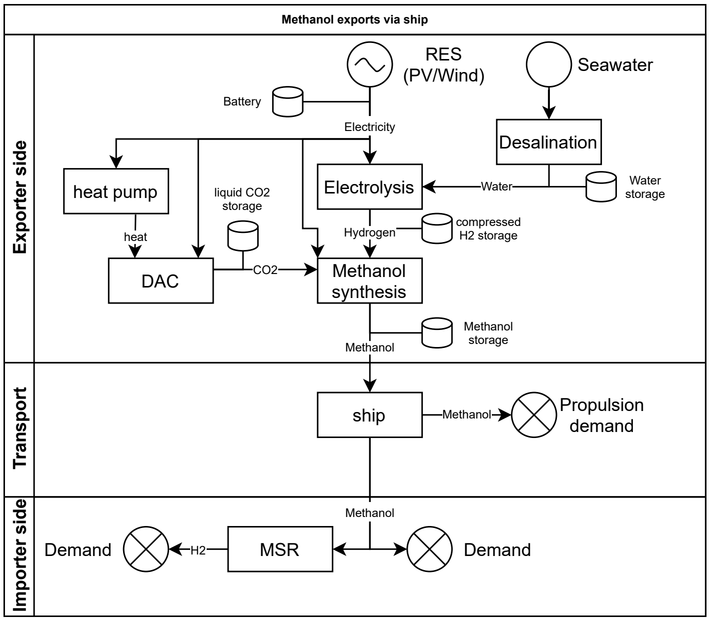

Fig 26. ESC schematic for methanol imports by ship. To serve an optional demand of hydrogen, methanol may be split via methanol steam reforming.

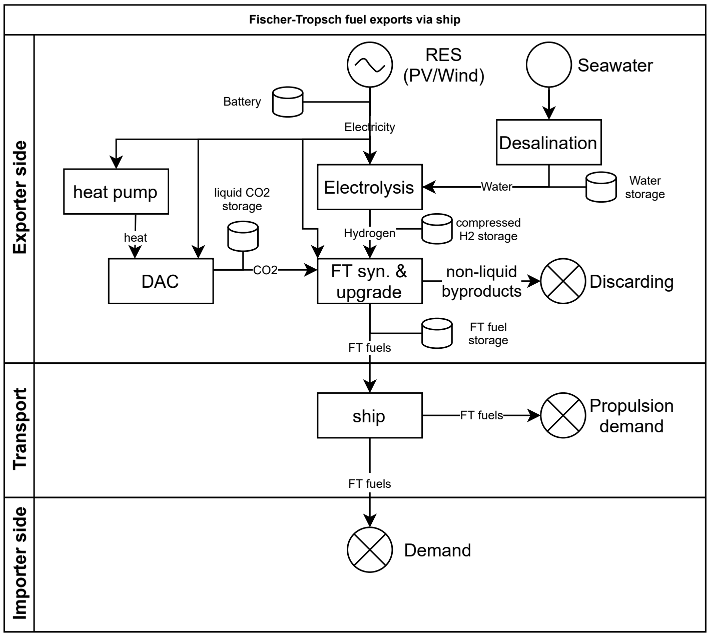

Fig 27. ESC schematic for FT fuel imports by ship.

References

1. Bañares-Alcántara R, Iii GD, Fiaschetti M, Grünewald P, Lopez JM, Tsang E, et al. Analysis of Islanded Ammonia-based Energy Storage Systems; 2015. Available from: http://www2.eng.ox.ac.uk/systemseng/publications/Ammonia-based_ESS.pdf.
