# Supplementary material for: Import options for chemical energy carriers from renewable sources to Germany
Source: PLoS One. 2023 Feb 9;18(2):e0262340. doi: 10.1371/journal.pone.0281380 (PMC9910710; doi:10.1371/journal.pone.0281380)
Supplement: S2 Fig — (PDF) [file pone.0281380.s005.pdf]

S 5 Figs Electricity generation mix and supply curves

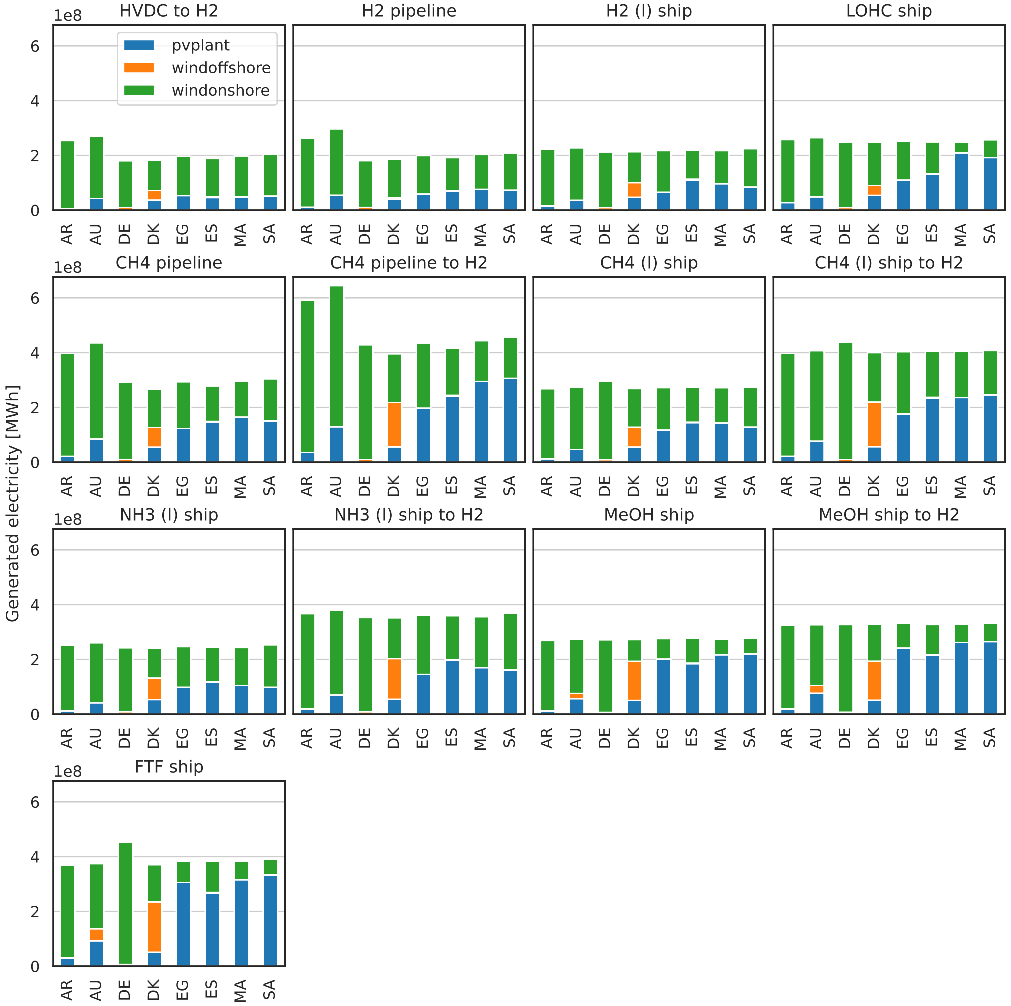

Fig 28. Electricity generation from RES ESC and exporting country under 10 % p.a. WACC scenario for 2030.

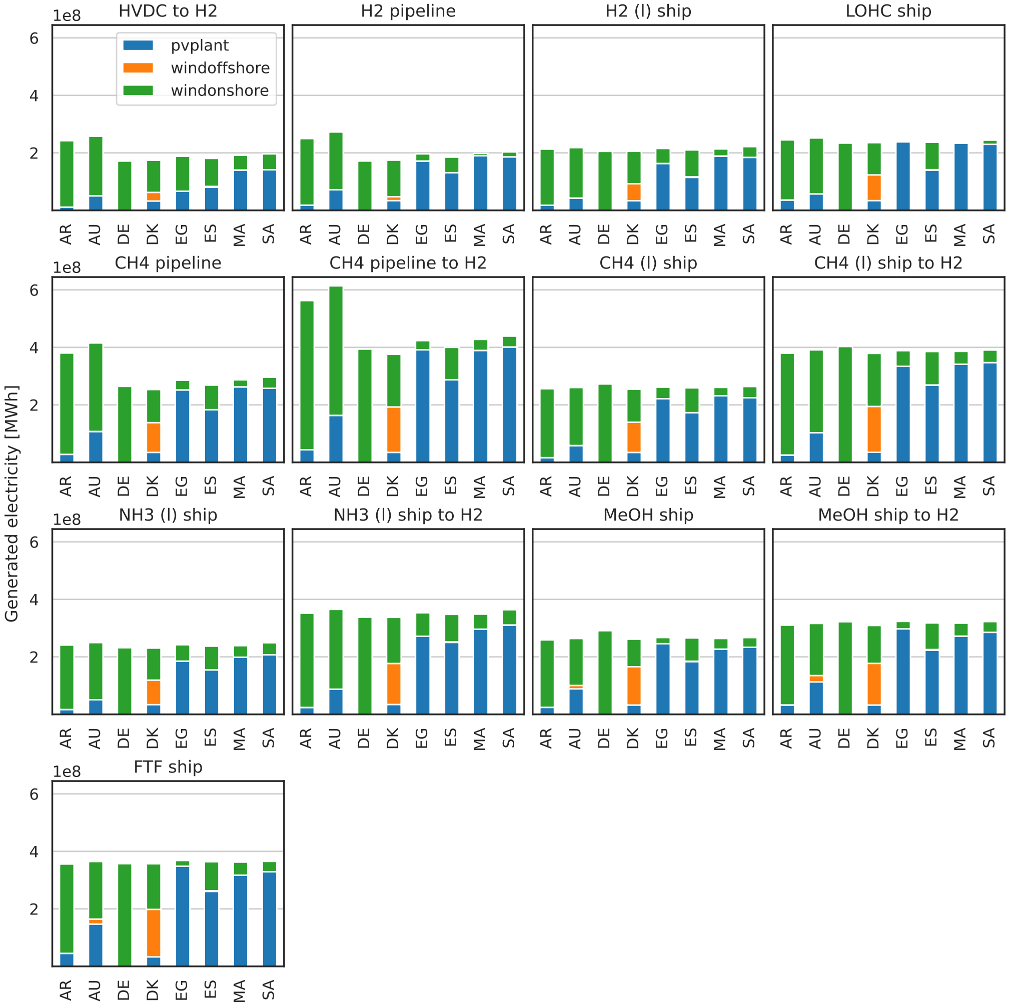

Fig 29. Electricity generation from RES ESC and exporting country under 10 % p.a. WACC scenario for 2040.

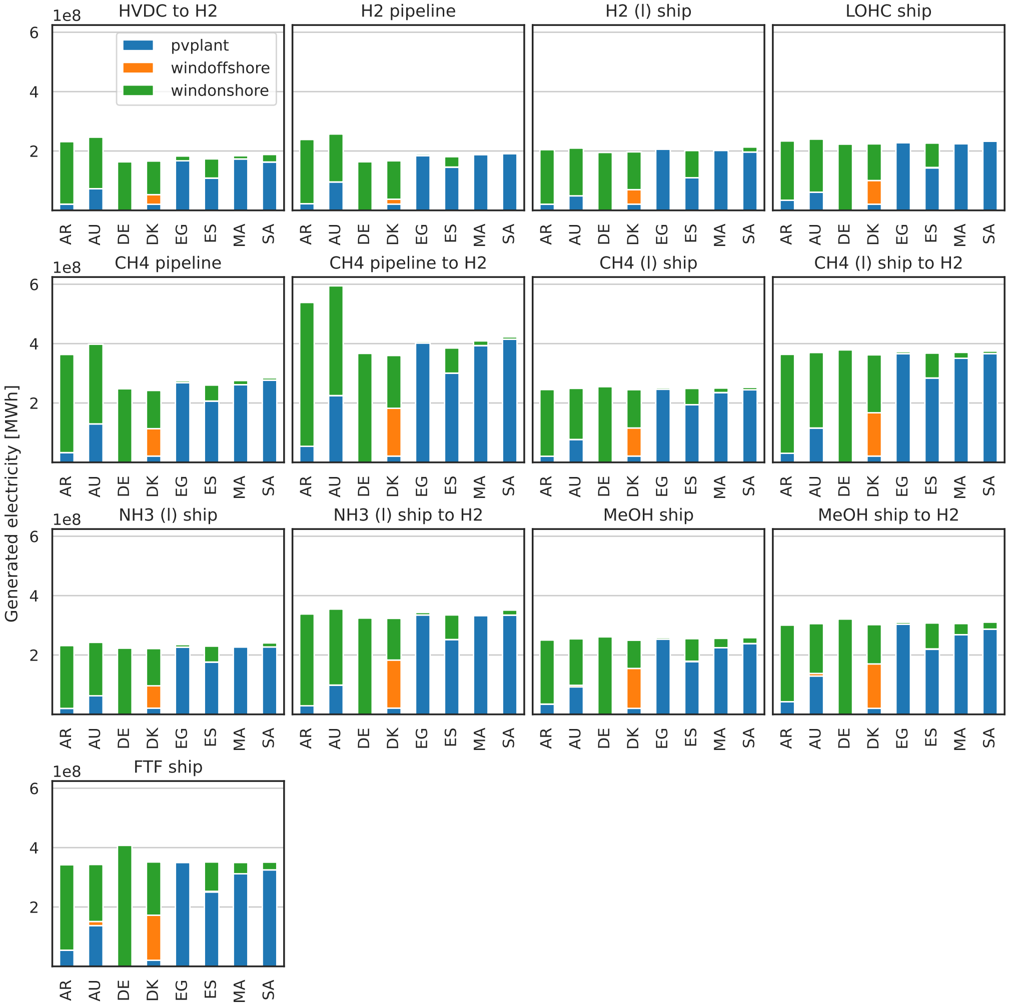

Fig 30. Electricity generation from RES ESC and exporting country under 10 % p.a. WACC scenario for 2050.

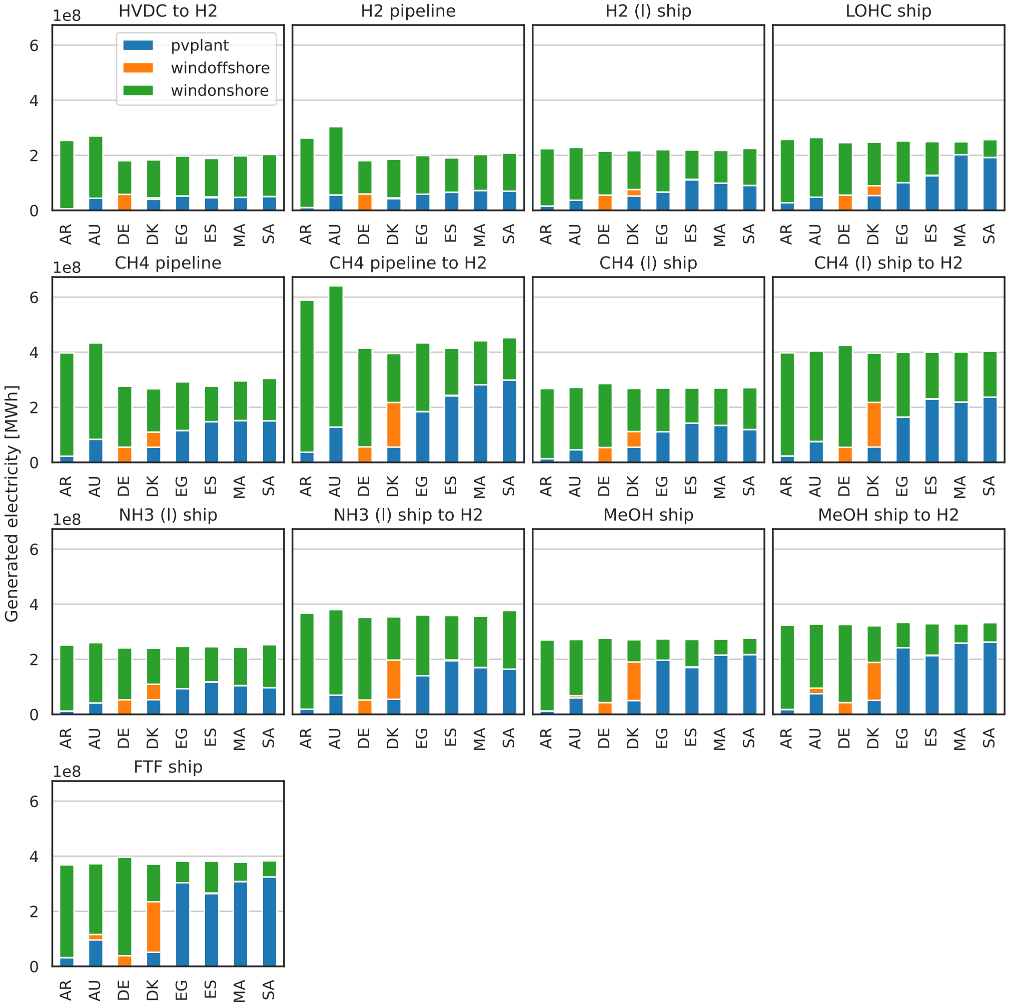

Fig 31. Electricity generation from RES ESC and exporting country under 5 % p.a. WACC scenario for 2030.

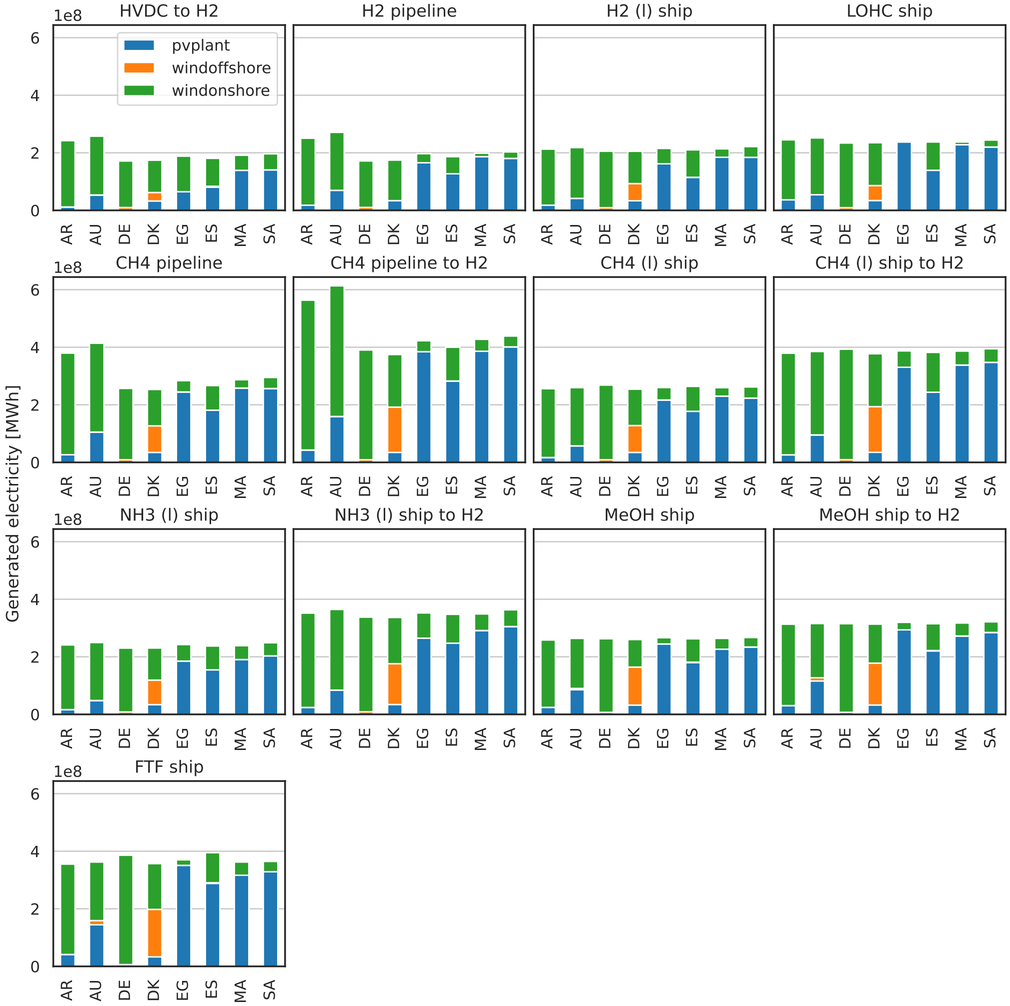

Fig 32. Electricity generation from RES ESC and exporting country under 5 % p.a. WACC scenario for 2040.

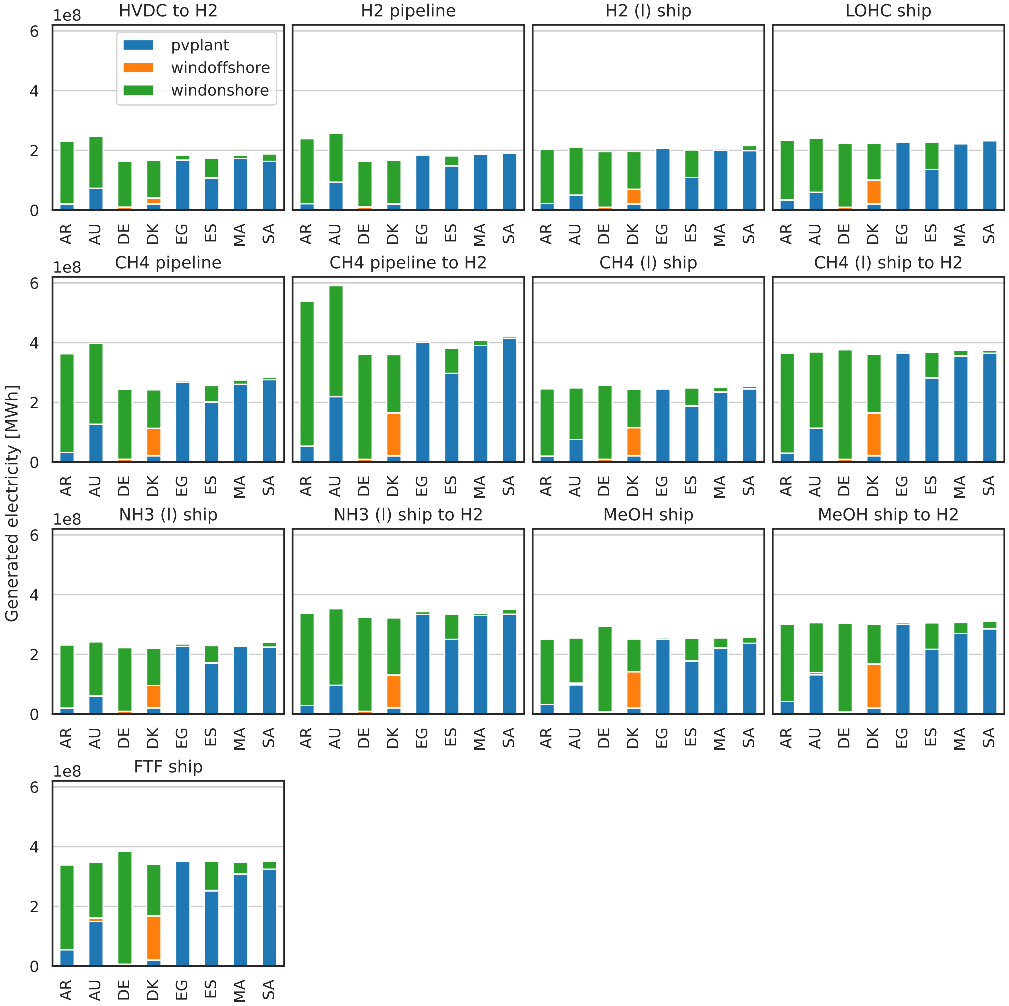

Fig 33. Electricity generation from RES ESC and exporting country under 5 % p.a. WACC scenario for 2050.

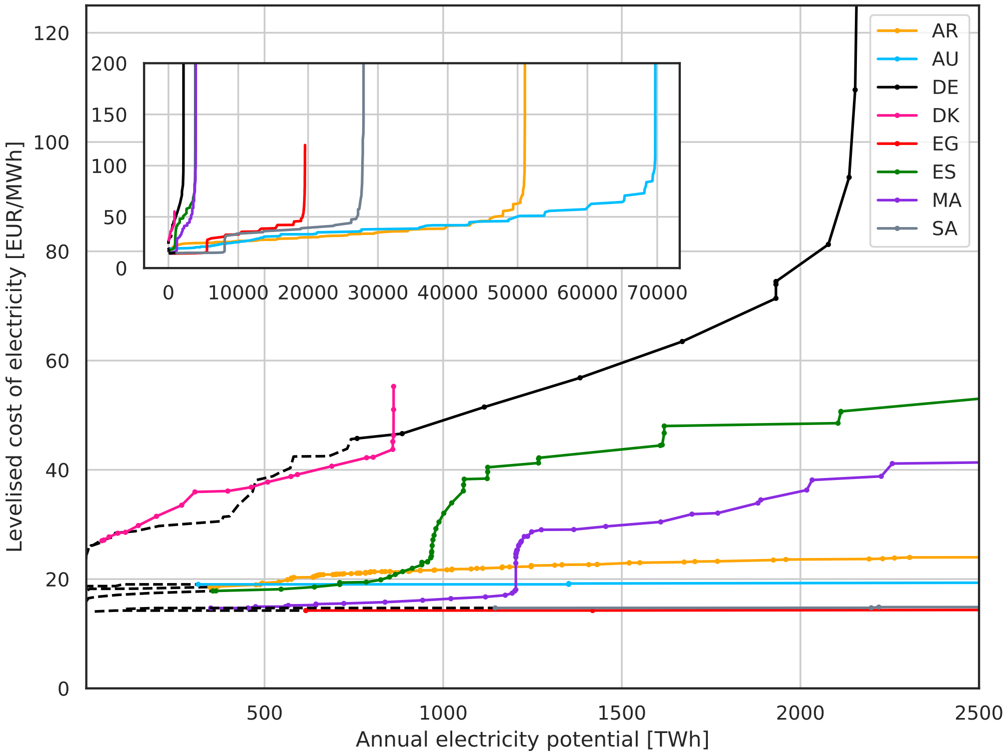

Fig 34. Similar figure as Fig 3 of electricity supply curves at 10 % p.a. WACC for year 2040.

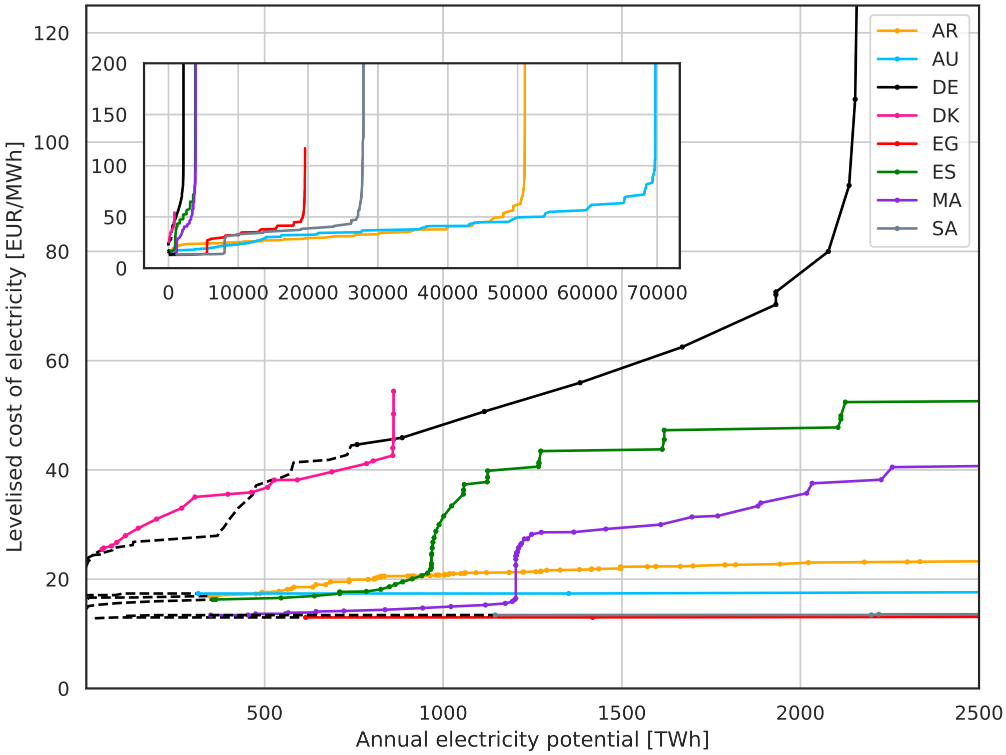

Fig 35. Similar figure as Fig 3 of electricity supply curves at 10 % p.a. WACC for year 2050.

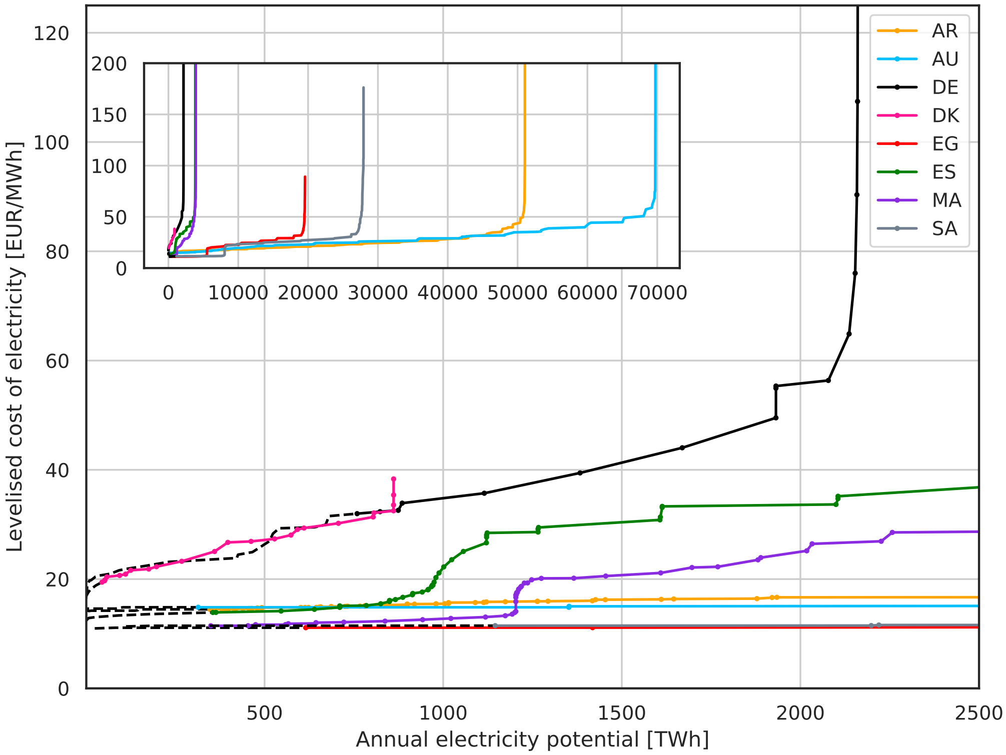

Fig 36. Similar figure as Fig 3 of electricity supply curves at 5 % p.a. WACC for year 2030.

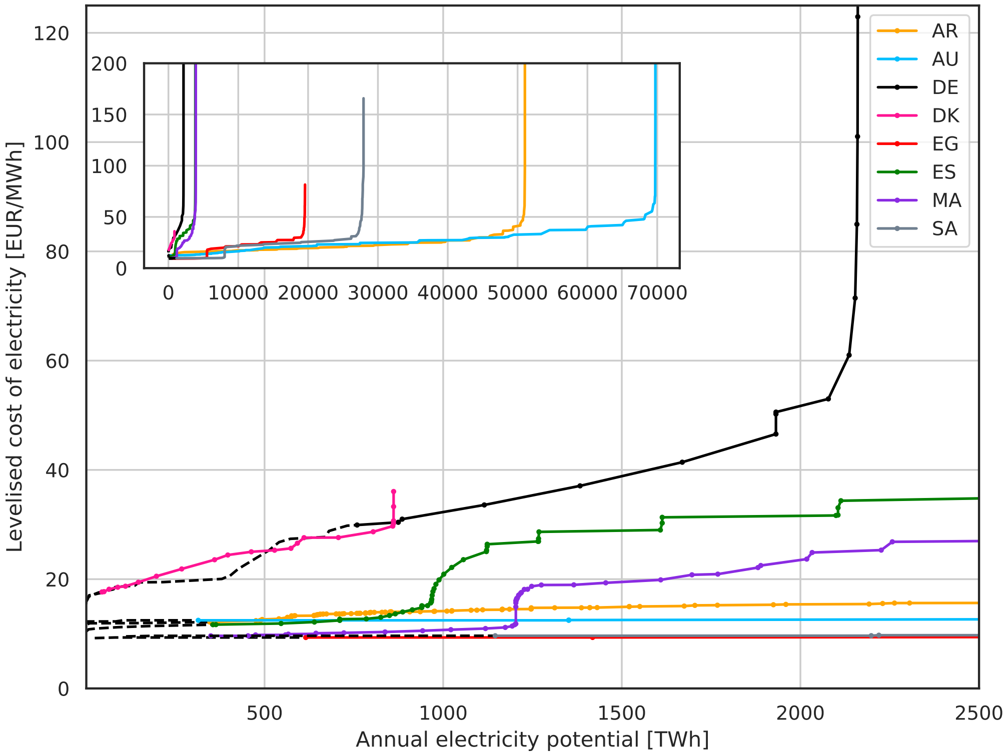

Fig 37. Similar figure as Fig 3 of electricity supply curves at 5 % p.a. WACC for year 2040.

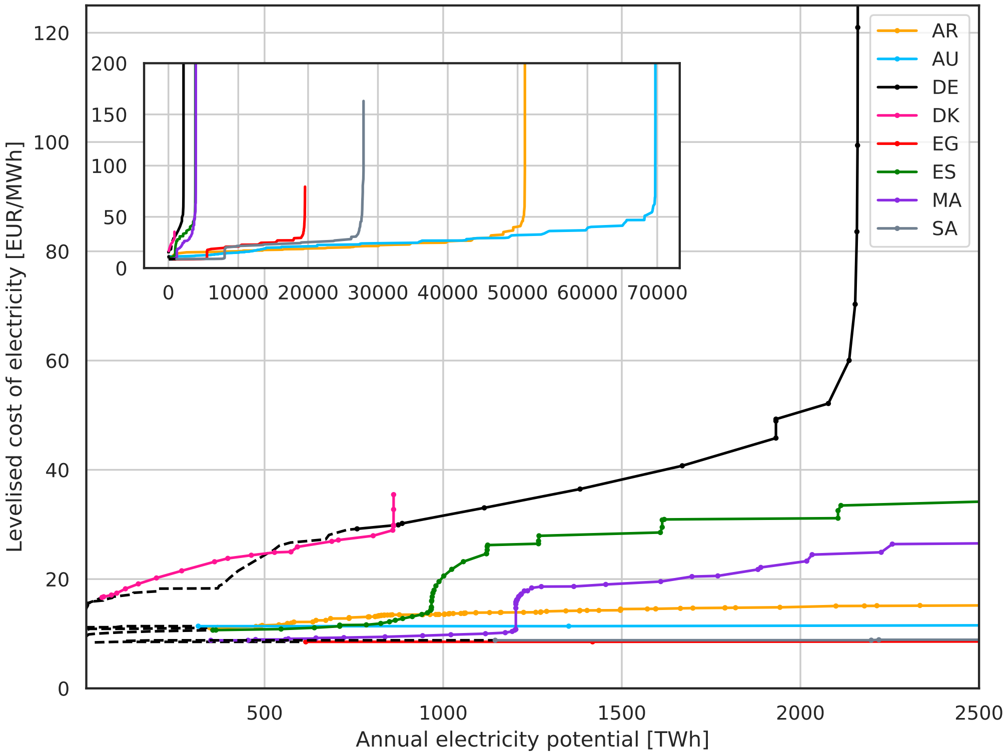

Fig 38. Similar figure as Fig 3 of electricity supply curves at 5 % p.a. WACC for year 2050.
