# Supplementary material for: Import options for chemical energy carriers from renewable sources to Germany
Source: PLoS One. 2023 Feb 9;18(2):e0262340. doi: 10.1371/journal.pone.0281380 (PMC9910710; doi:10.1371/journal.pone.0281380)
Supplement: S3 Fig — (PDF) [file pone.0281380.s006.pdf]

S 6 Figs Cost composition for other years, lower WACC

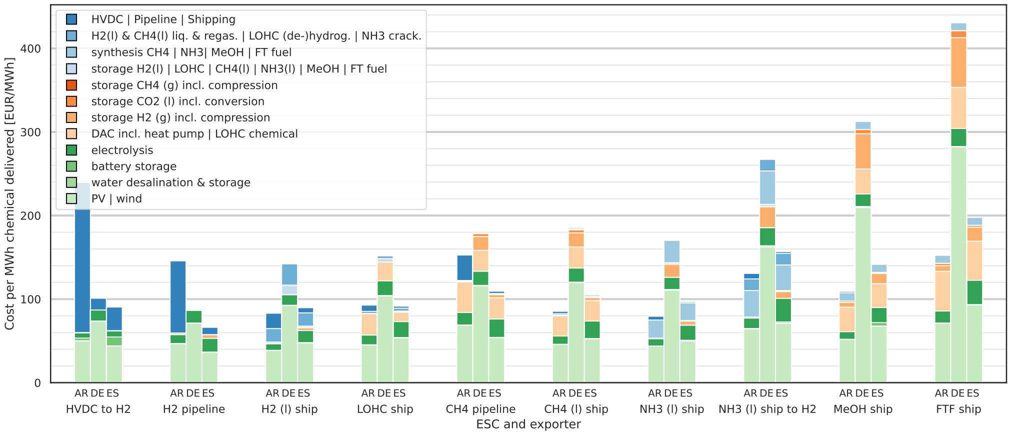

Fig 39. Similar figure as Fig 6 of cost compositions for selected ESCs at 10 % p.a. WACC for year 2040.

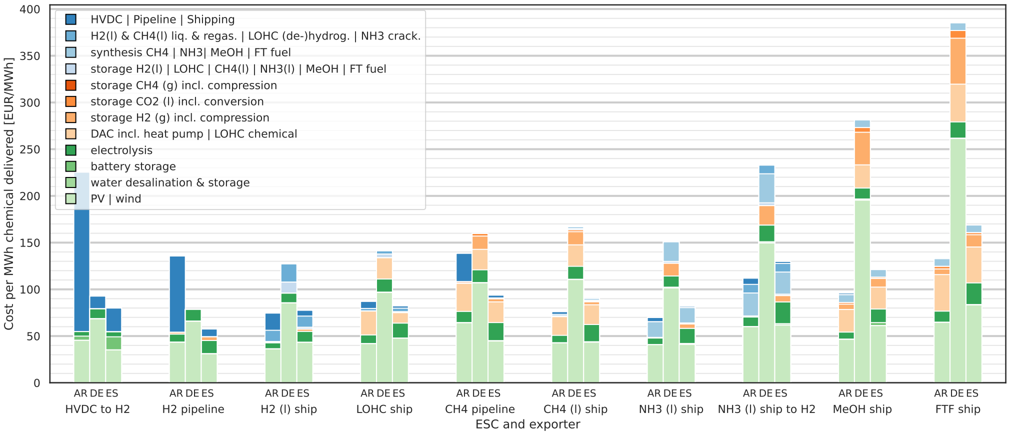

Fig 40. Similar figure as Fig 6 of cost compositions for selected ESCs at 10 % p.a. WACC for year 2050.

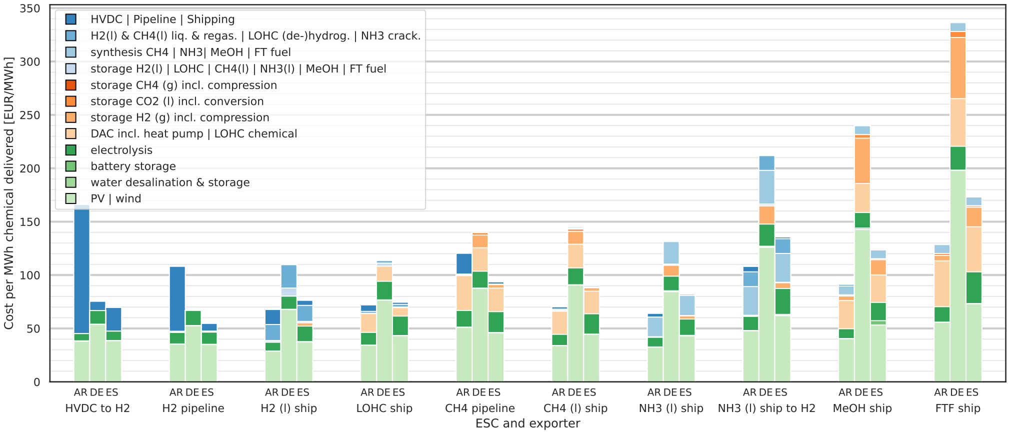

Fig 41. Cost compositions for selected ESCs at 5 % p.a. WACC for year 2030.

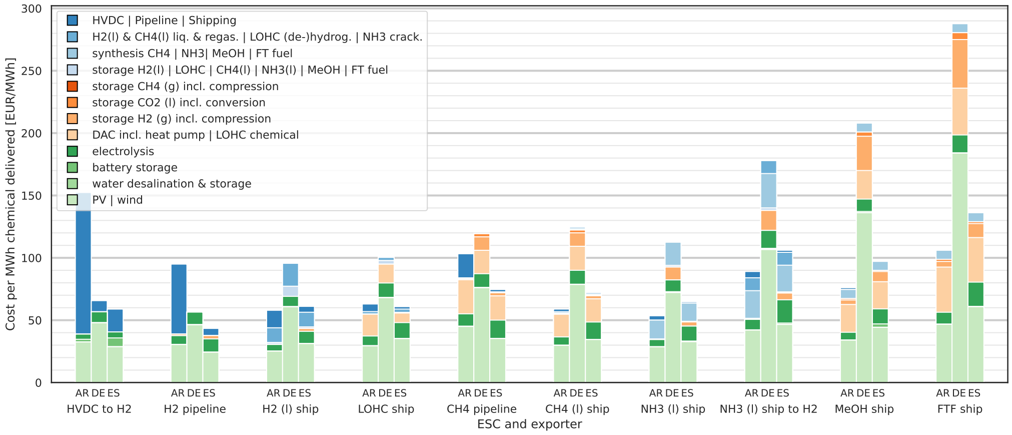

Fig 42. Cost compositions for selected ESCs at 5 % p.a. WACC for year 2040.

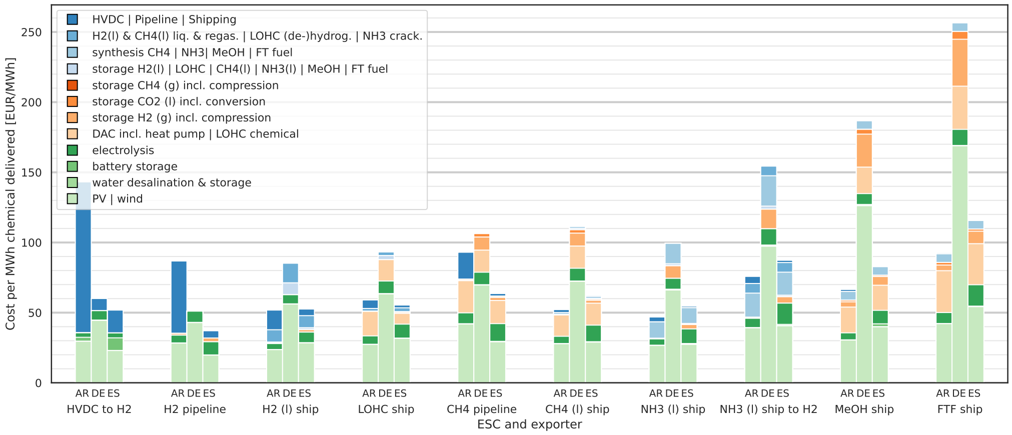

Fig 43. Cost compositions for selected ESCs at 5 % p.a. WACC for year 2050.
