# Supplementary material for: Import options for chemical energy carriers from renewable sources to Germany
Source: PLoS One. 2023 Feb 9;18(2):e0262340. doi: 10.1371/journal.pone.0281380 (PMC9910710; doi:10.1371/journal.pone.0281380)
Supplement: S4 Fig — (PDF) [file pone.0281380.s007.pdf]

S 12 Figs RES eligible area masks

The following figures show the eligible areas considered for onshore wind and PV RES for the countries while determining their RES potentials. For this study no differentiation between 'plant A' and 'plant B' was used. Both eligible types of area were combined to the total eligible area for each technology.

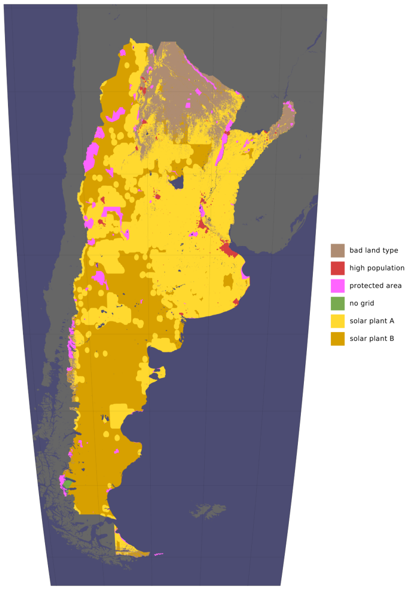

(a) PV mask for Argentina.

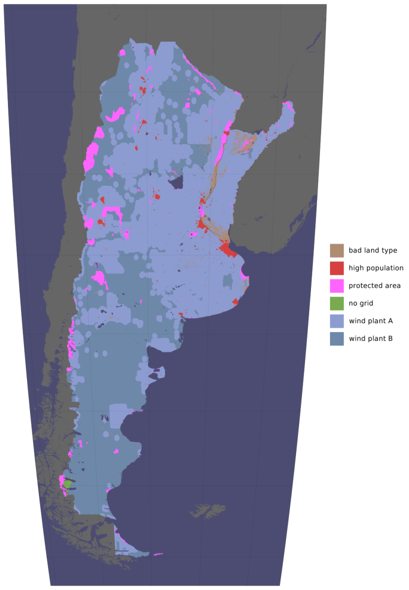

(b) Onshore wind mask for Argentina.

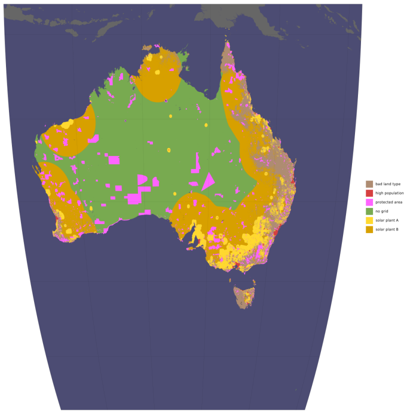

(c) PV mask for Australia.

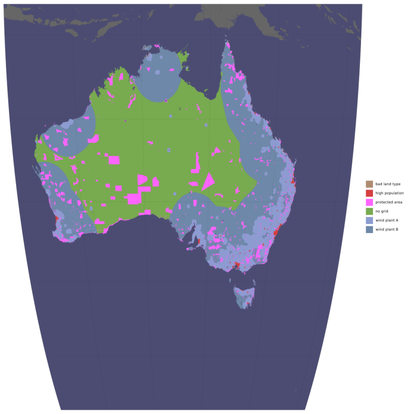

(d) Onshore wind mask for Australia.

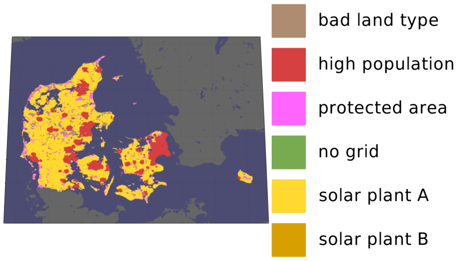

(e) PV mask for Denmark.

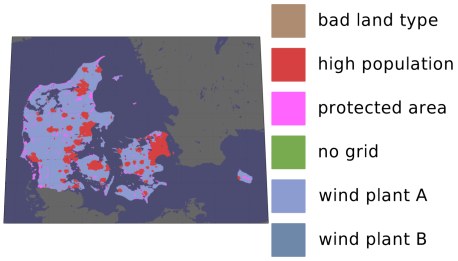

(f) Onshore wind mask for Denmark.

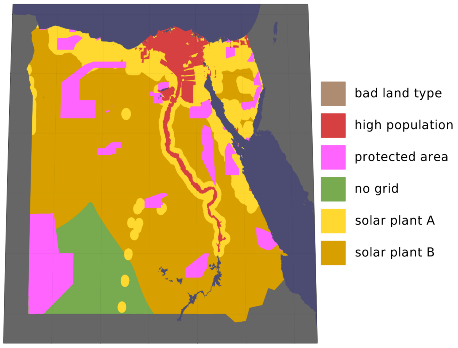

(g) PV mask for Egypt.

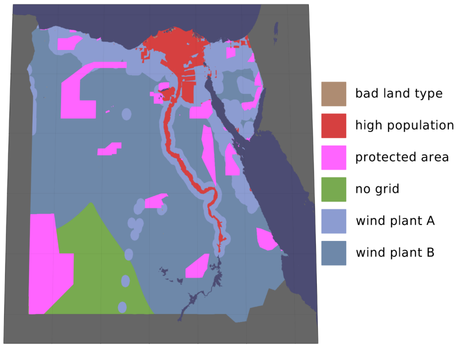

(h) Onshore wind mask for Egypt.

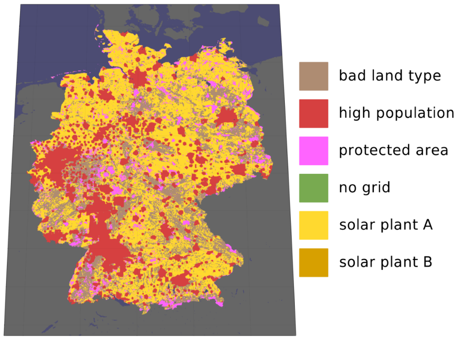

(i) PV mask for Germany.

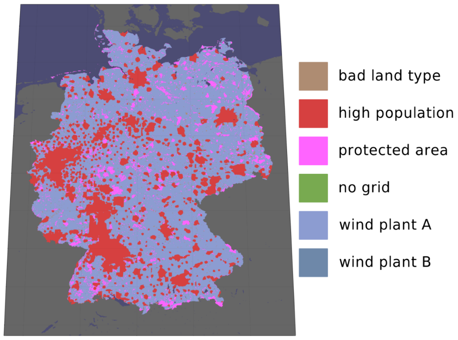

(j) Onshore wind mask for Germany.

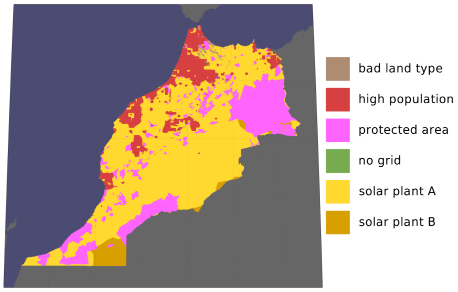

(k) PV mask for Morocco.

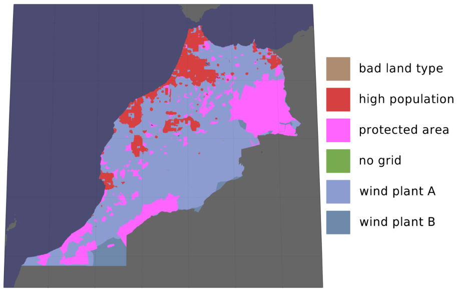

(l) Onshore wind mask for Morocco.

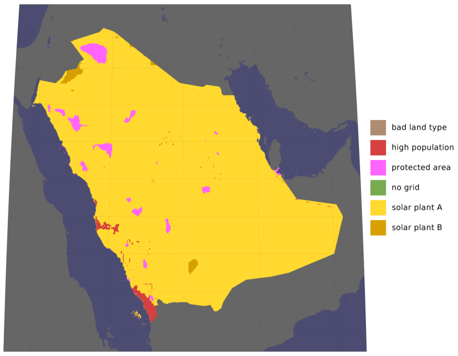

(m) PV mask for Saudi Arabia.

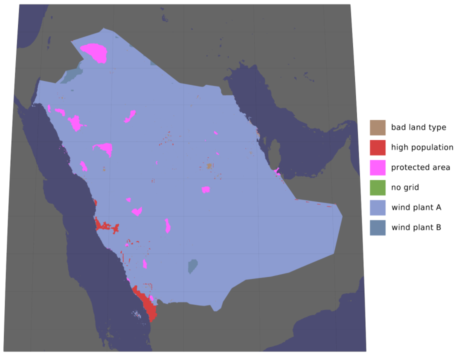

(n) Onshore wind mask for Saudi Arabia.

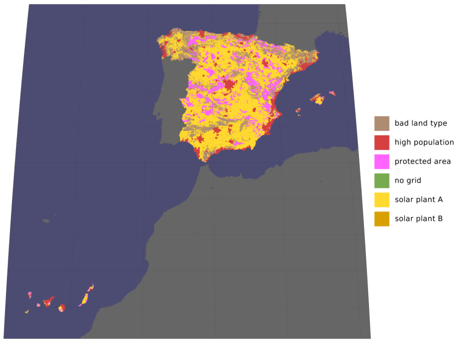

(o) PV mask for Spain.

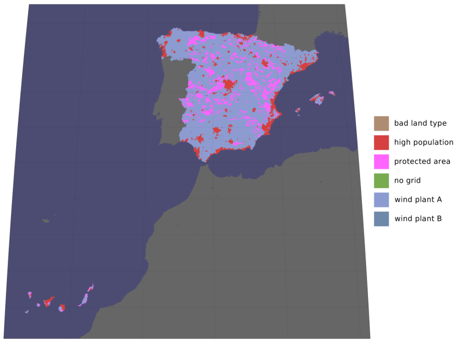

(p) Onshore wind mask for Spain.

Fig 44. RES area masks for all exporting countries considered and onshore wind as well as PV. No differentiation was made between ‘plant A’ and ‘plant B’ locations in this study.
