# Supplementary material for: Import options for chemical energy carriers from renewable sources to Germany
Source: PLoS One. 2023 Feb 9;18(2):e0262340. doi: 10.1371/journal.pone.0281380 (PMC9910710; doi:10.1371/journal.pone.0281380)
Supplement: S1 Table — (PDF) [file pone.0281380.s008.pdf]

S 7 Table Tabular results: Levelised Cost of Energy

Table 5. Obtained Levelised Cost of Energy (LCoE) for all Energy Supply Chains (ESCs), exporting countries and 5 % p.a. as well as 10 % p.a. WACC.

ESC	year	WACC [% p.a.]	EUR/MWh_{LHV} AR	AU	DE	DK	EG	ES	MA	SA
pipeline-ch4	2030	5	120.36	151.42	139.76	95.56	87.33	93.60	92.42	94.01
	2030	10	177.03	224.37	213.60	139.92	128.07	137.57	135.71	138.14
	2040	5	103.28	129.78	119.39	79.60	69.88	74.59	72.45	74.01
	2040	10	152.72	193.13	178.38	116.17	102.15	109.46	106.02	108.53
	2050	5	93.04	116.67	106.37	71.67	58.44	63.63	60.69	62.24
	2050	10	138.51	174.70	159.81	105.19	85.96	93.74	89.34	91.85
pipeline-h2	2030	5	108.14	138.71	66.90	49.61	54.24	54.70	58.93	62.53
	2030	10	163.24	210.11	103.70	75.06	82.57	83.15	89.62	95.20
	2040	5	94.98	121.35	56.60	39.81	44.05	43.41	45.96	49.35
	2040	10	145.80	186.80	86.66	60.86	67.49	66.28	70.37	75.74
	2050	5	86.81	109.88	51.19	35.54	37.15	37.09	38.66	41.78
	2050	10	135.68	172.35	78.67	54.61	57.79	57.53	60.17	65.27
shipping-ftfuel	2030	5	129.18	165.87	336.38	214.27	141.25	173.33	147.31	139.32
	2030	10	185.13	236.93	517.53	312.28	203.41	252.72	212.54	200.43
	2040	5	106.71	137.86	287.71	179.32	109.51	136.36	114.01	107.79
	2040	10	153.22	196.21	430.63	260.49	157.00	198.08	164.06	154.48
	2050	5	92.58	120.35	256.49	159.66	90.61	115.87	95.12	89.72
	2050	10	133.59	161.81	385.18	232.87	130.49	169.08	137.49	129.17
shipping-lch4	2030	5	70.22	82.44	145.16	96.83	79.01	90.66	82.21	82.16
	2030	10	101.46	119.88	221.56	141.51	115.01	132.76	119.85	119.66
	2040	5	58.92	69.32	124.47	81.06	63.14	72.44	64.33	64.79
	2040	10	85.43	100.95	185.81	118.04	91.52	105.82	93.39	94.01
	2050	5	52.19	61.35	111.31	73.27	52.75	62.04	53.73	54.45
	2050	10	76.06	89.70	166.74	107.26	76.84	90.87	78.36	79.33

Table 5 (continued).

ESC	year	WACC [% p.a.]	EUR/MWh$_{LHV}$ AR	AU	DE	DK	EG	ES	MA	SA
shipping-lh2	2030	5	67.72	86.61	109.72	77.73	69.59	76.31	69.92	79.09
	2030	10	97.12	124.05	165.98	113.63	101.07	112.11	102.24	114.30
	2040	5	57.95	75.53	95.60	64.58	56.87	61.09	54.72	63.90
	2040	10	83.14	107.97	142.17	94.25	82.58	89.75	79.99	92.08
	2050	5	51.84	68.54	85.21	57.38	47.81	52.57	45.30	54.99
	2050	10	74.56	98.00	127.22	84.06	69.56	77.55	66.49	79.23
shipping-lnh3	2030	5	64.09	78.39	131.35	87.44	71.13	82.02	73.69	76.32
	2030	10	94.89	116.27	203.86	130.01	105.98	122.58	109.86	113.50
	2040	5	53.55	65.92	112.52	72.06	56.87	64.79	57.35	60.05
	2040	10	79.51	97.85	170.29	106.99	84.61	96.85	85.46	89.16
	2050	5	46.90	57.94	99.37	63.98	46.95	54.62	46.93	49.92
	2050	10	69.84	86.16	150.84	95.35	70.06	81.85	70.19	74.32
shipping-lohc	2030	5	72.06	100.08	113.52	76.33	69.32	74.52	66.62	79.29
	2030	10	106.21	147.26	174.02	114.25	103.08	111.30	99.14	116.97
	2040	5	62.98	89.44	100.32	64.84	56.10	60.87	51.63	65.01
	2040	10	92.85	131.36	151.70	96.25	82.85	91.54	76.70	95.55
	2050	5	59.05	84.51	93.23	60.80	49.39	55.40	44.94	58.30
	2050	10	87.05	124.03	141.18	90.52	73.07	82.25	66.90	85.64
shipping-meoh	2030	5	91.21	112.10	239.80	143.90	100.64	123.95	105.13	99.68
	2030	10	130.87	162.58	372.17	209.31	145.20	181.05	151.94	143.53
	2040	5	76.03	93.26	208.01	123.06	78.73	97.61	81.53	77.81
	2040	10	109.26	134.86	312.47	178.82	113.22	142.16	117.67	111.67
	2050	5	66.53	81.44	186.72	111.91	65.43	83.22	68.27	65.07
	2050	10	96.01	118.37	281.32	163.25	94.49	121.71	98.96	93.80
