# Supplementary material for: Import options for chemical energy carriers from renewable sources to Germany
Source: PLoS One. 2023 Feb 9;18(2):e0262340. doi: 10.1371/journal.pone.0281380 (PMC9910710; doi:10.1371/journal.pone.0281380)
Supplement: S2 Table — (PDF) [file pone.0281380.s009.pdf]

S 8 Table Tabular results: Levelised Cost of Hydrogen

Table 6. Obtained Levelised Cost of Hydrogen (LCoH) for all Energy Supply Chains (ESCs), exporting countries and 5 % p.a. as well as 10 % p.a. WACC.

ESC	year	WACC	EUR/kg_{H2}							
		[% p.a.]	AR	AU	DE	DK	EG	ES	MA	SA
hvdc	2030	5	5.54	7.11	2.51	1.99	2.50	2.32	2.75	2.95
	2030	10	8.68	11.13	3.93	3.03	3.85	3.56	4.24	4.55
	2040	5	5.08	6.53	2.19	1.66	2.20	1.96	2.36	2.56
	2040	10	7.99	10.23	3.37	2.54	3.39	3.02	3.63	3.95
	2050	5	4.77	6.12	2.00	1.52	1.95	1.73	2.04	2.25
	2050	10	7.51	9.60	3.09	2.33	3.02	2.66	3.16	3.48
pipeline-ch4	2030	5	6.19	7.73	7.41	5.13	4.53	4.91	4.79	4.87
	2030	10	9.10	11.45	11.24	7.46	6.63	7.21	7.01	7.14
	2040	5	5.33	6.66	6.32	4.35	3.64	3.93	3.77	3.84
	2040	10	7.88	9.89	9.42	6.35	5.31	5.76	5.52	5.63
	2050	5	4.82	6.00	5.65	3.94	3.07	3.38	3.19	3.25
	2050	10	7.17	8.96	8.46	5.77	4.50	4.97	4.69	4.79
pipeline-h2	2030	5	3.60	4.62	2.23	1.65	1.81	1.82	1.96	2.08
	2030	10	5.44	7.00	3.46	2.50	2.75	2.77	2.99	3.17
	2040	5	3.17	4.04	1.89	1.33	1.47	1.45	1.53	1.64
	2040	10	4.86	6.23	2.89	2.03	2.25	2.21	2.35	2.52
	2050	5	2.89	3.66	1.71	1.18	1.24	1.24	1.29	1.39
	2050	10	4.52	5.75	2.62	1.82	1.93	1.92	2.01	2.18
shipping-ftfuel	2030	5	4.31	5.53	11.21	7.14	4.71	5.78	4.91	4.64
	2030	10	6.17	7.90	17.25	10.41	6.78	8.42	7.08	6.68
	2040	5	3.56	4.60	9.59	5.98	3.65	4.55	3.80	3.59
	2040	10	5.11	6.54	14.35	8.68	5.23	6.60	5.47	5.15
	2050	5	3.09	4.01	8.55	5.32	3.02	3.86	3.17	2.99
	2050	10	4.45	5.39	12.84	7.76	4.35	5.64	4.58	4.31

Table 6 (continued).

ESC	year	WACC [% p.a.]	AR	AU	DE	DK	EG	ES	MA	SA
			EUR/kg_{H2}							
shipping-lch4	2030	5	3.67	4.30	7.60	5.12	4.08	4.71	4.25	4.26
	2030	10	5.30	6.24	11.52	7.45	5.94	6.90	6.18	6.20
	2040	5	3.10	3.63	6.48	4.35	3.30	3.79	3.36	3.38
	2040	10	4.49	5.28	9.66	6.34	4.78	5.53	4.87	4.90
	2050	5	2.77	3.23	5.81	3.94	2.78	3.26	2.83	2.86
	2050	10	4.03	4.72	8.67	5.77	4.04	4.78	4.12	4.17
shipping-lh2	2030	5	2.26	2.89	3.66	2.59	2.32	2.54	2.33	2.64
	2030	10	3.24	4.13	5.53	3.79	3.37	3.74	3.41	3.81
	2040	5	1.93	2.52	3.19	2.15	1.90	2.04	1.82	2.13
	2040	10	2.77	3.60	4.74	3.14	2.75	2.99	2.67	3.07
	2050	5	1.73	2.28	2.84	1.91	1.59	1.75	1.51	1.83
	2050	10	2.49	3.27	4.24	2.80	2.32	2.59	2.22	2.64
shipping-lnh3	2030	5	3.60	4.32	7.06	4.83	3.93	4.52	4.06	4.21
	2030	10	5.27	6.35	10.79	7.10	5.79	6.69	5.99	6.20
	2040	5	2.97	3.58	5.93	4.00	3.12	3.54	3.14	3.28
	2040	10	4.36	5.27	8.91	5.89	4.59	5.23	4.63	4.82
	2050	5	2.53	3.07	5.15	3.47	2.51	2.91	2.52	2.66
	2050	10	3.73	4.53	7.76	5.13	3.72	4.33	3.73	3.93
shipping-lohc	2030	5	2.40	3.34	3.78	2.54	2.31	2.48	2.22	2.64
	2030	10	3.54	4.91	5.80	3.81	3.44	3.71	3.30	3.90
	2040	5	2.10	2.98	3.34	2.16	1.87	2.03	1.72	2.17
	2040	10	3.09	4.38	5.06	3.21	2.76	3.05	2.56	3.18
	2050	5	1.97	2.82	3.11	2.03	1.65	1.85	1.50	1.94
	2050	10	2.90	4.13	4.71	3.02	2.44	2.74	2.23	2.85
shipping-meoh	2030	5	3.68	4.54	9.71	6.02	4.04	5.00	4.22	4.01
	2030	10	5.29	6.57	15.01	8.79	5.83	7.31	6.10	5.77
	2040	5	3.07	3.76	8.39	5.11	3.16	3.94	3.28	3.12

Table 6 (continued).

ESC	year	WACC [% p.a.]	EUR/kg_{H2} AR	AU	DE	DK	EG	ES	MA	SA
	2040	10	4.41	5.44	12.59	7.44	4.54	5.74	4.73	4.48
	2050	5	2.69	3.29	7.53	4.61	2.63	3.36	2.74	2.61
	2050	10	3.88	4.77	11.34	6.73	3.79	4.91	3.98	3.77
