# Supplementary material for: Import options for chemical energy carriers from renewable sources to Germany
Source: PLoS One. 2023 Feb 9;18(2):e0262340. doi: 10.1371/journal.pone.0281380 (PMC9910710; doi:10.1371/journal.pone.0281380)
Supplement: S3 Table — (PDF) [file pone.0281380.s010.pdf]

S 9 Table Technology assumptions

All technology costs are given in EUR2015. Inflation adjustment was done where necessary assuming a 2 % p.a. inflation rate.

Table 7. Technology cost and lifetime assumptions used for 2030/2040/2050. A machine readable version of the input assumption can be found in the Zenodo and GitHub repositories listed in the data availability section.

technology	parameter	year	value	unit	source
direct air capture	CAPEX	2030	6000000.0	EUR/(tCO2/h)	Danish Energy Agency, ../technology_data_for_industrial_process_heat_0002.xlsx
		2040	5000000.0		
		2050	4000000.0		
	lifetime	2030	20.0	years	Danish Energy Agency, ../technology_data_for_industrial_process_heat_0002.xlsx
		2040	20.0		
		2050	20.0		
	FOM	2030	4.95	%/year	Danish Energy Agency, ../technology_data_for_industrial_process_heat_0002.xlsx
		2040	4.95		
		2050	4.95		
CO2 liquefaction	CAPEX	2030	16.03	EUR/t_CO2/h	Mitsubish Heavy Industries Ltd. and IEA (2004): https://ieaghg.org/docs/General_Docs/Reports/PH4-30%20Ship%20Transport.pdf .
		2040	16.03		
		2050	16.03		
	lifetime	2030	25.0	years	Guesstimate, based on CH4 liquefaction.
		2040	25.0		
		2050	25.0		
	FOM	2030	5.0	%/year	Mitsubish Heavy Industries Ltd. and IEA (2004): https://ieaghg.org/docs/General_Docs/Reports/PH4-30%20Ship%20Transport.pdf .
		2040	5.0		
		2050	5.0		
methanolisation	CAPEX	2030	3008.96	EUR/kW_MeOH	Danish Energy Agency, ../data_sheets_for_renewable_fuels.xlsx
		2040	2256.72		
		2050	1504.48		
		2030	20.0		
	lifetime			years	Danish Energy Agency, ../data_sheets_for_renewable_fuels.xlsx

Table 7 (continued).

technology	parameter	year	value	unit	source
		2040	20.0		
		2050	20.0		
		2030	1.75		
	FOM	2040	2.33	%/year	Danish Energy Agency, ../data_sheets_for_renewable_fuels.xlsx
		2050	3.5		
battery storage		2030	142.0		
	CAPEX			EUR/kWh	Danish Energy Agency, ../technology_data_catalogue_for_energy_storage.xlsx
		2040	94.0		
		2050	75.0		
		2030	25.0		
	lifetime	2040	30.0	years	Danish Energy Agency, ../technology_data_catalogue_for_energy_storage.xlsx
		2050	30.0		
	FOM	-	-		
seawater desalination		2030	32882.05		Caldera et al 2017: Learning Curve for Seawater Reverse Osmosis Desalination
	CAPEX			EUR/(m3-H2O/h)	Plants: Capital Cost Trend of the Past, Present, and Future
		2040	26297.44		(https://doi.org/10.1002/2017WR021402), Table 4.
		2050	21025.64		
		2030	30.0		Caldera et al 2016: Local cost of seawater RO desalination based on solar PV
	lifetime	2040	30.0	years	and windenergy: A global estimate.
		2050	30.0		(https://doi.org/10.1016/j.desal.2016.02.004), Table 1.
		2030	4.0		Caldera et al 2016: Local cost of seawater RO desalination based on solar PV
	FOM	2040	4.0	%/year	and windenergy: A global estimate.
		2050	4.0		(https://doi.org/10.1016/j.desal.2016.02.004), Table 1.
methanation		2030	278.0		
	CAPEX	2040	226.0	EUR/kW(CH4)	Fasihi et al 2017, table 1, https://www.mdpi.com/2071-1050/9/2/306
		2050	226.0		
		2030	30.0		
	lifetime	2040	30.0	years	Fasihi et al 2017, table 1, https://www.mdpi.com/2071-1050/9/2/306

Table 7 (continued).

technology	parameter	year	value	unit	source
		2050	30.0		
	FOM	2030	4.0	%/year	Fasihi et al 2017, table 1, https://www.mdpi.com/2071-1050/9/2/306
		2040	4.0		
		2050	4.0		
H2 (g) submarine pipeline	CAPEX	2030	329.37	EUR/MW/km	Assume similar cost as for CH4 (g) submarine pipeline but with the same factor as between onland CH4 (g) pipeline and H2 (g) pipeline (2.86). This estimate is comparable to a 36in diameter pipeline calaculated based on d'Amore-Domenech et al (2021): 10.1016/j.apenergy.2021.116625 , supplementary material (=251 EUR/MW/km).
		2040	329.37		
		2050	329.37		
	lifetime	2030	30.0	years	Assume same as for CH4 (g) submarine pipeline.
		2040	30.0		
		2050	30.0		
	FOM	2030	3.0	%/year	Assume same as for CH4 (g) submarine pipeline.
		2040	3.0		
		2050	3.0		
General liquid hydrocarbon storage (product)	CAPEX	2030	169.79	EUR/m3	Stelter and Nishida 2013: https://webstore.iea.org/insights-series-2013-focus-on-energy-security , pg. 8F .
		2040	169.79		
		2050	169.79		
	lifetime	2030	30.0	years	Stelter and Nishida 2013: https://webstore.iea.org/insights-series-2013-focus-on-energy-security , pg. 11.
		2040	30.0		
		2050	30.0		
	FOM	2030	6.25	%/year	Stelter and Nishida 2013: https://webstore.iea.org/insights-series-2013-focus-on-energy-security , figure 7 and pg. 12 .
		2040	6.25		
		2050	6.25		
CH4 evaporation	CAPEX	2030	0.28	EUR/kW(CH4)	Calculated, based on Fasihi et al 2017, table 1, https://www.mdpi.com/2071-1050/9/2/306

Table 7 (continued).

technology	parameter	year	value	unit	source
		2040	0.28		
		2050	0.28		
		2030	30.0		
	lifetime	2040	30.0	years	Fasihi et al 2017, table 1, https://www.mdpi.com/2071-1050/9/2/306
		2050	30.0		
		2030	3.5		
	FOM	2040	3.5	%/year	Fasihi et al 2017, table 1, https://www.mdpi.com/2071-1050/9/2/306
		2050	3.5		
Fischer-Tropsch		2030	1600.0		
	CAPEX			EUR/kW_FT/year	Danish Energy Agency, ../data_sheets_for_renewable_fuels.xlsx
		2040	1100.0		
		2050	900.0		
		2030	25.0		
	lifetime	2040	25.0	years	Danish Energy Agency, ../data_sheets_for_renewable_fuels.xlsx
		2050	25.0		
		2030	3.0		
	FOM	2040	3.0	%/year	doi:10.3390/su9020306
		2050	3.0		
LNG storage tank		2030	611.59		
	CAPEX			EUR/m3	Hurskainen 2019, https://cris.vtt.fi/en/publications/liquid-organic-hydrogen-carriers-lohc-concept-evaluation-and-tech pg. 46 (59).
		2040	611.59		
		2050	611.59		
		2030	20.0		
	lifetime	2040	20.0	years	Guesstimate, based on H2 (l) storage tank with comparable requirements.
		2050	20.0		
		2030	2.0		
	FOM	2040	2.0	%/year	Guesstimate, based on H2 (l) storage tank with comparable requirements.
		2050	2.0		

Table 7 (continued).

technology	parameter	year	value	unit	source
H2 (l) storage tank	CAPEX	2030	750.08	EUR/MWh(H2)	Reuß et al 2017, https://doi.org/10.1016/j.apenergy.2017.05.050 , Table 6.
		2040	750.08		
		2050	750.08		
	lifetime	2030	20.0	years	Reuß et al 2017, https://doi.org/10.1016/j.apenergy.2017.05.050 , Table 6.
		2040	20.0		
		2050	20.0		
	FOM	2030	2.0	%/year	Reuß et al 2017, https://doi.org/10.1016/j.apenergy.2017.05.050 , Table 6.
		2040	2.0		
		2050	2.0		
LOHC unloaded DBT storage	CAPEX	2030	132.26	EUR/t	Density via Wissenschaftliche Dienste des Deutschen Bundestages 2020, https://www.bundestag.de/resource/blob/816048/454e182d5956d45a664da9eb85486f76/WD-8-058-20-pdf-data.pdf , pg. 11.
		2040	132.26		
		2050	132.26		
	lifetime	2030	30.0	years	nan
		2040	30.0		
		2050	30.0		
	FOM	2030	6.25	%/year	nan
		2040	6.25		
		2050	6.25		
battery inverter	CAPEX	2030	160.0	EUR/kW	Danish Energy Agency, ../technology_data_catalogue_for_energy_storage.xlsx
		2040	100.0		
		2050	60.0		
	lifetime	2030	10.0	years	Danish Energy Agency, ../technology_data_catalogue_for_energy_storage.xlsx, Note K.
		2040	10.0		
		2050	10.0		

Table 7 (continued).

technology	parameter	year	value	unit	source
		2030	0.34		
	FOM	2040	0.54	%/year	Danish Energy Agency, ../technology_data_catalogue_for_energy_storage.xlsx
		2050	0.9		
Methanol steam reforming	CAPEX	2030	16318.43	EUR/MW(H2)	Niermann et al (2021): 10.1016/j.rser.2020.110171 , table 4.
		2040	16318.43		
		2050	16318.43		
		2030	20.0		
	lifetime	2040	20.0	years	Niermann et al (2021): 10.1016/j.rser.2020.110171 , table 4.
		2050	20.0		
		2030	4.0		
	FOM	2040	4.0	%/year	Niermann et al (2021): 10.1016/j.rser.2020.110171 , table 4.
		2050	4.0		
CO2 storage tank	CAPEX	2030	2528.17	EUR/t_CO2	Lauri et al. 2014: doi: 10.1016/j.egypro.2014.11.297, Table 3.
		2040	2528.17		
		2050	2528.17		
		2030	25.0		
	lifetime	2040	25.0	years	Lauri et al. 2014: doi: 10.1016/j.egypro.2014.11.297, pg. 2746 .
		2050	25.0		
		2030	1.0		
	FOM	2040	1.0	%/year	Lauri et al. 2014: doi: 10.1016/j.egypro.2014.11.297, pg. 2746 .
		2050	1.0		
LOHC hydrogenation	CAPEX	2030	46471.24	EUR/MW(H2)	Runge et al 2020, pg.8, https://papers.ssrn.com/abstract=3623514
		2040	46471.24		
		2050	46471.24		

Table 7 (continued).

technology	parameter	year	value	unit	source
	lifetime	2030	20.0	years	Runge et al 2020, pg.8, https://papers.ssrn.com/abstract=3623514
		2040	20.0		
		2050	20.0		
	FOM	2030	3.0	%/year	Runge et al 2020, pg.8, https://papers.ssrn.com/abstract=3623514
		2040	3.0		
		2050	3.0		
H2 (g) pipeline	CAPEX	2030	226.47	EUR/MW/km	European Hydrogen Backbone Report (June 2021): https://gasforclimate2050.eu/wp-content/uploads/2021/06/EHB_Analysing-the-future-demand-supply-and-transport-of-hydrogen_June-2021.pdf.
		2040	226.47		
		2050	226.47		
	lifetime	2030	50.0	years	Danish Energy Agency, Technology Data for Energy Transport (2021), Excel datasheet: H2 140.
		2040	50.0		
		2050	50.0		
	FOM	2030	3.17	%/year	Danish Energy Agency, Technology Data for Energy Transport (2021), Excel datasheet: H2 140.
		2040	2.33		
		2050	1.5		
industrial heat pump medium temperature	CAPEX	2030	778.8	EUR/kW	Danish Energy Agency, ../technology_data_for_industrial_process_heat_0002.xlsx
		2040	730.0		
		2050	700.0		
	lifetime	2030	20.0	years	Danish Energy Agency, ../technology_data_for_industrial_process_heat_0002.xlsx
		2040	20.0		
		2050	20.0		
	FOM	2030	0.11	%/year	Danish Energy Agency, ../technology_data_for_industrial_process_heat_0002.xlsx
		2040	0.11		
		2050	0.1		
CH4 (g) pipeline	CAPEX	2030	79.0	EUR/MW/km	Guesstimate.

Table 7 (continued).

technology	parameter	year	value	unit	source
		2040	79.0		
		2050	79.0		
		2030	50.0		
	lifetime	2040	50.0	years	Assume same as for H2 (g) pipeline in 2050 (CH4 pipeline as mature technology).
		2050	50.0		
		2030	1.5		
	FOM	2040	1.5	%/year	Assume same as for H2 (g) pipeline in 2050 (CH4 pipeline as mature technology).
		2050	1.5		
CCGT		2030	830.0		
	CAPEX	2040	815.0	EUR/kW	Danish Energy Agency, ../technology_data_for(el)_and_dh.xlsx
		2050	800.0		
		2030	25.0		
	lifetime	2040	25.0	years	Danish Energy Agency, ../technology_data_for(el)_and_dh.xlsx
		2050	25.0		
		2030	3.35		
	FOM	2040	3.3	%/year	Danish Energy Agency, ../technology_data_for(el)_and_dh.xlsx
		2050	3.25		
CH4 liquefaction		2030	0.74		
	CAPEX			EUR/kW(CH4)	Calculated, based on Fasihi et al 2017, table 1, https://www.mdpi.com/2071-1050/9/2/306
		2040	0.74		
		2050	0.74		
		2030	25.0		
	lifetime	2040	25.0	years	Fasihi et al 2017, table 1, https://www.mdpi.com/2071-1050/9/2/306
		2050	25.0		
		2030	3.5		
	FOM	2040	3.5	%/year	Fasihi et al 2017, table 1, https://www.mdpi.com/2071-1050/9/2/306
		2050	3.5		

Table 7 (continued).

technology	parameter	year	value	unit	source
clean water tank storage	CAPEX	2030	67.63	EUR/m3-H2O	Caldera et al 2016: Local cost of seawater RO desalination based on solar PV and windenergy: A global estimate. (https://doi.org/10.1016/j.desal.2016.02.004), Table 1.
		2040	67.63		
		2050	67.63		
	lifetime	2030	30.0	years	Caldera et al 2016: Local cost of seawater RO desalination based on solar PV and windenergy: A global estimate. (https://doi.org/10.1016/j.desal.2016.02.004), Table 1.
		2040	30.0		
		2050	30.0		
	FOM	2030	2.0	%/year	Caldera et al 2016: Local cost of seawater RO desalination based on solar PV and windenergy: A global estimate. (https://doi.org/10.1016/j.desal.2016.02.004), Table 1.
		2040	2.0		
		2050	2.0		
HVDC inverter pair	CAPEX	2030	150000.0	EUR/MW	Hagspiel
		2040	150000.0		
		2050	150000.0		
	lifetime	2030	40.0	years	Hagspiel
		2040	40.0		
		2050	40.0		
	FOM	2030	2.0	%/year	Hagspiel
		2040	2.0		
		2050	2.0		
Ammonia cracker	CAPEX	2030	1400083.71	EUR/MW(H2)	ENGIE et al (2020): Ammonia to Green Hydrogen Feasibility Study (https://assets.publishing.service.gov.uk/government/uploads/system/uploads/attachment_data/file/880826/HS420_-_Ecuity_-_Ammonia_to_Green_Hydrogen.pdf).
		2040	1400083.71		
		2050	1400083.71		
	lifetime	2030	30.0	years	-
		2040	30.0		
		2050	30.0		
	FOM	2030	3.0	%/year	-

Table 7 (continued).

technology	parameter	year	value	unit	source
		2040	3.0		
		2050	3.0		
LOHC loaded DBT storage	CAPEX	2030	149.27	EUR/t	Density via Wissenschaftliche Dienste des Deutschen Bundestages 2020, https://www.bundestag.de/resource/blob/816048/454e182d5956d45a664da9eb85486f76/WD-8-058-20-pdf-data.pdf , pg. 11.
		2040	149.27		
		2050	149.27		
	lifetime	2030	30.0	years	nan
		2040	30.0		
		2050	30.0		
	FOM	2030	6.25	%/year	nan
		2040	6.25		
		2050	6.25		
hydrogen storage tank incl. compressor	CAPEX	2030	44.91	EUR/kWh	Danish Energy Agency, ../technology_data_catalogue_for_energy_storage.xlsx
		2040	27.05		
		2050	21.0		
	lifetime	2030	30.0	years	Danish Energy Agency, ../technology_data_catalogue_for_energy_storage.xlsx
		2040	30.0		
		2050	30.0		
	FOM	2030	1.11	%/year	Danish Energy Agency, ../technology_data_catalogue_for_energy_storage.xlsx
		2040	1.85		
		2050	1.9		
electrolysis	CAPEX	2030	450.0	EUR/kW_e	Danish Energy Agency, ../data_sheets_for_renewable_fuels.xlsx
		2040	300.0		
		2050	250.0		
	lifetime	2030	30.0	years	Danish Energy Agency, ../data_sheets_for_renewable_fuels.xlsx
		2040	32.0		

Table 7 (continued).

technology	parameter	year	value	unit	source
		2050	35.0		
	FOM	2030	2.0	%/year	Danish Energy Agency, ../data_sheets_for_renewable_fuels.xlsx
		2040	2.0		
		2050	2.0		
methane storage tank incl. compressor	CAPEX	2030	8629.2	EUR/m3	Storage costs per l: https://www.compositesworld.com/articles/pressure-vessels-for-alternative-fuels-2014-2023 (2021-02-10).
		2040	8629.2		
		2050	8629.2		
	lifetime	2030	30.0	years	Guesstimate, based on hydrogen storage tank by DEA.
		2040	30.0		
		2050	30.0		
	FOM	2030	1.9	%/year	Guesstimate, based on hydrogen storage tank by DEA.
		2040	1.9		
		2050	1.9		
air separation unit	CAPEX	2030	10942205.1	EUR/MW	Calculated based on Morgan E. 2013: doi:10.7275/11KT-3F59 , Fig. 56, Fig. 58, pg. 207, pg. 210.
		2040	10942205.1		
		2050	10942205.1		
	lifetime	2030	20.0	years	Morgan E. 2013: doi:10.7275/11KT-3F59 , pg. 290
		2040	20.0		
		2050	20.0		
	FOM	2030	4.0	%/year	Estimate, based on methanation plant.
		2040	4.0		
		2050	4.0		
H2 evaporation	CAPEX	2030	4320.43	EUR/MW(H2)	Reuß et al 2017: https://doi.org/10.1016/j.apenergy.2017.05.050 , Table 9 and equation in sec 3.0.
		2040	4320.43		

Table 7 (continued).

technology	parameter	year	value	unit	source
		2050	4320.43		
	lifetime	2030	10.0	years	Reuß et al 2017: https://doi.org/10.1016/j.apenergy.2017.05.050 , Table 9 and equation in sec 3.0.
		2040	10.0		
		2050	10.0		
	FOM	2030	3.0	%/year	Reuß et al 2017: https://doi.org/10.1016/j.apenergy.2017.05.050 , Table 9 and equation in sec 3.0.
		2040	3.0		
		2050	3.0		
H2 (g) fill compressor station	CAPEX	2030	4478.0	EUR/MW(H2)	Danish Energy Agency, Technology Data for Energy Transport (2021), pg. 164, Figure 14 (Fill compressor).
		2040	4478.0		
		2050	4478.0		
	lifetime	2030	20.0	years	Danish Energy Agency, Technology Data for Energy Transport (2021), pg. 168, Figure 24 (Fill compressor).
		2040	20.0		
		2050	20.0		
	FOM	2030	1.7	%/year	Guidehouse 2020: European Hydrogen Backbone report, https://guidehouse.com/-/media/www/site/downloads/energy/2020/gh_european-hydrogen-backbone_report.pdf (table 3, table 5)
		2040	1.7		
		2050	1.7		
NH3 (l) storage tank incl. liquefaction	CAPEX	2030	161.93	EUR/MWh_NH3	Calculated based on Morgan E. 2013: doi:10.7275/11KT-3F59 , Fig. 55, Fig 58.
		2040	161.93		
		2050	161.93		
	lifetime	2030	20.0	years	Morgan E. 2013: doi:10.7275/11KT-3F59 , pg. 290
		2040	20.0		
		2050	20.0		
	FOM	2030	2.0	%/year	Guesstimate, based on H2 (l) storage tank.
		2040	2.0		
		2050	2.0		

Table 7 (continued).

technology	parameter	year	value	unit	source
Steam methane reforming	CAPEX	2030	470085.47	EUR/MW(H2)	International Energy Agency (2015): Technology Roadmap Hydrogen and Fuel Cells , table 15.
		2040	470085.47		
		2050	470085.47		
	lifetime	2030	30.0	years	International Energy Agency (2015): Technology Roadmap Hydrogen and Fuel Cells , table 15.
		2040	30.0		
		2050	30.0		
	FOM	2030	3.0	%/year	International Energy Agency (2015): Technology Roadmap Hydrogen and Fuel Cells , table 15.
		2040	3.0		
		2050	3.0		
CH4 (g) fill compressor station	CAPEX	2030	1498.95	EUR/MW(CH4)	Guesstimate, based on H2 (g) pipeline and fill compressor station cost.
		2040	1498.95		
		2050	1498.95		
	lifetime	2030	20.0	years	Assume same as for H2 (g) fill compressor station.
		2040	20.0		
		2050	20.0		
	FOM	2030	1.7	%/year	Assume same as for H2 (g) fill compressor station.
		2040	1.7		
		2050	1.7		
HVDC overhead	CAPEX	2030	400.0	EUR/MW/km	Hagspiel
		2040	400.0		
		2050	400.0		
	lifetime	2030	40.0	years	Hagspiel
		2040	40.0		
		2050	40.0		

Table 7 (continued).

technology	parameter	year	value	unit	source
	FOM	2030	2.0	%/year	Hagspiel
	FOM	2040	2.0	%/year	Hagspiel
	FOM	2050	2.0	%/year	Hagspiel
HVDC submarine	CAPEX	2030	471.16	EUR/MW/km	Purvins et al. (2018): https://doi.org/10.1016/j.jclepro.2018.03.095 .
	CAPEX	2040	471.16		
	CAPEX	2050	471.16		
	lifetime	2030	40.0	years	Purvins et al. (2018): https://doi.org/10.1016/j.jclepro.2018.03.095 .
	lifetime	2040	40.0		
	lifetime	2050	40.0		
	FOM	2030	0.35	%/year	Purvins et al. (2018): https://doi.org/10.1016/j.jclepro.2018.03.095 .
	FOM	2040	0.35		
	FOM	2050	0.35		
LOHC dehydrogenation	CAPEX	2030	759908.15	EUR/MW(H2)	Runge et al 2020, pg.8, https://papers.ssrn.com/abstract=3623514
	CAPEX	2040	759908.15		
	CAPEX	2050	759908.15		
	lifetime	2030	20.0	years	Runge et al 2020, pg.8, https://papers.ssrn.com/abstract=3623514
	lifetime	2040	20.0		
	lifetime	2050	20.0		
	FOM	2030	3.0	%/year	Runge et al 2020, pg.8, https://papers.ssrn.com/abstract=3623514
	FOM	2040	3.0		
	FOM	2050	3.0		
CH4 (g) submarine pipeline	CAPEX	2030	114.89	EUR/MW/km	Kaiser (2017): 10.1016/j.marpol.2017.05.003 .
	CAPEX	2040	114.89		
	CAPEX	2050	114.89		

Table 7 (continued).

technology	parameter	year	value	unit	source
	lifetime	2030	30.0	years	d'Amore-Domenech et al (2021): 10.1016/j.apenergy.2021.116625 , supplementary material.
		2040	30.0		
		2050	30.0		
	FOM	2030	3.0	%/year	d'Amore-Domenech et al (2021): 10.1016/j.apenergy.2021.116625 , supplementary material.
		2040	3.0		
		2050	3.0		
H2 liquefaction	CAPEX	2030	1497967.32	EUR/MW(H2)	Reuß et al 2017: https://doi.org/10.1016/j.apenergy.2017.05.050 , Table 9 and equation in sec 3.0.
		2040	1497967.32		
		2050	1497967.32		
	lifetime	2030	20.0	years	Reuß et al 2017: https://doi.org/10.1016/j.apenergy.2017.05.050 , Table 9 and equation in sec 3.0.
		2040	20.0		
		2050	20.0		
	FOM	2030	8.0	%/year	Reuß et al 2017: https://doi.org/10.1016/j.apenergy.2017.05.050 , Table 9 and equation in sec 3.0.
		2040	8.0		
		2050	8.0		
