# Supplementary material for: Import options for chemical energy carriers from renewable sources to Germany
Source: PLoS One. 2023 Feb 9;18(2):e0262340. doi: 10.1371/journal.pone.0281380 (PMC9910710; doi:10.1371/journal.pone.0281380)
Supplement: S4 Table — (PDF) [file pone.0281380.s011.pdf]

S 10 Table Conversion efficiencies

Table 8. Energy and feedstock efficiency assumptions used. A machine readable version of these assumptions can be found in the Zenodo and GitHub repositories listed in the data availability section.

process	year	from	amount	to	amount	details and source
Ammonia cracker	all	ammonia (g)	3191.00 MWh(LHV)	hydrogen (g)	2186.00 MWh(LHV)	Assuming a integrated 200t/d cracking and purification facility. Electricity demand (316 MWh per 2186 MWh(LHV) H2 output) is assumed to also be ammonia LHV input which seems a fair assumption as the facility has options for a higher degree of integration according to the report).. Source: ENGIE et al (2020): Ammonia to Green Hydrogen Feasibility Study (https://assets.publishing.service.gov.uk/government/uploads/system/uploads/attachment__data/file/880826/HS420__-__Ecuity__-__Ammonia__to__Green__Hydrogen.pdf), Fig. 10.
CCGT	all	hydrogen (g)	1.00 MWh(LHV)	electricity	0.58 MWh(el)	nan. Source: DEA technology data for electricity generation and district heating.
CH4 (g) fill compressor station	all	methane (g)	1.00 p.u.	methane (g) compressed	0.97 p.u.	Assume same value as for booster stations, need to provide initial pressure increase.. Source: Guesstimate.
CH4 (g) pipeline	all	methane (g) compressed	1.00 p.u.	methane (g) compressed submarine	0.97 p.u.	Per 1000km for losses and boosting. Based on 0.4%/100 miles estimate.. Source: Asia Pacific Energy Research Centre (2000): https://aperc.or.jp/file/2010/9/26/Natural__Gas__Infrastructure__Development__Northeast__Asia__2000.pdf , Table 49.
CH4 (g) pipeline decompressor	all	methane (g) compressed	1.00 p.u.	methane (g)	1.00 p.u.	Arbitrary componenet for degrading gas pipeline content to regular methane (g) bus.. Source: Guesstimate.

Table 8 (continued).

process	year	from	amount	to	amount	details and source
CH4 (g) submarine pipeline	all	methane (g) compressed submarine	1.00 p.u.	methane (g) compressed	0.97 p.u.	Per 1000km for losses and boosting. Based on 0.4%/100 miles estimate.. Source: Asia Pacific Energy Research Centre (2000): https://aperc.or.jp/file/2010/9/26/Natural_Gas_Infrastructure_Development_Northeast_Asia_2000.pdf , Table 49.
CH4 (l) storing and unstoring	all	methane (l)	0.42 t	methane (l) storage	1.00 m3	Auxiliary efficiency. Storage units are usually per m3, component for unit conversion. Based on density of 422.36 kg/m3.. Source: https://encyclopedia.airliquide.com/methane (2021-02-10).
CH4 evaporation	all	methane (l)	1.00 t	methane (g)	13.61 MWh(LHV)	98% efficiency.. Source: Pospíšil et al. 2019: https://doi.org/10.1016/j.rser.2018.09.027 , Fig. 14.
CH4 liquefaction	all	electricity	0.50 MWh	methane (l)	1.00 t	Assuming 0.5 MWh/t(CH4) for refigeration cycle based on Table 2 of source; cleaning of gas presumed unnecessary as it should be nearly pure CH4 (=SNG). Assuming energy required is only electricity which is for Table 3 in the source provided with efficiencies of 50% of LHV, making the numbers consistent with the numbers in Table 2.. Source: Pospíšil et al. 2019: https://doi.org/10.1016/j.rser.2018.09.027 , Table 2 and Table 3. Source 2: https://encyclopedia.airliquide.com/methane (2021-02-10).

Table 8 (continued).

process	year	from	amount	to	amount	details and source
CH4 liquefaction	all	methane (g)	13.89 MWh(LHV)	methane (l)	1.00 t	For refrigeration cycle, cleaning of gas presumed unnecessary as it should be nearly pure CH4 (=SNG). Assuming energy required is only electricity which is for Table 3 in the source provided with efficiencies of 50% of LHV, making the numbers consistent with the numbers in Table 2.. Source: Pospíšil et al. 2019: https://doi.org/10.1016/j.rser.2018.09.027 , Table 2 and Table 3.
CH4 storage compressor	all	electricity	0.19 MWh	methane (g) storage	1.00 m3	Assuming ca. 7.5% of HHV (55.50 MJ/kg) based on multi-stage adiabatic compression and density of 167.758 kg/m3 .. Source: e.g. Bossel and Eliasson, "Energy and the Hydrogen Economy", pg. 10f. (https://afdc.energy.gov/files/pdfs/hyd_economy_bossel(el)iasson.pdf , 10.02.2021)
CH4 storage compressor	all	methane (g)	2.33 MWh(LHV)	methane (g) storage	1.00 m3	Assuming methane density of 167.758 kg/m3 at 200 bar and LHV of 50 MJ/kg .. Source: https://www.unitrove.com/engineering/tools/gas/natural-gas-density (2021-02-10).
CH4 storage unstoring	all	methane (g) storage	1.00 m3	methane (g)	2.33 MWh(LHV)	Reverse connection (auxiliary object) for CH4 (g) compressed storage.. Source: https://www.unitrove.com/engineering/tools/gas/natural-gas-density (2021-02-10).
CO2 evaporation	all	CO2 (l)	1.00 t	CO2 (g)	1.00 t	nan. Source: nan

Table 8 (continued).

process	year	from	amount	to	amount	details and source
CO2 liquefaction	all	CO2 (g)	1.00 t	CO2 (l)	1.00 t	Assuming a pure, humid, low-pressure input stream. Neglecting possible gross-effects of CO2 which might be cycled for the cooling process.. Source: Mitsubish Heavy Industries Ltd. and IEA (2004): https://ieaghg.org/docs/General_Docs/Reports/PH4-30%20Ship%20Transport.pdf .
CO2 liquefaction	all	electricity	0.12 MWh	CO2 (l)	1.00 t	nan. Source: Mitsubish Heavy Industries Ltd. and IEA (2004): https://ieaghg.org/docs/General_Docs/Reports/PH4-30%20Ship%20Transport.pdf .
CO2 liquefaction	all	heat	0.01 MWh	CO2 (l)	1.00 t	For drying purposes.. Source: Mitsubish Heavy Industries Ltd. and IEA (2004): https://ieaghg.org/docs/General_Docs/Reports/PH4-30%20Ship%20Transport.pdf .
FT fuel storing and unstoring	all	FT fuel	9.80 MWh(LHV)	FT fuel storage	1.00 m3	Storage units are usually per m3, component for unit conversion. Based on FT fuel composition of 75% jet fuel/25% kerosene and a resulting density of 0.820 t/m3 = (0.75*0.820 + 0.25*0.821)t/m3 and LHV of 11.95 MWh/t = (0.75*11.95+0.25*11.95)MWh/t. Source: nan
Fischer-Tropsch	all	CO2 (g)	3.90 t	FT fuel	9.60 MWh(LHV)	Input per 1t FT fuels output. Product composition can be assumed to be 60% jet fuels/20% kerosene/20%LPG & fuel gases after upgrading, but we discard the LPG&fuel gas byproduct in this assumption – we're focusing on the liquid products. Liquid products both have LHVs of 11.95 Mwh/t.. Source: DEA technology data for Renewable fuels (2018): Hydrogen to Power.

Table 8 (continued).

process	year	from	amount	to	amount	details and source
Fischer-Tropsch	all	electricity	0.01 MWh	FT fuel	0.56 MWh(LHV)	Assuming limited CO2 conversion efficiency; can theoretically be improved upon. Output is 0.7 MWh product equivalent, of which we're neglecting 20% end products after upgrade: LPG and other fuel gases – we're only looking at liquid products here.. Source: DEA technology data for Renewable fuels (2018): Hydrogen to Power.
Fischer-Tropsch	all	hydrogen (g)	0.99 MWh(LHV)	FT fuel	0.56 MWh(LHV)	Output is 0.7 MWh product equivalent, we're neglecting 20% end products after upgrade: LPG and other fuel gases – we're only looking at liquid products here.. Source: DEA technology data for Renewable fuels (2018): Hydrogen to Power.
H2 (g) fill compressor station	all	hydrogen (g)	1.00 p.u.	hydrogen (g) compressed	0.98 p.u.	Is 2.1%/1000km. Assuming self-consumption of H2 for filling compressors.. Source: Danish Energy Agency, Technology Data for Energy Transport (2021), Excel datasheet: H2 140.
H2 (g) pipeline	all	hydrogen (g) compressed	1.00 p.u.	hydrogen (g) compressed submarine	0.98 p.u.	Is 2.1%/1000km. Assuming self-consumption of H2 for boosting, 6000 MW_HHV H2 pipeline.. Source: Danish Energy Agency, Technology Data for Energy Transport (2021), Excel datasheet: H2 140.
H2 (g) pipeline decompressor	all	hydrogen (g) compressed	1.00 p.u.	hydrogen (g)	1.00 p.u.	Arbitrary componenet for degrading gas pipeline content to regular hydrogen (g) bus.. Source: Guess.
H2 (g) submarine pipeline	all	hydrogen (g) compressed submarine	1.00 p.u.	hydrogen (g) compressed	0.98 p.u.	2.1%/1000km. Assumed per 1000km here including H2 consumption for booster stations for 6000 MW_HHV pipeline.. Source: Danish Energy Agency, Technology Data for Energy Transport (2021), Excel datasheet: H2 140.

Table 8 (continued).

process	year	from	amount	to	amount	details and source
H2 evaporation	all	hydrogen (l)	1.00 MWh(LHV)	hydrogen (g)	1.00 MWh(LHV)	nan. Source: Heuser et al. 2019: https://doi.org/10.1016/j.ijhydene.2018.12.156, table 1.
H2 evaporation	all	electricity	0.60 MWh	hydrogen (g)	33.33 MWh(LHV)	.6 kWh/kg(H2). Source: Heuser et al. 2019: https://doi.org/10.1016/j.ijhydene.2018.12.156, table 1.
H2 liquefaction	all	hydrogen (g)	33.33 MWh(LHV)	hydrogen (l)	32.78 MWh(LHV)	'= 1.65% losses.. Source: Heuser et al. 2019: https://doi.org/10.1016/j.ijhydene.2018.12.156, table 1.
H2 liquefaction	all	electricity	6.78 MWh	hydrogen (l)	33.33 MWh(LHV)	6.78 kWh/kg(H2), considering H2 with LHV of 33.3333 MWh/t. Source: Heuser et al. 2019: https://doi.org/10.1016/j.ijhydene.2018.12.156, table 1.
H2 storage compressor	all	hydrogen (g)	1.00 MWh(LHV)	hydrogen (g) storage	1.00 MWh(LHV)	nan. Source: nan
H2 storage compressor	all	electricity	4.00 MWh	hydrogen (g) storage	33.33 MWh(LHV)	Storing into low pressure (<200 bar) type I tanks.. Source: DEA technology data for energy storage, pg. 82 .
H2 storage unstoring	all	hydrogen (g) storage	1.00 MWh(LHV)	hydrogen (g)	1.00 MWh(LHV)	Reverse connection (auxiliary object) for H2 (g) compressed storage.. Source: nan
HVDC inverter pair	all	hvdc	1.00 p.u.	electricity	0.99 p.u.	Losses for voltage source converters (VSCs). Line commutated converters (LLC) would be lower 0.7%.. Source: d'Amore-Domenech et al (2021): 10.1016/j.apenergy.2021.116625 .
HVDC inverter pair	all	electricity	1.00 p.u.	hvdc	0.99 p.u.	Losses for voltage source converters (VSCs). Line commutated converters (LLC) would be lower 0.7%.. Source: d'Amore-Domenech et al (2021): 10.1016/j.apenergy.2021.116625 .
HVDC overhead	all	hvdc	1.00 p.u.	hvdc submarine	0.98 p.u.	Per 1000 km HVDC submarine line. Based on 7% losses for 3000 km.. Source: Purvins et al. (2018): https://doi.org/10.1016/j.jclepro.2018.03.095 .

Table 8 (continued).

process	year	from	amount	to	amount	details and source
HVDC submarine	all	hvdc submarine	1.00 p.u.	hvdc	0.98 p.u.	Per 1000 km HVDC submarine line. Based on 7% losses for 3000 km.. Source: Purvins et al. (2018): https://doi.org/10.1016/j.jclepro.2018.03.095 .
Haber-Bosch	all	hydrogen (g)	5.93 MWh(LHV)	ammonia (g)	5.17 MWh(LHV)	178 kg(H2) per t_NH3, LHV for both assumed.. Source: DECHEMA 2017: DECHEMA: Low carbon energy and feedstock for the European chemical industry , pg. 57.
Haber-Bosch	all	electricity	1.28 MWh	ammonia (g)	5.17 MWh(LHV)	Assume 5 GJ/t_NH3 for compressors and NH3 LHV = 5.16666 MWh/t_NH3.. Source: DECHEMA 2017: DECHEMA: Low carbon energy and feedstock for the European chemical industry , table 11.
Haber-Bosch	all	nitrogen (g)	0.82 t(N2)	ammonia (g)	5.17 MWh(LHV)	.33 MWh electricity are required for ASU per t_NH3, considering 0.4 MWh are required per t(N2) and LHV of NH3 of 5.1666 Mwh.. Source: DECHEMA 2017: DECHEMA: Low carbon energy and feedstock for the European chemical industry , pg. 57.
LOHC (used) loading	all	LOHC (used)	1.00 p.u.	berth LOHC (used)	1.00 p.u.	Auxiliary process: Unloading / loading of LOHC (loaded/unloaded) happen at the same time, assume no losses as handling of LOHC is rather simple.. Source: nan
LOHC (used) unloading	all	berth LOHC (used)	1.00 p.u.	LOHC (used)	1.00 p.u.	Auxiliary process: Unloading / loading of LOHC (loaded/unloaded) happen at the same time, assume no losses as handling of LOHC is rather simple.. Source: nan

Table 8 (continued).

process	year	from	amount	to	amount	details and source
LOHC dehydrogenation	all	LOHC (loaded)	1.00 t	hydrogen (g)	1.34 MWh(LHV)	1t loaded LOHC (H18-DBT) contains 5.6 wt-% H2 (=0.056 t(H2)). Considering LHV and worst case assumption: Dehydrogenation heat has to be provided by burning hydrogen (no other heat source available, loss of H2 due to burning are 28.07%). The small electricity demand (power = 0.2 MW) from the source also already included, which seems reasonable as in the source the energy flow is missing 1 MW_HHV of hydrogen which is enough to provide this electricity).. Source: Niermann et al 2019: https://pubs.rsc.org/en/content/articlelanding/2019/ee/c8ee02700e , fig. 6A and for depth-of-discharge: Runge et al 2020, pg. 7, https://papers.ssrn.com/abstract=3623514 .
LOHC dehydrogenation	all	LOHC (loaded)	1.00 t	LOHC (used)	0.94 t	Considering 5.6 wt-% H2 in loaded LOHC and LHV of H2.. Source: Runge et al 2020, pg. 7, https://papers.ssrn.com/abstract=3623514
LOHC hydrogenation	all	LOHC (unloaded)	0.94 t	LOHC (loaded)	1.00 t	LOHC (DBT) has loaded only 5.6%-wt H2 as rate of discharge is kept at ca. 90%.. Source: nan
LOHC hydrogenation	all	electricity	0.20 MWh	LOHC (loaded)	51.66 t	Flow in figures shows 0.2 MW for 114 MW_HHV = 96.4326 MW(LHV) = 2.89298 t hydrogen. At 5.6 wt-% effective H2 storage for loaded LOHC (H18-DBT), corresponds to 51.6604 t loaded LOHC .. Source: Niermann et al 2019: https://pubs.rsc.org/en/content/articlelanding/2019/ee/c8ee02700e , fig. 6A .

Table 8 (continued).

process	year	from	amount	to	amount	details and source
LOHC hydrogenation	all	hydrogen (g)	1.87 MWh(LHV)	LOHC (loaded)	1.00 t	Considering 5.6 wt-% H2 in loaded LOHC and LHV of H2.. Source: Runge et al 2020, pg. 7, https://papers.ssrn.com/abstract=3623514
LOHC treatment	all	LOHC (used)	1.00 p.u.	LOHC (unloaded)	1.00 p.u.	A 0.01% penalty, as ca. 0.01% of LOHC have to be exchanged after each loading/unloading cycle.. Source: Runge et al 2020, pg. 7, https://papers.ssrn.com/abstract=3623514
MeOH storing and unstoring	all	methanol	4.38 MWh(LHV)	methanol storage	1.00 m3	Storage units are usually per m3, component for unit conversion. Based on LHV and density of 792 kg/m3 .. Source: nan
Methanol steam reforming	all	methanol	40.03 MWh(LHV)	hydrogen (g)	33.33 MWh(LHV)	Assuming per 1 t(H2) (with LHV 33.3333 MWh/t): 4.5 MWh(th) and 3.2 MWh(el) are required. We assume electricity can be substituted / provided with 1:1 as heat energy.. Source: Niermann et al (2021): 10.1016/j.rser.2020.110171 , table 4.
NH3 evaporation	all	ammonia (l)	1.00 p.u.	ammonia (g)	1.00 p.u.	guess. .. Source: nan
NH3 liquefaction	all	ammonia (g)	1.00 p.u.	ammonia (l)	1.00 p.u.	nan. Source: nan
NH3 liquefaction	all	electricity	0.00 p.u.	ammonia (l)	1.00 p.u.	0.1% of energy content of ammonia required for refrigeration.. Source: Dias et al. 2020: https://www.frontiersin.org/articles/10.3389/fmech.2020.00021/full
Steam methane reforming	all	methane (g)	123.61 MWh(LHV)	hydrogen (g)	83.33 MWh(LHV)	Large scale SMR plant producing 2.5 kg/s H2 output (assuming 33.3333 MWh/t H2 LHV), with 6.9 kg/s CH4 input (feedstock) and 2 kg/s CH4 input (energy). Neglecting water consumption.. Source: Keipi et al (2018): 10.1016/j.enconman.2017.12.063 , table 2.

Table 8 (continued).

process	year	from	amount	to	amount	details and source
air separation unit	all	electricity	0.25 MWh	nitrogen (g)	1.00 t(N2)	For consistency reasons use value from Danish Energy Agency. DEA also reports range of values (0.2-0.4 MWh/t(N2)) on pg. 288. Other efficienices reported are even higher, e.g. 0.11 Mwh/t(N2) from Morgan (2013): Techno-Economic Feasibility Study of Ammonia Plants Powered by Offshore Wind .. Source: Danish Energy Agency, Technology Data for Renewable Fuels (04/2022), pg.288 .
battery inverter	all	battery	1.00 MWh	electricity	0.98 MWh	from DEA technology assumptions via technology-data repository. Source: DEA
battery inverter	all	electricity	1.00 MWh	battery	0.98 MWh	from DEA technology assumptions via technology-data repository. Source: DEA
direct air capture	all	heat	1.60 MWh(th)	CO2 (g)	1.00 t	Thermal energy demand. Provided via air-sourced heat pumps. 1.6 MWh based on Beuttler et al (2019) for Climeworks LT DAC, alternative value: 1.102 MWh based on Breyer et al (2019).. Source: Beuttler et al (2019), alternative: Breyer et al (2019).
direct air capture	all	electricity	0.40 MWh	CO2 (g)	1.00 t	0.4 MWh based on Beuttler et al (2019) for Climeworks LT DAC, alternative value: 0.182 MWh based on Breyer et al (2019). Should already include electricity for water scrubbing and compression (high quality CO2 output).. Source: Beuttler et al (2019), alternative: Breyer et al (2019).
electrolysis	all	water	9.00 m3	hydrogen (g)	33.33 MWh(LHV)	Based on ideal conversion process of stochiometric composition.. Source: -
electrolysis	2050	electricity	44.44 MWh	hydrogen (g)	33.33 MWh(LHV)	nan. Source: nan

Table 8 (continued).

process	year	from	amount	to	amount	details and source
electrolysis	2040	electricity	46.62 MWh	hydrogen (g)	33.33 MWh(LHV)	nan. Source: nan
electrolysis	2030	electricity	49.02 MWh	hydrogen (g)	33.33 MWh(LHV)	Alkaline electrolysis AEC. May potentially increase to 70-80% in the future.. Source: DEA (2021): Technology Data for Renewable Fuels, pg. 97.
electrolysis	2020	electricity	50.13 MWh	hydrogen (g)	33.33 MWh(LHV)	nan. Source: nan
industrial heat pump medium temperature	all	electricity	1.00 MW	heat	3.00 MW(th)	based on DEA technology data catalogue for industrial process heat (302, pg. 48, 2020). Conservative for 20°C → 100°C .. Source: DEA
methanation	all	hydrogen (g)	17.81 MWh(LHV)	methane (g)	13.89 MWh(LHV)	Additional H2 required for methanation process (2x H2 amount compared to stochiometric conversion).. Source: Götz et al. (2016), fig. 11 .
methanation	all	CO2 (g)	2.75 t	methane (g)	13.89 MWh(LHV)	Based on ideal conversion process of stochiometric composition (1 t CH4 contains 750 kg of carbon).. Source: nan
methanolisation	all	electricity	1.50 MWh	methanol	5.54 MWh(LHV)	nan. Source: DECHEMA 2017: DECHEMA: Low carbon energy and feedstock for the European chemical industry , pg. 65.
methanolisation	all	CO2 (g)	1.37 t	methanol	5.54 MWh(LHV)	nan. Source: DECHEMA 2017: DECHEMA: Low carbon energy and feedstock for the European chemical industry , pg. 66.
methanolisation	all	hydrogen (g)	6.30 MWh(LHV)	methanol	5.54 MWh(LHV)	189 kg(H2) per t_MeOH, LHV for both assumed.. Source: DECHEMA 2017: DECHEMA: Low carbon energy and feedstock for the European chemical industry , pg. 64.

Table 8 (continued).

process	year	from	amount	to	amount	details and source
seawater desalination	all	electricity	0.00 MWh	water	1.00 m3	Desalination using SWRO. Assume medium salinity of 35 Practical Salinity Units (PSUs) = 35 kg/m3.. Source: Caldera et al 2016: Local cost of seawater RO desalination based on solar PV and windenergy: A global estimate. (https://doi.org/10.1016/j.desal.2016.02.004), Fig. 4.
