# Supplementary material for: Import options for chemical energy carriers from renewable sources to Germany
Source: PLoS One. 2023 Feb 9;18(2):e0262340. doi: 10.1371/journal.pone.0281380 (PMC9910710; doi:10.1371/journal.pone.0281380)
Supplement: S5 Table — (PDF) [file pone.0281380.s012.pdf]

S 11 Table   Shipping parameters

Table 9. Shipping parameters used. A machine readable version of these assumptions can be found in the Zenodo and GitHub repositories listed in the data availability section.

ship type	parameter	unit	value	details and source
CH4 (l) transport ship	(un-) loading losses	%/transfer	0.70	Approx. 450 t for a 130 000m^3 tanker based on https://citeseerx.ist.psu.edu/viewdoc/download?doi=10.1.1.470.6116&rep=rep1&type=pdf , pg. 59 f .
	(un-) loading time	h	48.00	Hurskainen 2019: https://cris.vtt.fi/en/publications/liquid-organic-hydrogen-carriers-lohc-concept-evaluation-and-tech , table 8.
	average speed	km/h	37.00	Hurskainen 2019: https://cris.vtt.fi/en/publications/liquid-organic-hydrogen-carriers-lohc-concept-evaluation-and-tech , table 8.
	boil-off	%/h	0.01	ca. 0.1% per day.Lowell et al. 2013, pg. 14.
	capacity	MWh_LHV	809 717.00	Based on CH4 LHV of 13.8888 MWh/t_CH4 and 58300 t capacity.Calculated.
	energy demand	MWh/km	0.57	Taken from source (average fuel demand / average cruising speed) without 50% efficiency.Calculated based on Hurskainen 2019: https://cris.vtt.fi/en/publications/liquid-organic-hydrogen-carriers-lohc-concept-evaluation-and-tech , table 8.
H2 (l) transport ship	(un-) loading losses	%/transfer	2.00	Runge et al 2020, pg. 8.
	(un-) loading time	h	48.00	Hurskainen 2019: https://cris.vtt.fi/en/publications/liquid-organic-hydrogen-carriers-lohc-concept-evaluation-and-tech , table 8.

Table 9 (continued).

ship type	parameter	unit	value	details and source
	average speed	km/h	30.00	Hurskainen 2019: https://cris.vtt.fi/en/publications/liquid-organic-hydrogen-carriers-lohc-concept-evaluation-and-tech , table 8.
	boil-off	%/h	0.01	lH2 tank losses (boil off) per day: 0.2%Runge et al 2020 pg. 8. and IEA (2019): The Future of Hydrogen, Assumptions Annex, pg. 7.
	capacity	MWh_LHV	378 666.00	Corresponds to 11360 t H2 (l) with LHV of 33.3333 MWh/t_H2Cihlar et al 2020 based on IEA 2019, Table 3-B
	energy demand	MWh/km	0.41	IEA (2019): The Future of Hydrogen, Annex Assumptions (Transmission)
LOHC transport ship	(un-) loading losses	%/transfer	0.00	Guesstimate, transfer of simple non-cryogenic fluid.
	(un-) loading time	h	48.00	Hurskainen 2019: https://cris.vtt.fi/en/publications/liquid-organic-hydrogen-carriers-lohc-concept-evaluation-and-tech , table 8.
	average speed	km/h	27.80	Hurskainen 2019: https://cris.vtt.fi/en/publications/liquid-organic-hydrogen-carriers-lohc-concept-evaluation-and-tech , table 8.
	boil-off	%/h	0.00	Guesstimate, simple non-cryogenic fluid in closed tanks.
	capacity	MWh_LHV	140 000.00	Assuming DBT as LOHC. Only ca. 90% rate of discharge of H18-DBT recommended, e.g. 1.87 MWh/t effective energy density (5.6 wt-% hydrogen, 33.3333 MWh/t_H2 LHV). 75000 t capacity for LOHC (H18-DBT form).Calculated, based on Runge et al 2020, pg. 7, https://papers.ssrn.com/abstract=3623514

Table 9 (continued).

ship type	parameter	unit	value	details and source
	energy demand	MWh/km	0.24	Taken from source (average fuel demand / average cruising speed) without 50% efficiency.Calculated based on Hurskainen 2019: https://cris.vtt.fi/en/publications/liquid-organic-hydrogen-carriers-lohc-concept-evaluation-and-tech , table 8.
MeOH transport ship	(un-) loading losses	%/transfer	0.00	Guesstimate, transfer of simple non-cryogenic fluid.
	(un-) loading time	h	48.00	Assume same as for LOHC.
	average speed	km/h	27.80	Assume same as for LOHC.
	boil-off	%/h	0.00	Guesstimate, simple non-cryogenic fluid in closed tanks.
	capacity	MWh_LHV	415 208.00	Based on MeOH LHV of 5.53611 MWh/t_MeOH and 75000t capacity.Calculated.
	energy demand	MWh/km	0.24	Assume same as for LOHC.
NH3 (l) transport ship	(un-) loading losses	%/transfer	0.00	Guesstimate, possibly negligible losses due to relatively close to ambient transport temperature.
	(un-) loading time	h	48.00	Assume same as for CH4 (l).
	average speed	km/h	37.00	Assume same as for CH4 (l).
	boil-off	%/h	0.00	Guesstimate, possibly negligible losses due to relatively close to ambient transport temperature.
	capacity	MWh_LHV	273 830.00	Based on NH3 LHV of 5.1666 MWh/t_NH3 and 53000 t capacity.Calculated.
	energy demand	MWh/km	0.57	Assume same as for CH4 (l).
FT fuel transport ship	(un-) loading losses	%/transfer	0.00	Guesstimate, transfer of simple non-cryogenic fluid.
	(un-) loading time	h	48.00	Assume same as for LOHC.
	average speed	km/h	27.80	Assume same as for LOHC.
	boil-off	%/h	0.00	Guesstimate, simple non-cryogenic fluid in closed tanks.

Table 9 (continued).

ship type	parameter	unit	value	details and source
	capacity	MWh_LHV	896 250.00	Based on FT fuel LHV of 11.95 MWh/t_FTfuel and 75000t capacity.Calculated.
	energy demand	MWh/km	0.24	Assume same as for LOHC.
