# Supplementary material for: Import options for chemical energy carriers from renewable sources to Germany
Source: PLoS One. 2023 Feb 9;18(2):e0262340. doi: 10.1371/journal.pone.0281380 (PMC9910710; doi:10.1371/journal.pone.0281380)
Supplement: Attachment — Submitted filename: response-to-reviewers.pdf [file pone.0281380.s013.pdf]

0 Response formatting and colour codes

We use the following formatting and colour coding for our response:

> *Comment:*
> Comments we received from the editor and reviewers are formatted like this.

> *Our response:*
> Our responses or actions we took following the comments we received are formatted like this.
>
> > 'Direct quotes from our manuscript are formatted like this.'

1 Academic Editor

> *Comment:*
> 1. Please ensure that your manuscript meets PLOS ONE's style requirements, including those for file naming. The PLOS ONE style templates can be found at
> https://journals.plos.org/plosone/s/file?id=wjVg/PLOSOne_formatting_sample_main_body.pdf
> and
> https://journals.plos.org/plosone/s/file?id=ba62/PLOSOne_formatting_sample_title_authors_affiliations.pdf

> *Our response:*
> We have updated our manuscript so that it should now be aligned with the style requirements.

> *Comment:*
> 2. We note that Figures 2 and 24 in your submission contain [map/satellite] images which may be copyrighted.
> [. . .]
> We require you to either (1) present written permission from the copyright holder to publish these figures specifically under the CC BY 4.0 license, or (2) remove the figures from your submission:
> [. . .]

Our response:

- The figure 2 is a self-created, derivative work based on a map taken from Wikimedia Commons (`https://commons.wikimedia.org/w/index.php?title=File:Blank_world_map_pusta_mapa_%C5%9Bwiata.png&oldid=505814435`). The map is available under CC0 1.0 license.
- The figures in (former) figure 24 in the supplementary material (now S 12 Figs) are of own creation using the Julia package 'GeoMakie' and thus does not require a written 3rd party consent.

Comment:
3. Please ensure that you refer to Figures 12, 13, 14, 15, 16, 17, 18, 19, 20, 21, 22, 23 in your text as, if accepted, production will need this reference to link the reader to the figure.

Our response:
We have created references to the respective supplementary information at the end of our manuscript as asked for in the PLOS one style templates mentioned above.

Comment:
4. Please review your reference list to ensure that it is complete and correct. If you have cited papers that have been retracted, please include the rationale for doing so in the manuscript text, or remove these references and replace them with relevant current references. Any changes to the reference list should be mentioned in the rebuttal letter that accompanies your revised manuscript. If you need to cite a retracted article, indicate the article's retracted status in the References list and also include a citation and full reference for the retraction notice.

Our response:

- We've reviewed and updated our references and the reference information.
- We've added the following references during the revisions sparked by the reviewer's comments: [1–21]
- We've removed the following references and substituted them by other references: [22–25]

2 Reviewer 1

General

Comment:
- [No action] Great work comparing more carriers than previous studies and with all the data available. My main comments are around the data and sources used which could impact the results and ranking between pathways. Other aspects such as presentation of the results, clarity of the discussion, conclusions supported by the analysis and alike need limited improvement

Our response:
Thank you! We thank the reviewer for their kind comments and the detailed review of our assumptions they have provided. These comments and suggestions have immeasurably improved the manuscript and we will acknowledge this in the paper. We have made extensive changes based on the reviewer's feedback: we have changed some of the data inputs and sources (see our responses below), recalculated all of the results and added a new graphic (Fig. 11) comparing our results to historic prices for fossil-based energy carriers.
We have acknowledged reviewers Herib Blanco and the anonymous reviewer in appreciation in the Acknowledgements.

2.1 Literature review

Comment:
- [1-5] (and many of the studies below) are missing

Our response:
Thank you for the suggestions, we have added them to our literature review along with some other literature which was published in the meantime.

2.2 Design of Energy Supply Chains

Comment:
- You might consider expanding the scope by one more (steel) and make it "commodities" instead of "chemical energy carriers". You can find the relevant data here [6] and then you can have all the comparison in one place

Our response:
We thank the reviewer for the suggestion, but we decided to restrict the scope in this paper to chemicals typically used as energy carriers. We will consider adding steel in future works as a commodity and potential energy carrier.

Comment:
- Page 4, line 104. Storage can make a big difference for liquid hydrogen since it is expensive to store it in large quantities or to deal with the boil-off. Making the comparison without storage will favour LH2. You can find some data for LH2 storage in [5, 6] for example

Our response:
We agree that storage costs can make a significant difference and are especially high for H2 (l). We have rephrased the sentences to make it more clear to readers that we do in fact include storage (incl. H2 (l) storage) on the exporter terminal side. Only storage on the importer side is beyond the scope of our study. On the importer side the storage volumes/durations highly depend on the downstream usage (i.e. required form of energy carrier, demand pattern) which we wanted to exclude from our study. We expect optimised conditioning of the imported energy carrier depending on specific needs to happen on the importer side. As your referenced [5] also shows, the cost for the import terminal can be higher for MCH or NH3 than for H2 (l) if conversion to H2 also has to be considered. We have rephrased the paragraph to read as follows:

> 'We exclude the transport within the exporting and importing country and storage in the importing country, i.e. excluded is
>
> 1. transport of electricity, feedstocks and chemical energy carriers between facilities and to the export terminal
> 2. short-term or long-term storage at the importer in Germany (DE)
> 3. distribution and end use of chemical energy carriers within DE
>
> We exclude these steps because the range of possible options, such as the time patterns of the demand, would create too many scenarios and detract from the generality of our analysis. Such scenarios are better suited for investigation in specific case studies.'

2.3 Investment optimization problem

Comment:
- Page 5, line 136. The limits for flexibility of the synthesis units (both ramping rate and minimum turndown) is not yet known and this will constrain the flexibility of the overall plant and determine the sizing of the storage. See [7, 8] for ammonia and [9] for FT

Our response:
We agree and are aware of this limitation. We have tried to choose reasonable short- to medium-term estimates on the must-run constraints and hope to have made this point sufficiently clear. The discussion of results also contains the following paragraph: Levers for decreasing overall costs lie in reduction of storage costs by either direct reduction of the investment costs or usage of different technologies, e.g. cavern storage instead of steel-tank storage for H_2. An indirect leverage is the flexibility of synthesis processes as the must-run processes drive the storage volumes in our results. Increasing flexibility of processes and enabling lower must-run capacities as well as hot-standby would decrease the required storage volumes. Simultaneously it would increase the cost share of the synthesis process, such that cost improvements of the said processes could have a higher impact in lowering total product costs.

Comment:
- Page 5, line 144. There are economies of scale that cannot be ignored. You can take some of the scaling exponents from Table 6.1 of [10] and use the standard 0.67 or 0.7 for the remaining technologies. If you take linear scaling, you both overestimate the costs (putting shipping at a disadvantage) and favour liquid hydrogen that requires a larger scale to become cost effective [10]

Our response:
Based on the other comments on capital cost assumptions we have revised our input assumptions to be considered for large scale facilities where economies of scale are already considered and further capacity expansion requires new facilities / infrastructure. We have revised the paragraph in an effort to highlight this more clearly.
From (old version)

> ‘Capital costs for all components scale linearly (scaling factor = 1) with their capacity representing a situation where capacity expansion requires new facilities rather than extending existing ones. Learning effects and economies of scale enter our model exogenously by choosing technology CAPEX and

> FOM assumptions for large facilities and an increase in world-wide capacity deployment where numbers from external analysis are available.'

To (new version)

> 'Capital costs for all components scale linearly with their capacity representing a situation where capacity expansion requires new facilities rather than extending existing ones. The used capital costs and Fixed Operation & Maintenance (FOM) already assume large scale facilities with respective economies of scale applied as well as exogenous learning rates for cost reductions between 2030 to 2050.'

Comment:
- Page 5, line 148. If costs scale linearly, how do you prevent that small facilities are built? Do you specify a minimum size below which the route is not possible (see e.g. [11] where they specify minimum trade flows set at 100 kt/yr for long-distance importers, 10 kt/yr for regional importers, and 1 kt/yr for internal EU trade, while in your case it would be a minimum size by technology or overall)

Our response:
We assume an import volume of 120 TWh/a for all scenarios (each ESC, exporter, year) which requires large facilities to be built. We do not use any additional constraints on facility sizes or capacities.

Comment:
- Page 5, line 149. Fixed efficiencies is not correct for hydrogen liquefaction, electrolysis, ammonia reconversion for which the values will probably change the most over time. Taking the low values (expected in the long term) already in 2022 would be too optimistic potentially underestimating the cost added by these steps

Our response:
We agree that fixed efficiencies do not properly reflect future prospective developments. We have tried to avoid using low (e.g. 2050) values and tried to assume near-future (e.g. 2030) numbers when possible. Technological development and improvements are difficult to properly quantify. Thus adding year-dependent efficiencies would likely result in an additional dimension of complexity and uncertainty. That being said, we have adapted time-dependent efficiencies for hydrogen electrolysis from which all ESCs would profit (and not only specific ESCs like with hydrogen liquefaction) and for which technological development is seen across all currently available technologies to be in the direction of improved efficiency. We have changed this sentence (old version)

‘Process efficiencies are assumed to not be affected from learning and are fixed for all years, see supplementary information ...’

to (new version)

‘Process efficiencies are assumed constant for all years (see **??**) due to the difficulty of making well-founded estimates for future technological developments and improvements. One exception is made for hydrogen electrolysis where efficiency is expected to increase across all available technology options [10, 26], an improvement which would affect all Energy Supply Chains (ESCs) presented here. Thus the electricity-to-hydrogen (Lower Heating Value (LHV)) efficiency for electrolysis is assumed to improve from 68 % in 2030 to 71.5 % in 2040 and 75 % in 2050.’

in order to reflect the change.

2.4 Choice of technologies and energy carriers

Comment:
- Page 6, line 174. Alkaline electrolysers have enough flexibility to provide grid balancing services, so for all practical purposes they are fast enough [12-14]

Our response:
Thank you for this comment. We have adjusted the wording to emphasise that this aspect has been something associated with AE in the past but that this property might not be an issue with today’s AEs and is in any case not of practical relevance for the systems investigated here. From (old version)

‘Compared to alternatives like proton exchange membrane (PEM) electrolysers, AEs electrolysers are less flexible in their operation and were for this property in the past considered less suitable for paired operations with RES. The advantages of AEs are that it is a well established technology. In comparison to PEMs, AEs have lower costs and do not require rare-earth-metals or platinum which may become bottlenecks for massive deployments in the future.’

to (new version)

‘Compared to alternatives like proton exchange membrane (PEM) electrolysers, alkaline electrolysiss (AEs) electrolysers were historically considered to be less suitable to provide grid services due to their slow start-up times

in the range of minutes [26]. For large scale operations in chemical energy carrier production the slow start-up time may be neglected because the electrolysis here does not need to provide grid services and the variability of Renewable Energy Source (RES) feed-in can be managed using for example battery storage. The main advantages of AEs are that it is a well established technology which has in comparison to PEMs lower costs and does not require rare-earth-metals or platinum which may become bottlenecks for massive deployments in the future.'

Comment:
- Page 6, line 199. Please add in parenthesis the energy efficiency of the LNG liquefaction step (e.g. "liquefaction of methane is energy intensive (96.5%)"
- because this energy penalty is much smaller [15]

Our response:
Thank you for the suggestion. We have added the energy demand for liquefaction for H2 (l) and CH4 (l): From (old version)

> 'The physical properties of hydrogen make it difficult to transport as gas (low energy density) or as cryogenic liquid (low temperature and high energy demand for liquefaction). [...] Similar to hydrogen liquefaction of methane is energy intensive and handling of the cryogenic LNG rather complex.'

to (new version)

> 'The physical properties of hydrogen make it difficult to transport as a gas due to its low energy density and as a cryogenic liquid due to the very low temperature required and high energy demand for liquefaction ($0.203\,\mathrm{MWh/MWh_{LHV}}$). [...] Liquefaction of methane requires less energy ($0.036\,\mathrm{MWh/MWh_{LHV}}$) than liquefaction of hydrogen and handling of cryogenic liquefied natural gas (LNG) is less complex and better established compared to liquefied hydrogen.'

Comment:
- Page 7, line 200. Please mention that already today 10% of the global ammonia production (of a total of 183 Mt) is already traded today [10] and this is an advantage that not all the pathways have

Our response:
Thank you for the suggestion. We have extended the sentence from (old version)

> 'In addition industry has long-standing experience of transporting and handling ammonia in its various forms.'

to (new version)

> 'In addition industry has long-standing experience of transporting and handling ammonia in its various forms, with around 10 % of the global 183 Mt annual ammonia production being traded [11].'

Comment:
- Page 7, line 212. (1) Ref for this statement is missing "high energy requirements (> 25 % of ammonia's LHV)"; (2) There are a few examples of large-scale application of ammonia cracking (in heavy-water production) [10] and the design would be very similar to SMR furnaces [16] which decreases significantly the risk

Our response:
Thank you for pointing out the already existing use case for ammonia crackers in D2O production. We have changed the previous formulation from (old version)

> 'The chemical bonds of ammonia make direct energetic use challenging. The dehydrogenation of NH3 to obtain H2 through thermal decomposition is not well established yet and has high energy requirements (> 25 % of ammonia's Lower Heating Value (LHV)).'

to (new version)

> 'Direct energetic use of ammonia is possible but poses a range of challenges including suppression of NO_x emissions [12]. Alternatively ammonia may be used as hydrogen carrier where the dehydrogenation of NH_3 happens through thermal decomposition. The dehydrogenation process has high energy requirements (> 25 % of LHV [13]) and the technology is not yet fully commercially mature. Large specialised ammonia crackers are already in operation for the production of heavy water [11].'

Comment:
- Page 7, line 219. Please add a few sentences on the current scale of methanol reforming and cost ratio with methanol synthesis

Our response:
We have changed the previous sentences (old version)

> 'We focus on methanol as energy carrier it can also considered a LOHC with

> its dehydrogenated form being CO2 [5].'

and adapted the following sentences (new version)

> 'With an annual production volume of ca. 100 Mt [14] methanol synthesis is a well-established process with industry experience in handling, storage and transport. Methanol is used either as a industrial feedstock, for power and heat generation, as a mobility fuel [15,16] and also be considered a member the Liquid Organic Hydrogen Carrier (LOHC) family with the dehydrogenated form being CO_2 [27]. Methanol-to-hydrogen cracking (steam methanol reforming) is mature on small industrial scales [17] and practical where methanol logistics are easier than logistics for alternative hydrogen feedstocks like e.g. natural gas.'

Comment:
- Page 7, line 230. Clarify in this section what the product split is (e.g. to jet fuel/kerosene/diesel). You mention the average LHV, but that is for energy balances purposes. The other relevant aspect is that the products satisfy different end uses and the jet fuel share is more relevant (assuming diesel consumption will decrease significantly once ZEV are adopted)

Our response:
As we write in the short description for Fischer-Tropsch fuels, we only consider the liquid fractions after FT synthesis, which we estimate to constitute 80% of the product. The liquid fractions are assumed to have the same specific energy of 11.95 MWh/t and same energy density. More details on the FT synthesis and fuel split are provided in supplementaries, table 8 for "Fischer-Tropsch synthesis":

> 'Product composition can be assumed to be 60% jet fuels/20% kerosene/20%LPG & fuel gases after upgrading, but we discard the LPG&fuel gas by-product in this assumption – we're focusing on the liquid products. Liquid products both have LHVs of 11.95 MWh/t.. Source: DEA technology data for Renewable fuels (2018): Hydrogen to Power.'

Comment:
- Page 7, line 234. (1) For LOHC, one challenge is the small scale today compared to what is needed for global trade. Mention briefly what the assumption is for the costs (same as small scale since costs scale linearly?); (2) Another big limitation is the cost of the carrier itself. Mention in one sentence what the assumption is

Our response:
We agree with the comment and have made the following changes: We have checked and accordingly updated our cost assumptions for LOHC dehydrogenation to reflect a large dehydrogenation plant (1 GW_{H2} output capacity). Earlier it was indeed reflecting small scale dehydrogenation with higher specific cost. We have added the cost for the LOHC chemical to the text:

> 'The cost for the LOHC chemical dibenzyltoluene (DBT) is assumed to be 2264 EUR_{2015}/t.'

The odd value 2265 EUR_{2015}/t value is due to the inflation correction of 2265 EUR_{2020}/t to EUR_{2015}.

Comment:
- For this section, I realize many of the answers are in Table 7 (and in the Zenodo repository)

Our response:
We're happy to hear those resources already answer some questions.

Comment:
- Page 8, line 279. (1) Revise 94.25% based on [9]; (2) Methanol should have a similar flexibility to HB and FT

Our response:
We considered [9] and our own references again and decided not to make any changes. Reasoning: We assume the same must-run capacity for Methanol and Fischer-Tropsch synthesis of 94.25% which we deem consistent with [9] ("The Today case assumes that the fuel synthesis must remain constantly at 100% production." from [9] supplementary information) where the remaining 5.75% represented planned and unplanned plant offline hours. It is also consistent with the DEA renewables fuels report (`https://ens.dk/sites/ens.dk/files/Analyser/technology_data_for_renewable_fuels.pdf`) which states "the plant should be able to operate at an average annual rate of 95%" (pg. 210). Chen and Yang (2021,) note that "Currently, there is no experimental or theoretical data available regarding the load range of a methanol synthesis reactor." when they looked into the benefits of flexibility in the methanol synthesis process using CO2 + H2. Based on this we do not have enough reasons to comfortable assume lower flexibility for now but we can't exclude that the current (inflexible) operation is due to economic reasons rather than to inherent limitations of the process. The higher flexibility for Ammonia synthesis (HB) of 30% must-run capacity is kept based on the DEA Renewable Fuels

report which states a regular operational range of 70%-100% today and 30-100% to be a feasible operational range without major changes to the plant designs.

2.5 Countries investigated

Comment:
- Table 2. Can you eliminate some of the combinations (e.g. HVDC from Argentina and Australia and shipping from Denmark. The former countries are just too far to realistically consider HVDC and shipping from less than 1000 km while having a land connection possible is not realistic either)?

Our response:
We prefer to keep all countries in the spirit of a scientific thought experiment that doesn't exclude options a priori. Electricity supply, demand and supply curves

Comment:
- Page 11, line 345. 1.45 MW/km2 seems extremely low. If I check your ref 25, it seems the 1.45 MW/km2 comes from Section 2.5 that has an arbitrary 1% (145 MW/km2 * 0.01), see e.g. [25] (but there are many others)

Our response:
We understand the concerns: For a technical potential the 1.45 MW/km2 would indeed be very low. The 1.45 MW/km2 are meant to represent a "deployable" potential and serves as a grand average across a multitude of available areas which are available after GIS analysis. Only a small percentage of this area will be available and used for PV installation (due to land restrictions, land competition and social-acceptability criteria which are not considered in the GIS analysis). We therefore believe our assumptions to be realistic.

Comment:
- Table 3. The cost reduction profile does not seem to be realistic. Today, global average CAPEX for solar PV is about USD 890/kW [26] and you are assuming it decreases by more than half towards 2030, only to decrease by another 12.5% towards 2040 (vs 2030). I would suggest a higher value for 2030 (and potentially also 2040), while the 2050 value seems reasonable [27] (same comment applies to onshore and offshore wind)

Our response:
The value for utility scale solar PV seems indeed low at a first glance and strongly depends on the expected learning rate in the future as well as capacity expansion.
We rely on data from the DEA and believe this has high value because it is a homogeneous data basis with a uniform methodology for data collection and processing. Given that strong learning has lead to significant decrease in solar utility PV cost (e.g. 2010-2020: 46-85% reported here: https://www.irena.org/Statistics/View-Data-by-Topic/Costs/Global-Trends and 53%-14% expected here: https://atb.nrel.gov/electricity/2022/utility-scale_pv) we consider this number consistent and realistic assumption.
To quantify a potential expansion and learning pathway: At learning rates between 23%-40% (cf. Fig 2B, doi:10.1016/j.joule.2021.03.005) this corresponds to world-wide cumulative capacities between 9.85 to 3.25 (= 2^3.32 to 2^1.7) times of today's installed capacity by 2030 which are realistic capacity expansions by 2030.

Comment:
- 10% WACC everywhere does not seem to be the best approach. At least you could use current WACC [28,29]

Our response:
We agree that a homogeneous WACC assumption of 10% does not represent the current values of WACC for all countries. We decided against using different WACC values for different countries for multiple reasons: Comparability: By using the same WACC assumptions for all regions the results are not influenced by another parameter dimension and thus easier to compare. Lower and upper estimates: We provide a lower (5% WACC) and upper (10% WACC) cost estimation for all ESCs to show the range of potential values. This range (5-10%) should capture future WACCs for most regions, as WACC is not only determined by the renewable technology, but also by the other technologies used. This approach allows us also to present the reader with two values to choose from, depending on which WACC assumption is deemed realistic for the specific circumstances. Unknown influence of state actors: Given that we here demonstrate costs for large scale energy supply chains to Germany which can be linked to national interests and thus potentially also to state-level guarantees from countries like Germany, we argue that WACC might actually be lower for projects of this calibre despite being realised in regions with (today) high WACC values. For example, off-taker guarantees from Germany for the H2Global initiative are leading to lower WACC than the local country WACC. Further influences on WACC: Taking current WACC for the countries does not account for learning effects in financing as projects scale up in the country. Since these effects are complex to capture, we prefer a simple approach with two reference WACCs of 5% and 10%.

2.6 Results

Comment:
- Figure 4. LH2 with a similar cost than NH3 by 2030 does not seem right. This might be rooted in the several assumptions that favour LH2 (see previous comments, in particular LH2 storage needs in the terminal). Those should be corrected to give a more complete view

Our response:
We have adjusted our model and input parameters as described above and updated the figure with the new results accordingly. Quantitatively the cost for H2 (l) shipping and NH3 (l) shipping did decrease. Qualitatively the cost of the two ESCs is still comparable after adapting the changes.

Comment:
- This is an outcome that should be highlighted more (e.g. conclusions or even abstract) " The exporting country preference order does not change much between the ESCs"

Our response:
We have adapted the following sentence in the conclusions:

> 'While better financing conditions and fast-track technology development lead to lower costs for imported energy and hydrogen, they do not lead to changes in in the order of preference regarding technology and exporter if they affect all exporters and ESC similarly.'

Comment:
- Figure 5. The almost constant cost decrease towards 2050 for all ESCs is difficult to believe. For instance, one extreme is LH2 where costs for liquefaction, storage, and ships still has to decrease significantly since it is all small scale or pilots today and most of those economies of scale will not be achieved by 2030, so I would expect a larger cost reduction for 2050 (vs 2030). In contrast, for FT, the only step that is missing is electrolysis, FT is commercial and it will be difficult to justify a 50% cost reduction for the synthesis and cost for ships will not decrease as much (as e.g. LH2 ships) over time (other than the use of larger ships which are already the case today)

Our response:
With the new results based on the updated input parameters the decrease in cost is less pronounced for e.g. FTF for AR from 180 (2030) to 140 (2050) EUR/MWh compared to previously 220 (2030) to 150 (2050) EUR/MWh. The cost decrease for FTF is driven by by the decrease in costs for electrolysis as well as for RES and DAC (cf. cost composition figures in text and supplementary materials). With the new results we can also observe a stronger cost decrease for e.g. H2 (l) by ship for AR from 100 (2030) to 75 (2050) EUR/MWh compared to previously 120 (2030) to 110 (2050) EUR/MWh.

Comment:
- Figure 5. I would expect the costs to be much lower since FT efficiency is higher than methanation, the CAPEX is comparable and the transport is much cheaper (so it should be cheaper than CH4 ship). The only downside is the higher CO2 use but that should not be the reason for a 50

Our response:
With the new results the cost difference between FTF and CH4 (l) shipping is less pronounced but still high. E.g. for AR CH4 (l) is 100 (2030) to 75 (2050) EUR/MWh while FTF is 185 (2030) to 135 (2050) EUR/MWh. The higher CO2 demand for FTF (3.9 t / MWh) compared to CH4 (2.75 t/MWh) increases the required investment for DAC and increases the input electricity demand and thus necessary RES investments (cf. Fig 6.). The CO2 demand for FTF is 20% above the DEA literature value as we disregard the gaseous fractions yielded by the FTF synthesis.

Comment:
- Discussion should include (1) How come LH2 is cheaper than CH4 pipeline (i.e. pipeline does not have boil-off losses and it can handle larger flows of energy. It has the losses of the methanation step, but this is only about 20% compared to 30% for liquefaction, so conversion losses are lower and conversion and transport costs are also lower); (2) Why CH4 ship is cheaper than CH4 pipeline (there are losses for liquefaction and the ship has the boil-off losses)

Our response:
We have added the following sentence in the discussion on “Cost compositions and drivers”:

> ‘This energy demand for transport requires additional capacities for CH_4 synthesis, H_2 electrolysis and CO_2 capture as well as RES capacities, thus making pipeline transport less attractive across medium and long distances

> compared to H_2 and CH_4 shipping.'

And in the "Limitations" section:

> 'Also some technologies might realise technological maturity faster or slower than others leading to earlier or later cost decreases in some ESC thus influencing the time-horizon of the results presented.'

The advantage of H2 (l) shipping compared to CH4 (g) pipeline is caused by a number of factors:

- H2 (l) / CH4 (l) liquefaction can use electricity as input in our model while energy demand of CH4 (g) pipelines has to be met using the CH4 (g) inside the pipelines, thus requiring more CH4 synthesis including feedstock production
- H2 (l) liquefaction is assumed to be similarly efficiency 20% as the CH4 synthesis
- H2 (l) and CH4 (l) shipping boil-off losses can be fully used for ship propulsion, thus leading to no additional efficiency losses along an ESC

Further limitations:

- H2 (l) shipping might not realise the (optimistic) cost decreases which are assumed here while CH4 (g) pipeline is already a mature technology, thus cost decreases only affect the feedstock sourcing and synthesis

For short distance transport CH4 (g) pipelines are still the better option compared to CH4 (l) shipping. For medium to long-distance transport (ca. 4000 km, e.g. Egypt) CH4 (l) shipping becomes more economic. This is consistent with literature values, e.g. Fig. 2.22 from [28] where the switch between economic preference of LNG vs. pipeline is between 3000 and 7000 km.

Comment:
- Page 17, line 512. From Figure 1, it was not clear that battery storage was included (I guess for increasing the effective capacity factor of PV?). Modify accordingly to make it clear

Our response:
Buffer storages are available for every chemical and energy carrier, including battery storage. The idea is to provide the model options for optimisation, e.g. to increase the load factor of transmission infrastructure by expanding buffer storage up- or downstream of the transmission infrastructure.
We have changed the figure description to mention the inclusion of buffer storages:

> 'Schematic representation of ESCs considered in this study.

ESCs cover electricity generation from RES, intermediary buffer storage for electricity and chemicals, conversion to chemical energy carriers, conditioning and transport from exporter to importer. Each ESCs delivers one out of five chemical energy carriers. Detailed representations of each ESC and all involved technologies are included in supplementary information...'

Comment:
- Not clear why the renewable electricity cost contribution would be almost double for FT vs CH4 (see comment above) considering the pathway efficiency is similar. A higher flexibility for FT (or more comparable to methanation) should also make hydrogen storage cost for FT smaller

Our response:
There are multiple effects at work here: The efficiency for Fischer-Tropsch synthesis is worse than for methanation, partially because we disregard the gaseous fraction resulting from the FT synthesis. This leads to an overall more energy intensive ESC for FTF vs. CH4, e.g. for AR the ESF for FTF is 3 while it is 2.2 for CH4 (l) by ship (see Fig. 11 left side) The different flexibilities of the two synthesis processes leads to different mixes of RES capacities installed and used for producing electricity. E.g. for AR for the FTF ESC more PV is installed and used to produce electricity than for the CH4 (l) case. In the second case more wind is favoured as a more cost-effective source for electricity. We see different RES mixes for most exporters, more notably for AU and DK. The amount of curtailed electricity for the FTF ESC is also higher for the CH4 (l) ESC showcasing that curtailment is preferred to battery storage deployment but also further driving the RES cost share for the FTF ESC (see Fig. 11 right side). E.g. for AR 16% (FTF) vs. 2% (CH4 (l)). Based on this we agree that in principle a higher flexibility would have a significant cost effect. To express this aspect towards the reader we have added the following paragraph:

> 'Inflexibilities in the synthesis processes do not directly become apparent from the cost composition figure but show up indirectly by the need for increased buffer storage capacities and overcapacities of components upstream in the ESCs, e.g. RES. A good example for this are the Fischer-Tropsch fuel (FT fuel) and CH_4 (l) ESCs where the more inflexible FT fuel synthesis leads to higher RES investments per MWh with higher curtailment and a different mix of RES capacities (see **??**).'

2.7 Hydrogen import costs for 2030 to 2050

Comment:

- Please make more explicit and clearer earlier in the text that you are covering energy transport (with different carriers as end use) and hydrogen transport (all reconverted to hydrogen). Up to this point, I still had the question if for instance FT was being used as hydrogen carrier (which would make little sense) but I realize now the figures above are just comparing different carriers delivered (i.e. not directly comparable)

Our response:

We have adapted a new version of Fig. 1 trying to visualise this aspect more clearly. We have also adapted the following paragraph in the introduction of the results section:

> 'Results are compared with a focus on their Levelised Cost of Energy (LCoE) and Levelised Cost of Hydrogen (LCoH). The LCoE represent the costs for delivering 1 MWh of energy in the form of the main energy carrier of the respective ESC, i.e. H_2 (g), CH_4 (g), NH_3 (g), methanol and FT fuel. For the LCoH costs are compared for delivering 1 MWh of H_2 (g) as commodity to the importer.'

And the following paragraph in the beginning of the LCoH cost comparison section:

> 'In addition to comparing the costs of energy delivered, we can also compare the ESCs based on their import costs for delivering hydrogen. For this we calculate the levelised cost of delivering 1 MWh H_2 (g) using adapted versions of the previously discussed ESCs. Comparing the cost of hydrogen rather than the cost of energy is useful for applications which either require pure hydrogen like hydrogen fuel cells or processes requiring hydrogen as an industrial feedstock.
>
> The ESCs used are identical where the ESCs already delivered H_2 (g). The CH_4, NH_3 and MeOH ESCs are extended by cracking processes for converting the energy carrier to H_2 (g) with their respective energy demand and investment costs. The additional cracking processes are steam methane reforming (SMR), methanol steam reforming (MSR) and ammonia cracking. Additional water demand of the cracking processes is neglected. The extra process steps and their location in the ESCs are shown in **??**. We exclude cracking of FT fuel as methanol can be considered an equivalent choice for the purpose of being a liquid, carbon-based hydrogen carrier under ambient conditions but with easier synthesis. FT fuels are better suited as drop-in fuel replacements.'

Comment:
- Page 18, line 543. This point should make it clearly to the conclusions "by 2050 as most exporting countries except for AR, AU and DE show nearly identical LCoHs", so where it comes from does not really make a difference

Our response:
We have changed the sentence slightly to:

> 'By 2050 as most exporting countries show similar LCoH the question of exporter makes no big difference from a techno-economic point of view as there does not seem to be an inherent advantage to a specific exporter.'

And in the conclusions:

> 'In fact no single exporter or ESC shows a unique techno-economic advantage over the others, leading to the conclusion that preference for one energy carrier, ESC and exporter should not be given solely based on small differences in LCoE and LCoH. '

And in the abstract:

> 'No single exporting country or energy carrier has a unique cost advantage, since for each energy carrier and country there are cost-competitive alternatives. This allows exporter and infrastructure decisions to be made based on other criteria than energy and cost.'

2.8 Lower WACC

Comment:
- More important than testing lower WACC across all countries is considering the WACC differentials across countries. While there are no good values for 2050, the most sensible assumption would be to assume they stay similar to the current values. This would be a better sensitivity (or base) scenario since the only aspect you are measuring by doing it all 5% instead of 10% is how much cheaper it becomes which was already evident from Figure 8. I see this is in the Limitations section but I am not sure if it is good enough

Our response:
Also see our response above regarding 10% WACC vs. realistic WACC. We agree that different WACC assumptions would yield a valuable comparison. While we would like to investigate a more fitting base scenario we are not aware of a publicly accessible

up-to-date database of relevant WACC values. The benefit of homogeneous WACC assumptions as in our current study is that it allows for a better comparison of different ESCs with a multitude of carriers analysed under identical system boundaries and with the same methodology for input.
An additional factor influencing WACC is if the off-taker (e.g. Germany) guarantees a strike price for the ESC, then the off taker will have a stronger role determining the WACC than the local country's credit rating.
A lot of work is currently being produced on analysis based on region-specific WACC assumptions which has yet to converge towards results which we feel comfortable to base a study upon. We hope to use more adequate WACC estimates in the future and consider this a potential extension to our study left to future research. We have adapted the following sentence in the conclusions to address this:

> 'To overcome the limitations of this study and increase insight into energy imports, future research should focus on differentiating Weighted Average Cost of Capital (WACC) based on exporter and technologies, include spatially resolved RES and collection infrastructure, include additional relevant energy carriers and ESC designs like CO_2-cycling and import of secondary, energy-intense products like refined-iron or other valuable hydrocarbons and investigate sensitivities of results also to technical aspects like process flexibilities and synthesis must-run conditions.'

Comment:
- Page 21, line 601. Can you somehow include the comparison with current commodity prices in one of the figures above (e.g. an additional dot with a different marker or a shaded area for each commodity)? This will make it easier for the reader that might browse through the figures (instead of reading the entire text)

Our response:
Instead of showing the cost in one of the figures, we have included a new figure (Fig. 11) showing the past 3 years of commodity prices compared to average and median costs from our scenarios for each the 5 % p.a. and 10 % p.a. WACC case

Comment:
- Page 21, line 618. Make a figure with the CO2 costs

Our response:
Thank you for the suggestion. As the CO2 externality cost is not a focus of this paper we have decided to not include a figure with the CO2 costs. However, we have discussed the CO2 abatement costs in the text accompanying the new figure on fossil costs (Fig.

11).

2.9 Conclusions

Comment:
- Page 23, line 731. This is the part that is not fully self-explanatory, when you mention/use LCoE, it is not 100

Our response:
We have rephrased the sentence to make this more clear:

> 'For the nine different ESCs we minimised greenfield investment costs to determine the Levelised Cost of Energy (LCoE) per MWh_{th} of the specific chemical energy carriers as well as Levelised Cost of Hydrogen (LCoH) per kg_{H2} after dehydrogenation of the energy carriers.'

Comment:
- Make sure the comments above where I mention that those ideas should make it to the conclusions are incorporated

Our response:
This has been addressed.

2.10 Table 7

We have made a significant number of changes to the table. Among others we included the description and comments for the technologies which were already included in the data repository but which we missed to include into Table 7 in the paper.

Comment:
- Pipeline cost should be a function of capacity. It is not the same a small pipeline than a large one. Gas pipelines are cheaper than hydrogen [17-19]

Our response:
Certainly. As with all other cost assumptions in our study we are using a value deemed realistic for large scale energy exports/imports, specifically 120 TWh / a in our study. In this case the CH4 (g) pipeline CAPEX is estimated based on https://en.wikipedia.org/wiki/Arab_Gas_Pipeline for a large diameter pipeline including mid-way compressor (booster) stations.
For H2 (g) pipelines we already assume higher CAPEX than for CH4 (g) pipelines, namely 3x higher CAPEX based on the EHB (June 2021) report (https://gasforclimate2050.eu/wp-content/uploads/2021/06/EHB_Analysing-the-future-demand-supply-and-transport-of-hydrogen_June-2021.pdf) for H2 (g) pipelines incl. mid-way compressor (booster) stations.

Comment:
- Why not considering SMR with CCS? It is a relatively small cost penalty (USD 70-100/tCO2) for negative emissions (since that CO2 originally comes from DAC and I am assuming this is for SNG reconversion to hydrogen at the importing terminal)

Our response:
Comparing multiple dehydrogenation technology paths for the same energy carrier (methane) is outside the scope of our study. We also believe that combining green methane with CCS technology could be a potentially valuable and integrated way for realizing negative carbon emissions (given that methane leakage are addressed and CO2 capture rates are high). Small outlook: We are already working on a study where we consider imported green methane (based on CO2 from DAC and H2 from electricity generated with RES) in a sector coupled European Energy System model where this technology option may be endogenously chosen by the model to generate negative emissions for the energy system.

Comment:
- Depending on how much influence the CO2 liquefaction has, you might want to use some more pessimistic assumptions, [20] has almost double the cost and energy consumption

Our response:
Unfortunately we could not find the mentioned cost and energy consumption in the mentioned source. Too low energy demand assumptions would indeed be a problem. We see our energy demand assumptions being aligned to other reports, e.g. by [29, pg. 26]. Differences in CAPEX would not have a big impact as the CO2 storage cost dwarf the cost for liquefaction and evaporation. As for the CAPEX assumptions for CO2 storage made: They can be argued to be conservative as the source they are taken from [30] who

also cities values of 799 EUR/t_{CO_2} and 1859 EUR/t_{CO_2}. We used 2430 EUR/t_{CO_2} from Lauri et al 2014 which is a bit higher but should be in the correct order of magnitude. In general CO2 liquefaction/evaporation and storage has only minor contribution to the overall cost in all scenarios (<= 2% of LCoE in all scenarios).

Comment:
- The CAPEX for dehydrogenation in [Runge 2020] is too high because it is meant to be for HRS (i.e. small scale), it is more in the USD 100-250/kW range [10]

Our response:
Thank you for pointing out this important issue. We've adapted new values for LOHC hydrogenation and dehydrogenation based on [31], Table 9:

- Hydrogenation: 51 260 EUR_{2015}/MW (from 46 471 EUR_{2035}/MW)
- Dehydrogenation: 50 728 EUR_{2015}/MW (from 759 908 EUR_{2035}/MW)

Reuß et al. is the underlying source for most of the values referenced by IRENA (2022) for DBT dehydrogenation. We're assuming a 2 GW hydrogenation and 1 GW dehydrogenation plant with a scaling factor of 0.6 applied to 40 MEUR (30 MEUR) for hydrogenation (dehydrogenation) at a base plant capacity of 300 t/d.

Comment:
- HVDC. Please include complete reference

Our response:
Fixed, thank you.

Comment:
- HB. The CAPEX seems to be highly overestimated, please double check the value and see [7,8,10,20]

Our response:
The value is not overstated, but reported in the table of per MW electricity input, where the process consumes ca. 0.25 MWh electricity per 1 MWh_{NH3} output (i.e. ca. 1.352 MEUR/MW_{NH3} output) . This information was not obvious from the table for which we apologise (the same applied to the air separation unit). We've changed the table to make the unit more clearer.
For consistency we further decided to adapt the value from the newer DEA technology

data release [32] for Haber-Bosch and the air separation unit.
Previous values:

- Haber-Bosch:
  - 5 310 108 EUR/MW (2030), 4 % FOM, 20 years lifetime
  - 5 310 108 EUR/MW (2040), 4 % FOM, 20 years lifetime
  - 5 310 108 EUR/MW (2050), 4 % FOM, 20 years lifetime
- ASU:
  - 10 942 205 EUR/MW (2030), 4 % FOM, 20 years lifetime
  - 10 942 205 EUR/MW (2040), 4 % FOM, 20 years lifetime
  - 10 942 205 EUR/MW (2050), 4 % FOM, 20 years lifetime

New values:

- HB:
  - 1297.43 EUR/kW (2030), 3 % FOM, 30 years lifetime
  - 1061.17 EUR/kW (2030), 3 % FOM, 30 years lifetime
  - 813.55 EUR/kW (2030), 3 % FOM, 30 years lifetime
- ASU:
  - 729 306.18 EUR/(t_{N2} h), 3 % FOM, 30 years lifetime
  - 596 501.02 EUR/(t_{N2} h), 3 % FOM, 30 years lifetime
  - 457 307.78 EUR/(t_{N2} h), 3 % FOM, 30 years lifetime

Comment:
- Hydrogen regasification. The CAPEX seems to be too low, see e.g. [4, 22]

Our response:
Based on IRENA [8] and DNV GL [33] report we have adapted lower liquefaction cost (ca. 0.7x and 0.4x lower) and higher regasification cost (38x and 15x higher) as well as assumption for short-term (2030) as well as long-term (2050) development. The lower liquefaction cost are mainly warranted due to the lack of earlier scaling in the facility investment cost (economies of scale). We note that the low evaporation cost were correctly adapted from [31] which in turn is based on the Nextant 2008 report also reported by in [8] in Figure 3.15 where the Nextant report is also an outlier with very low investment cost. Given that the Nextant report is close to 15 years old we agree that newer numbers as reported by e.g. DNV GL / IRENA / Guidehouse in the EHB are more appropriate to be used.

Comment:
- Hydrogen storage tank. Specify pressure

Our response:
Type 1 storage tanks at <= 200 bar.

Comment:
- ASU. Since the conversion seems to have gone wrong for HB (from [Morgan 2013]), please double check the CAPEX because it seems too high (see [7,8,10,20])

Our response:
(Also see our comment above regarding HB) We've adapted the values from DEA for ASU for this assumption for consistency. While the old value was meant for a ca 1.070 MEUR/(t_{N2} h) facility, in combination with the high electricity demand for ASU (0.4 MWh_{el}/t_{N2}) it resulted in unintentionally high cost of 2.477 MEUR/(t_{N2} h). We have changed this number to an output-based number and are now using consistently the values from DEA of 0.729 MEUR/(t_{N2} h) (2030) and 0.457 MEUR/(t_{N2} h) (2050) for ASU and HB. Furthermore we have increased the ASU electric efficiency from 0.4 MWh_{el}/t_{N2} to 0.25 MWh_{el}/t_{N2} for consistency with the DEA report.

Comment:
- Methane fill compressor station. Why would this be needed? (the name seems to indicate compression for CNG cars or similar. If you mean a standard compression station for pipeline transport, then delete the "fill")

Our response:
The purpose of the fill compressor station for methane and hydrogen pipeline transport is to raise the initial pressure of the gas to pipeline pressure levels. Compared to booster stations along a pipeline which compensate for pressure losses along the transport route, the fill compressor stations have a special task as an entry point to the pipeline. We follow here the nomenclature used in the DEA report [34, pg. 122]

Comment:
- Hydrogen pipeline. Specify the diameter assumption for the CAPEX

Our response:
See updated table 7.

Comment:
- Methane liquefaction. It seems something went wrong for the CAPEX (0 EUR/kW for 2040 and 2050). I would expect somewhere in the 400-600 EUR/kW range [23]

Our response:
We found and fixed the error for CH4 liquefaction and evaporation. The new CAPEX are 190.43 EUR/kW (liquefaction) and 71.86 EUR/kW (regasification).

Comment:
- FT synthesis. (1) Unclear why the learning would be about 20% cost reduction for methanation and about 50% for FT (I would argue it should be similar to methanation since FT is already a commercial technology and there are limited improvements to reach 50% cost reduction); (2) EUR 1600/kW seems too high [24]

Our response:
We have revisited the original sources of these numbers. The numbers from these sources were considering a scale-up over the next years leading to relatively high 2030 cost and lower 2050 cost for methanol and FTF, but not for methanation (which was already considered in the costs to be scaled up). We decided to change to numbers based on this Agora report [35] for the cost for methanol, Fischer-Tropsch and methane synthesis which address both points. New cost assumptions are 2030/2040/2050 for methanation 628/554/480 EUR/kW$_{CH4}$ and 650/565/480 EUR$_{k}$ W$_{liquid}$ for methanolisation and Fischer-Tropsch (same for both technologies). All three technologies thus now see a similar decrease in cost.

Comment:
- Ammonia cracker. EUR 1400/kW is too high, see Figure 2.13 of [10]. You might as well consider that in the medium term (2030) relatively small crackers are used and the full economies of scale are reaped by 2040 and beyond

Our response:
The originally used number was for a small plant (200 t$_{NH3}$/d) at low TRL; unfortunately this was only noted in the additional description missing from Table 7 in the paper. After comparison with the IRENA (2022) report we decided to adapt new numbers based on Ishimoto et al (2020), namely 1062 EUR/kW$_{H2}$ (2030) and 527 EUR/kW$_{H2}$ (2050) for

200 t_{NH3}/d and 2500 t_{NH3}/d facilities respectively. The 2040 value used is interpolated based on these two values. For consistency we also adapted other assumptions from Ishimoto et al (2020): lifetime of 25 years (instead of 30 years prior) and FOM of 4.3% (instead of 3% prior).

Comment:
- Hydrogen liquefaction. Benchmark CAPEX with [10] and there should probably be a cost decrease over time

Our response:
Agreed. Value has been adjusted. See comment regarding Hydrogen evaporation (regasification) earlier.

2.11 Table 8

Comment:
- FT-CO2. Can you change the basis from 1t of fuel to 0.56 MWh of fuel (which the rows below seem to use and this would allow for a direct comparison)

Our response:
Adapted.

2.12 References from Reviewer 1

[1] https://www.ewi.uni-koeln.de/en/publications/estimating-long-term-global-supply-costs-for-low-carbon-hydrogen/

[2] https://www.globh2e.org.au/_files/ugd/8d2898_2778fbb92232442ba17942dc47b5f845.pdf

[3] https://www.sciencedirect.com/science/article/abs/pii/S0360319921016815

[4] https://doi.org/10.1016/j.ijhydene.2020.09.017

[5] https://repository.tudelft.nl/islandora/object/uuid%3Ad2429b05-1881-4e42-9bb3-ed604bc15255

[6] https://doi.org/10.1016/j.enconman.2022.115268

[7] DOI: 10.1039/d0ee01707h

[8] https://doi.org/10.1016/j.ijhydene.2019.11.028

[9] https://doi.org/10.1021/acs.est.0c07955

[10] IRENA report. Global hydrogen trade to meet the 1.5C climate goal: Part II - Technology review of hydrogen carriers

[11] https://www.belfercenter.org/sites/default/files/files/publication/Report_EU%20Hydrogen_FINAL.pdf

[12] https://www.nrel.gov/docs/fy14osti/61758.pdf

[13] https://ietresearch.onlinelibrary.wiley.com/doi/10.1049/iet-rpg.2020.0453(andref17therein)

[14] https://www.thyssenkrupp.com/en/newsroom/press-releases/pressdetailpage/thyssenkrupps-water-electrolysis-technology-qualified-as-primary-control-reserve--eon-and-thyssenkrupp-bring-hydrogen-production-to-the-electricity-market-83355

[15] https://pubs.rsc.org/en/content/articlelanding/2020/se/d0se00067a

[16] https://www.sciencedirect.com/science/article/abs/pii/S0306261920314549

[17] http://dx.doi.org/10.1016/j.ijhydene.2015.06.090

[18] doi:10.1016/j.ijggc.2011.09.008

[19] https://gasforclimate2050.eu/?smd_process_download=1&download_id=471

[20] http://www.co2europipe.eu/

[21] https://doi.org/10.1016/j.ijhydene.2018.06.121

[22] https://gasforclimate2050.eu/?smd_process_download=1&download_id=718

[23] https://doi.org/10.1016/j.rser.2018.09.027

[24] https://doi.org/10.1016/j.rser.2017.05.288

[25] https://doi.org/10.1109/JPHOTOV.2021.3136805

[26] https://www.irena.org/publications/2021/Jun/Renewable-Power-Costs-in-2020

[27] https://doi.org/10.1016/j.esr.2021.100636

[28] http://aures2project.eu/wp-content/uploads/2021/06/AURES_II_D5_2_financing_conditions.pdf

[29] https://doi.org/10.1016/j.eneco.2020.104783

3 Reviewer 2

Comment:
The article investigates synthetic chemical energy carriers, hydrogen, methane, methanol, ammonia and Fischer-Tropsch fuels, produced using electricity from Renewable Energy Source (RES) as fossil substitutes. They model the sourcing of feedstock chemicals, synthesis and transport along nine different Energy Supply Chains to Germany and compare import options for seven locations around the world against each other and with domestically sourced alternatives on the basis of their respective cost per unit of hydrogen and energy delivered. The article is rigorous and methodological choices are well motivated. I recommend publication after minor revisions.

Our response:
We thank the reviewer for their kind comments. We have made extensive revisions based on their suggestions, as well as recalculating some results and adding new graphics.

Comment:
It is great that you make the model openly available but it would also good if you could include some or the equations used in the model also in the main article so it would be easier to follow for the reader.

Our response:
We have added a chapter “Model equations” describing central equations to the supplementary materials and referenced it in the main text under “Materials and methods”.

Comment:
Please explain p.a., I assume it stands for per year.

Our response:
Indeed, p.a. Indicates “per annum” / “per year”. We have added this explanation to the text.

Comment:
Please write out WACC where you first introduce it, it is explained later in the text.

Our response:
The term WACC is introduced in the beginning of the “Materials and methods” section where it is also written out.

Comment:
You use 5

Our response:
The 5

Comment:
Line 427 You write “The lowest cost option for import is at 77EUR/MWhLHV by H2 pipeline from DK. Runner-up ESCs are also by H2 pipelines and imports by HVDC from DK at 92EUR/MWhLHV.” You mention H2 pipelines from DK in both pathways is it by mistake? I do not fully follow.

Our response:
This is indeed a mistake in our manuscript. We’ve corrected the sentence and updated it with the new results obtained after considering both reviewers comments.

Comment:
You do consider expected domestic use of the chemical feedstock. Would it for the remote location potentially also make a difference if considering the need from countries that are closer than Germany? How would this look at a global scale?

Our response:
Yes, we expect this would make a difference. The exact effects do heavily depend on the conditions and assumptions to be made about the future. One could expect a global hydrogen (energy commodity) market to form in which a shorter transport distance would constitute a competitive advantage, thus a potential redirection of commodity streams. We think this is a very interesting question and we are aware of some work already being conducted in that direction, including by our group. We consider our study a first step into this direction but recognise that an answer to this question is beyond the scope of our study. We've highlighted this in the limitations with the following paragraph added to the end: "In the broader picture we have considered imports to a single country, Germany, in our study. In reality there is to be expected a complex web of trade and competition between importing and exporting countries and individual actors. Our study is a first step into this direction."

Comment:
How you take the synthesis process flexibility into account seem good but it would be good if you could describe how it is incorporated in the model a bit more. "In addition to the economic perspective of operating synthesis processes at a high utilisation rate, some synthesis processes may be designed for continuous operation from a chemical process point of view [16, 19] and not be suited for flexible operation or standby. We take this into account be setting the must-run capacity of the methanation and ammonia synthesis processes to 30

Our response:
We have extended the paragraph and added the constraint equation to the supplementary materials. The new paragraph reads as follows:

> 'We consider this by assuming a must-run capacity for the methanation and ammonia synthesis processes of 30 % each, based on what could potentially be feasible for the methanation [36] and Haber-Bosch [26] processes. Methanol and FT fuel synthesis are assumed to run at a minimum of 94.25 % capacity corresponding to a maximum of 3 weeks downtime for e.g. maintenance per year. The must-run capacity is assumed for the aggregated availability factor of the whole respective process plant, e.g. a must-run capacity of 94.25 % translates to a maximum of 5.75 % of the facilities capacities being unavailable for maintenance or other reasons at the same time (see **??**).'

Comment:
From your figures, I can see that you are considering hydrogen storage. I would be good if you could elaborate a bit more on how you have determined the needed size of the

hydrogen storage for the different chemical feedstock. I have not managed to find this in the text, but apologies if it is there and I missed it.

Our response:
All storage capacities are determined endogenously within our model. We have added to following sentence to the subsection "Battery and chemical storage" to highlight this important aspect:

> 'The storage technology options available for each ESC are as shown in **??**. Storage capacities are endogenously determined by the model and represent optimal capacities under the given constraints minimising the objective function.'

Comment:
For the direct air capture you are referring to the wrong technology data sheet (`technology_data_for_industrial_process_heat_0002.xlsx`). Should be that connected to carbon capture and storage I think.

Our response:
Thanks for spotting this. When we originally wrote the manuscript the direct air capture technology data was indeed part of the mentioned datasheet. In the meantime it has been moved into a new technology data sheet dedicated to CO2 capture, transport and storage. We have updated the reference accordingly.

Comment:
It is not clear for what demand of end energy in German the cost is calculated. I assume the larger the demand the higher the demand due to the increased cost for electricity.

Our response:
Yes, your assumption is correct, cf. figure 3. To allow for comparison between all ESCs we require the same import volume from all ESCs, including the pseudo-imports (domestic production) from Germany to Germany. The imported amount is 120 TWh:

> 'We assume an energy import volume of 120 TWh based on the estimated hydrogen energy demand (LHV) for 2030 in the German Hydrogen Strategy [37].
> '

Comment:
The argument for why you consider DAC and not recirculating CO2 could be further

elaborating on and backed up with references.

Our response:
We have restructured the paragraph explaining why we only consider a CO2 cycle via the atmosphere and not direct recirculation of CO2 using CC at the point of emission. The new paragraph reads as follows:

> 'We do not consider the alternative approach of carbon capture (CC) at the location of use and back-transport of pure CO_2 to the location of energy carrier synthesis via a dedicated CO_2 infrastructure. This approach would require guaranteed capture of CO_2 from all use cases, which will prove complicated for applications where concentrated point sources are not available, like in aviation and individual mobility. Another obstacle to a perfect carbon cycle via CC is carbon leakage from imperfect CC which would need to be compensated through Direct Air Capture (DAC) infrastructure to ensure atmospheric carbon neutrality. Finally the infrastructure for recirculation of CO_2 would for most ESCs require additional dedicated infrastructure for CO_2 transport, incurring additional costs and complexity. While the costs of carbon capture at the location of use and back-transport may not be prohibitive, but such closed carbon cycles would require detailed analysis that is out of the scope of this paper. For the LOHC ESC recirculation is considered here as the infrastructure for recirculation of DBT in the LOHC ESC is the same as required for delivery of the hydrogen-loaded energy-carrying LOHC.'

Comment:
The cost difference between e-methanol and ammonia from you model is not seen in al studies for example not in Korberg et al (2021). It would be great if you could explain the reason for the significantly higher cost of methanol.

Our response:
The results we present are a direct consequence of the methods and assumptions we use. After the revisions the differences between LCoE and LCoH between methanol and ammonia are smaller compared to the results in the original manuscript.
We consider this comment as two separate questions and answer them with reference to the original manuscript. The changes we have made for the revisions are reflected in the tables and marked as 'revision'.
'*Why are the cost between Ammonia and Methanol noticeably different?*'
The Ammonia ESC only requires Hydrogen and Nitrogen as chemical feedstocks. The Methanol ESC on the other hand requires CO2 as feedstock, which is expensive and energy-intensive to source using direct air capture technology. This leads to a difference in cost between Ammonia and Methanol ESCs as can be seen from the cost composition comparison in Fig. 6 in our paper.

'Why is this cost difference not found in other studies, e.g. Korberg et al (2021)?'
We can not give a general answer to this question as most studies either vary significantly with regards to technology assumptions, methodology or both. Korberg et al (2021) use much more favourable cost and technology assumptions for sourcing of the CO2 feedstock and less favourable assumptions for sourcing Nitrogen for Ammonia production. We assume this leads to the difference in results between the studies. We can not give a definite answer as we do not have access to the full results of Korberg et al. We elaborate on the difference between our study and Korberg et al with a focus on CO2 in the following paragraphs:
We see the major differences in assumptions between their and our study (selection):

- Cost assumptions

		Korberg et al.	Our study[a] original[b]	Our study[a] revision[b]
Methanol synthesis	investment [MEUR/MW_{fuel}]	0.52	3	0.65
	FOM [%/p.a.]	4	1.76	3
	Lifetime [a]	25	20	20
	Efficiency [%]	79	71[c]	
Ammonia & Haber-Bosch synthesis	investment [MEUR/MW_{fuel}]	0.44	5.3	1.3
	FOM [%/p.a.]	2	4	3
	Lifetime [a]	20	20	30
	Efficiency [%]	87	71.7[c]	

[a] See tables 7 and 8. 2030 cost assumptions.
[b] 'original' are previous results before our revisions to which the reviewers comment and our answer relates, 'revisions' are the changed results after our revisions.
[c] Considering electricity and H_2 (LHV) input.

  - Higher investment cost increase the cost for producing the respective energy carrier
  - Higher (lower) lifetimes decrease (increase) the production cost of the respective energy carrier
  - Higher (lower) FOM increase (decrease) the production cost of the respective energy carrier
  - Higher (lower) efficiencies decrease (increase) the production cost of the respective energy carrier

- Discount rate
  - Assumed: 3% by Korberg et al. vs. 5% and 10% WACC in our study
  - A higher (lower) discount rate changes whether the lifetime of an installation has a higher (lower) effect relative to the FOM on the product cost (results of the Net Present Value investment calculation method).

- We assume Korberg et al (2021) are also using the NPV with their discount rate but were unable to find a which method they are using for their "annuity calculation"

- Methods
  - Korberg et al use an Excel model without optimisation or temporal resolution. They themselves note in their paper e.g. that "It is difficult to determine a specific storage size in the context of this analysis without using modelling tools, e.g. on plant or energy system level, but the authors assume using steel tank storage with a capacity that can supply the demand for 24 h together with a 30
  - We did encounter the same problem as well as an earlier version of this study also relied on an Excel model without optimisation and time resolution. With the results of this earlier unpublished model we decided that such a model is inadequate as too many assumptions have to be made on storage capacities and utilisation factors of all components and conversion steps in the model due to the variability of the renewable electricity sources (RES) used. This lead us to create the model we are using here which leverages synthetic time-series for RES at an hourly resolution and differentiated among quality classes with the therefrom derived supply curves.
  - This major methodological difference leads to Korberg et al having to make a lot of assumptions exogenously which are different to the results we obtain after optimization endogenously. These differences make it further hard to compare the results of Korberg et al with ours. This affects e.g.:

	Korberg et al	Our study[a]		
		original[b]	revised[b]	
Offshore wind CF	53 %	30.9 % to 48.1 %		
Electrolysis CF	53 %	57.4 %	57 %	for MeOH
		63.4	62.3 %	for NH_3
Electricity cost	33	45.63	43.9	for MeOH
[EUR/MWh]		38.37	37.4	for NH_3
H2 tank capacity[c]	24h +	62.7 h	61.7 h	for MeOH
	30% (buffer)	16.25 h	9.6 h	for NH_3

[a] Results based on `results.csv` which can be found on Zenodo, for 2030, 10% WACC, exporter DK, shipping-meoh and shipping-lnh3.
[b] 'original' are previous results before our revisions to which the reviewers comment and our answer relates, 'revisions' are the changed results after our revisions.
[c] Relative to maximum MeOH or NH3 synthesis capacity

- Cost for CO2 / direct air capture: We highlight this aspect specifically as we presume it to be the major driver behind the different results in Korberg et al (2021) vs. our results for the cost of Methanol and Ammonia.
  - Investment cost assumption: Korberg et al. note in Table 1 "Carbon Capture"

cost of 400 EUR/t_{CO_2}. We believe these are meant as 400 EUR/(t_{CO2} year) which is 41.6% lower than our assumption (684.93 EUR/(t_{CO2} year), cf. table 7 in our paper)

- Energy demand: Korberg et al mention in their supplementary materials ("A 3) Electrofuels") an electricity demand of 225 kW h/t_{CO2} and free-of-charge waste heat as input to their direct air capture process. For our study we assume 0.4 MWh_{el}/t_{CO2} as direct input and 0.533 MWh_{el} as indirect input for heat pumps to provide 1.6 $MWh_{thermal}$ of heat to the direct air capture plant.
- For our study the DAC plus heat pump investment is 20.6% of total of cost (51.6 EUR/MWh_{MeOH}). The electricity demand for DAC and heat pumps to provide enough CO_2 for the methanol synthesis is 0.933 MWh_{el}/MWh_{MeOH}, i.e. in terms of cost 42.6 EUR/MWh_{MeOH} for electricity for DAC and CO2.
- The cost of CO2 for 1 MWh_{MeOH} in our paper is thus ca. 94 EUR/MWh_{MeOH} ca. 30% of the total cost of methanol (250 EUR/MWh_{MeOH} for DK in 2030 at 10% p.a. WACC) compared to a reported total cost of 119 EUR/MWh for E-methanol in Fig. 3 of in the publication of Korberg et al (2021).
- For Ammonia production no CO2 is required leading to lower cost in Ammonia production in our study compared to Methanol cost. This can be seen in Fig 6. in the cost composition of the ESCs where e.g. NH3 (l) shipping from Argentina is cheaper than methanol shipping from Argentina by the cost for DAC and some PV plus wind cost (required for the additional electricity demand).
- Without having access to the disaggregated results of Korberg et al (2021) we can only assume their in comparison optimistic assumptions for direct air capture and difference in methodology to be the origin of the qualitative and quantitative differences in results between both studies.

Comment:
Consider changing the name of the y-axis on Figure 3 "Marginal electricity cost". For some it may be confusing with the term marginal as this is used for different things. I am not sure I fully understood how it is derived but is the word marginal needed.

Our response:
We have renamed the y-axis as "Levelised cost of electricity [EUR/MWh]", which also fits in this context, to avoid confusion

3.1 References by Reviewer 2

Korberg, A., Brynolf, S., Grahn, M., Skrov, I., 2021. Techno-economic assessment of advanced fuels and propulsion systems in future fossil-free ships. Renewable and Sustainable Energy Reviews 142.

References

[1] Ishimoto Y, Voldsund M, Nekså P, Roussanaly S, Berstad D, Gardarsdottir SO. Large-Scale Production and Transport of Hydrogen from Norway to Europe and Japan: Value Chain Analysis and Comparison of Liquid Hydrogen and Ammonia as Energy Carriers. International Journal of Hydrogen Energy. 2020;45(58):32865–32883. doi:10.1016/j.ijhydene.2020.09.017.

[2] Lanphen S. Hydrogen Import Terminal; 2019. Available from: https://repository.tudelft.nl/islandora/object/uuid%3Ad2429b05-1881-4e42-9bb3-ed604bc15255.

[3] Schorn F, Breuer JL, Samsun RC, Schnorbus T, Heuser B, Peters R, et al. Methanol as a Renewable Energy Carrier: An Assessment of Production and Transportation Costs for Selected Global Locations. Advances in Applied Energy. 2021;3:100050. doi:10.1016/j.adapen.2021.100050.

[4] Brändle G, Schönfisch M, Schulte S. Estimating Long-Term Global Supply Costs for Low-Carbon Hydrogen. Applied Energy. 2021;302:117481. doi:10.1016/j.apenergy.2021.117481.

[5] Johnston C, Ali Khan MH, Amal R, Daiyan R, MacGill I. Shipping the Sunshine: An Open-Source Model for Costing Renewable Hydrogen Transport from Australia. International Journal of Hydrogen Energy. 2022;47(47):20362–20377. doi:10.1016/j.ijhydene.2022.04.156.

[6] Pfennig M, Böttger D, Häckner B, Geiger D, Zink C, Bisevic A, et al.. Global GIS-based Potential Analysis and Cost Assessment of Power-to-X Fuels in 2050; 2022. Available from: http://arxiv.org/abs/2208.14887.

[7] Staiß F, Adolf J, Ausfelder F, Erdmann C, Hebling C, Jordan T, et al. Optionen für den Import grünen Wasserstoffs nach Deutschland bis zum Jahr 2030. acatech - Deutsche Akademie der Technikwissenschaften; 2022. Available from: https://energiesysteme-zukunft.de/publikationen/analyse/transportoptionen-wasserstoff-2030.

[8] IRENA. Global Hydrogen Trade to Meet the 1.5°C Climate Goal: Trade Outlook for 2050 and Way Forward; 2022. Available from: https://www.irena.org/publications/2022/Jul/Global-Hydrogen-Trade-Outlook.

[9] Viral faisalovers. Heat; 2018. Available from: https://thenounproject.com/icon/heat-2090492/.

[10] IRENA. Green Hydrogen Cost Reduction; 2020. Available from: https://www.irena.org/publications/2020/Dec/Green-hydrogen-cost-reduction.

[11] IRENA. Global Hydrogen Trade to Meet the 1.5°C Climate Goal: Technology Review of Hydrogen Carriers; 2022. Available from: https://www.irena.org/publications/2022/Apr/Global-hydrogen-trade-Part-II.

[12] MacFarlane DR, Cherepanov PV, Choi J, Suryanto BHR, Hodgetts RY, Bakker JM, et al. A Roadmap to the Ammonia Economy. Joule. 2020;4(6):1186–1205. doi:10.1016/j.joule.2020.04.004.

[13] ENGIE, Siemens, Ecuity Consulting, UK's Science & Technology Facilities Council. Ammonia to Green Hydrogen Project: Feasibility Study; 2020. Available from: https://assets.publishing.service.gov.uk/government/uploads/system/uploads/attachment_data/file/880826/HS420_-_Ecuity_-_Ammonia_to_Green_Hydrogen.pdf.

[14] Methanol Institute. Methanol Price and Supply/Demand; 2022. Available from: https://www.methanol.org/methanol-price-supply-demand/.

[15] Dolan G. Overview of Global Methanol Fuel Blending; 2019. Available from: https://www.methanol.org/wp-content/uploads/2019/01/Dolan-TT-Methanol-Fuel-Blending-Workshop-24-Jan-2018.pdf.

[16] IRENA, Methanol Institute. Innovation Outlook: Renewable Methanol; 2021. Available from: https://www.methanol.org/wp-content/uploads/2020/04/IRENA_Innovation_Renewable_Methanol_2021.pdf.

[17] Caloric Anlagenbau GmbH. Hydrogen by Methanol Reforming; 2020. Available from: https://www.caloric.com/en/product/hydrogen-generation/hydrogen-by-methanol-reforming/.

[18] Chen C, Yang A. Power-to-Methanol: The Role of Process Flexibility in the Integration of Variable Renewable Energy into Chemical Production. Energy Conversion and Management. 2021;228:113673. doi:10.1016/j.enconman.2020.113673.

[19] IEA. The Cost of Capital in Clean Energy Transitions – Analysis; 2021. Available from: https://www.iea.org/articles/the-cost-of-capital-in-clean-energy-transitions.

[20] Statistisches Bundesamt. 51000-0006: Aus- und Einfuhr (Außenhandel): Deutschland, Monate, Warensystematik; 2022. Available from: https://www-genesis.destatis.de/genesis/online?operation=table&code=51000-0006&bypass=true&levelindex=0&levelid=1667074103053#abreadcrumb.

[21] Investing com. ICE Dutch TTF Natural Gas Futures Historical Prices; 2022. Available from: https://www.investing.com/commodities/ice-dutch-ttf-gas-c1-futures-historical-data.

[22] Ewing R. ICIS: Global Ammonia Prices Jump as Key Plants Go Offline; 2021. Available from: https://www.icis.com/explore/resources/news/2021/02/12/10606213/global-ammonia-prices-jump-as-key-plants-go-offline.

[23] Vectors Point. Energy Consumption; 2019. Available from: https://thenounproject.com/search/?q=Consumption&i=2565142.

[24] Methanol Institute. Methanol Price and Supply/Demand; 2021. Available from: https://www.methanol.org/methanol-price-supply-demand/.

[25] eurostat. Natural Gas Price Statistics - Statistics Explained; 2020. Available from: https://ec.europa.eu/eurostat/statistics-explained/index.php?title=Natural_gas_price_statistics#Natural_gas_prices_for_non-household_consumers.

[26] Danish Energy Agency, Energinet. Technology Data for Renewable Fuels (04/2021); 2021. Available from: https://ens.dk/sites/ens.dk/files/Analyser/technology_data_for_renewable_fuels.pdf.

[27] Niermann M, Drünert S, Kaltschmitt M, Bonhoff K. Liquid Organic Hydrogen Carriers (LOHCs) – Techno-Economic Analysis of LOHCs in a Defined Process Chain. Energy & Environmental Science. 2019;12(1):290–307. doi:10.1039/C8EE02700E.

[28] Molnar G. Economics of Gas Transportation by Pipeline and LNG. In: Hafner M, Luciani G, editors. The Palgrave Handbook of International Energy Economics. Cham: Springer International Publishing; 2022. p. 23–57. Available from: https://doi.org/10.1007/978-3-030-86884-0_2.

[29] Danish Energy Agency. Technology Data for Carbon Capture, Transport and Storage; 2021.

[30] Lauri K, Jouko R, Nicklas N, Sebastian T. Scenarios and New Technologies for a North-European CO2 Transport Infrastructure in 2050. Energy Procedia. 2014;63:2738–2756. doi:10.1016/j.egypro.2014.11.297.

[31] Reuß M, Grube T, Robinius M, Preuster P, Wasserscheid P, Stolten D. Seasonal Storage and Alternative Carriers: A Flexible Hydrogen Supply Chain Model. Applied Energy. 2017;200:290–302. doi:10.1016/j.apenergy.2017.05.050.

[32] Danish Energy Agency, Energinet. Technology Data for Renewable Fuels (04/2022);

2022. Available from: https://ens.dk/sites/ens.dk/files/Analyser/technology_data_for_renewable_fuels.pdf.

[33] DNV GL. Study on the Import of Liquid Renewable Energy: Technology Cost Assessment; 2020.

[34] Danish Energy Agency. Technology Data for Energy Transport; 2021.

[35] Agora Verkehrswende, Agora Energiewende, Frontier Economics. The Future Cost of Electricity-Based Synthetic Fuels; 2018. Available from: https://www.agora-energiewende.de/en/publications/the-future-cost-of-electricity-based-synthetic-fuels-1/.

[36] Götz M, Lefebvre J, Mörs F, McDaniel Koch A, Graf F, Bajohr S, et al. Renewable Power-to-Gas: A Technological and Economic Review. Renewable Energy. 2016;85:1371–1390. doi:10.1016/j.renene.2015.07.066.

[37] Bundesministerium für Wirtschaft und Energie. Die Nationale Wasserstoffstrategie; 2020. Available from: https://www.bmwi.de/Redaktion/DE/Publikationen/Energie/die-nationale-wasserstoffstrategie.html.

Acronyms

AE alkaline electrolysis.

CC carbon capture.

DAC Direct Air Capture.

DBT dibenzyltoluene.

DE Germany.

ESC Energy Supply Chain.

FOM Fixed Operation & Maintenance.

FT fuel Fischer-Tropsch fuel.

LCoE Levelised Cost of Energy.

LCoH Levelised Cost of Hydrogen.

LHV Lower Heating Value.

LNG liquefied natural gas.

LOHC Liquid Organic Hydrogen Carrier.

MSR methanol steam reforming.

PEM proton exchange membrane.

RES Renewable Energy Source.

SMR steam methane reforming.

WACC Weighted Average Cost of Capital.
