# Supplementary material for: Correction: Import options for chemical energy carriers from renewable sources to Germany
Source: PLoS One. 2025 Jan 24;20(1):e0314578. doi: 10.1371/journal.pone.0314578 (PMC11760567; doi:10.1371/journal.pone.0314578)
Supplement: S3 Table — (PDF) [file pone.0314578.s001.pdf]

## S 9 Table Technology assumptions

All technology costs are given in EUR2015. Inflation adjustment was done where necessary assuming a 2 % p.a. inflation rate.

**Table 7.** Technology cost and lifetime assumptions used for 2030/2040/2050. A machine readable version of the input assumption can be found in the Zenodo and GitHub repositories listed in the data availability section.

| technology | parameter | year | value | unit | source |
|---|---|---|---|---|---|
| Ammonia cracker | CAPEX | 2030 | 1062107.74 | | |
| | | 2040 | 794849.98 | EUR/MW(H2) | Ishimoto et al. (2020): 10.1016/j.ijhydene.2020.09.017 , table 6. |
| | | 2050 | 527592.22 | | |
| | lifetime | 2030 | 25.0 | | |
| | | 2040 | 25.0 | years | Ishimoto et al. (2020): 10.1016/j.ijhydene.2020.09.017 , table 7. |
| | | 2050 | 25.0 | | |
| | FOM | 2030 | 4.3 | | |
| | | 2040 | 4.3 | %/year | Ishimoto et al. (2020): 10.1016/j.ijhydene.2020.09.017 , table 7. |
| | | 2050 | 4.3 | | |
| CCGT | CAPEX | 2030 | 830.00 | | |
| | | 2040 | 815.00 | EUR/kW | Danish Energy Agency, technology_data_for(el)_and_dh.xlsx |
| | | 2050 | 800.00 | | |
| | lifetime | 2030 | 25.0 | | |
| | | 2040 | 25.0 | years | Danish Energy Agency, technology_data_for(el)_and_dh.xlsx |
| | | 2050 | 25.0 | | |
| | FOM | 2030 | 3.35 | | |
| | | 2040 | 3.3 | %/year | Danish Energy Agency, technology_data_for(el)_and_dh.xlsx |
| | | 2050 | 3.25 | | |
| CH4 (g) fill compressor station | CAPEX | 2030 | 1498.95 | | |
| | | 2040 | 1498.95 | EUR/MW(CH4) | Guesstimate, based on H2 (g) pipeline and fill compressor station cost. |
| | | 2050 | 1498.95 | | |
| | lifetime | 2030 | 20.0 | | |
| | | 2040 | 20.0 | years | Assume same as for H2 (g) fill compressor station. |
| | | 2050 | 20.0 | | |

Table 7 (continued).

| technology | parameter | year | value | unit | source |
|---|---|---|---|---|---|
| CH4 (g) pipeline | FOM | 2030 | 1.7 | %/year | Assume same as for H2 (g) fill compressor station. |
| | | 2040 | 1.7 | | |
| | | 2050 | 1.7 | | |
| | CAPEX | 2030 | 79.00 | EUR/MW/km | Guesstimate. |
| | | 2040 | 79.00 | | |
| | | 2050 | 79.00 | | |
| | lifetime | 2030 | 50.0 | years | Assume same as for H2 (g) pipeline in 2050 (CH4 pipeline as mature technology). |
| | | 2040 | 50.0 | | |
| | | 2050 | 50.0 | | |
| CH4 (g) submarine pipeline | FOM | 2030 | 1.5 | %/year | Assume same as for H2 (g) pipeline in 2050 (CH4 pipeline as mature technology). |
| | | 2040 | 1.5 | | |
| | | 2050 | 1.5 | | |
| | CAPEX | 2030 | 114.89 | EUR/MW/km | Kaiser (2017): 10.1016/j.marpol.2017.05.003 . |
| | | 2040 | 114.89 | | |
| | | 2050 | 114.89 | | |
| | lifetime | 2030 | 30.0 | years | d'Amore-Domenech et al (2021): 10.1016/j.apenergy.2021.116625 , supplementary material. |
| | | 2040 | 30.0 | | |
| | | 2050 | 30.0 | | |
| CH4 evaporation | FOM | 2030 | 3.0 | %/year | d'Amore-Domenech et al (2021): 10.1016/j.apenergy.2021.116625 , supplementary material. |
| | | 2040 | 3.0 | | |
| | | 2050 | 3.0 | | |
| | CAPEX | 2030 | 87.60 | EUR/kW(CH4) | Calculated, based on Lochner and Bothe (2009): https://doi.org/10.1016/j.enpol.2008.12.012 and Fasihi et al 2017, table 1, https://www.mdpi.com/2071-1050/9/2/306 |
| | | 2040 | 87.60 | | |
| | | 2050 | 87.60 | | |
| | lifetime | 2030 | 30.0 | years | Fasihi et al 2017, table 1, https://www.mdpi.com/2071-1050/9/2/306 |
| | | 2040 | 30.0 | | |
| | | 2050 | 30.0 | | |
| | FOM | 2030 | 3.5 | %/year | Lochner and Bothe (2009): https://doi.org/10.1016/j.enpol.2008.12.012 and Fasihi et al 2017, table 1, https://www.mdpi.com/2071-1050/9/2/306 |

Table 7 (continued).

| technology | parameter | year | value | unit | source |
|---|---|---|---|---|---|
| | | 2040 | 3.5 | | |
| | | 2050 | 3.5 | | |
| CH4 liquefaction | CAPEX | 2030 | 232.13 | | |
| | | 2040 | 232.13 | EUR/kW(CH4) | Calculated, based on Lochner and Bothe (2009): https://doi.org/10.1016/j.enpol.2008.12.012 and Fasihi et al 2017, table 1, https://www.mdpi.com/2071-1050/9/2/306 |
| | | 2050 | 232.13 | | |
| | lifetime | 2030 | 25.0 | | |
| | | 2040 | 25.0 | years | Fasihi et al 2017, table 1, https://www.mdpi.com/2071-1050/9/2/306 |
| | | 2050 | 25.0 | | |
| | FOM | 2030 | 3.5 | | |
| | | 2040 | 3.5 | %/year | Fasihi et al 2017, table 1, https://www.mdpi.com/2071-1050/9/2/306 |
| | | 2050 | 3.5 | | |
| CO2 liquefaction | CAPEX | 2030 | 16.03 | | |
| | | 2040 | 16.03 | EUR/t_CO2/h | Mitsubish Heavy Industries Ltd. and IEA (2004): https://ieaghg.org/docs/General_Docs/Reports/PH4-30%20Ship%20Transport.pdf . |
| | | 2050 | 16.03 | | |
| | lifetime | 2030 | 25.0 | | |
| | | 2040 | 25.0 | years | Guesstimate, based on CH4 liquefaction. |
| | | 2050 | 25.0 | | |
| | FOM | 2030 | 5.0 | | |
| | | 2040 | 5.0 | %/year | Mitsubish Heavy Industries Ltd. and IEA (2004): https://ieaghg.org/docs/General_Docs/Reports/PH4-30%20Ship%20Transport.pdf . |
| | | 2050 | 5.0 | | |
| CO2 storage tank | CAPEX | 2030 | 2528.17 | | |
| | | 2040 | 2528.17 | EUR/t_CO2 | Lauri et al. 2014: doi: 10.1016/j.egypro.2014.11.297, Table 3. |
| | | 2050 | 2528.17 | | |
| | lifetime | 2030 | 25.0 | | |
| | | 2040 | 25.0 | years | Lauri et al. 2014: doi: 10.1016/j.egypro.2014.11.297, pg. 2746 . |
| | | 2050 | 25.0 | | |
| | FOM | 2030 | 1.0 | | |
| | | 2040 | 1.0 | %/year | Lauri et al. 2014: doi: 10.1016/j.egypro.2014.11.297, pg. 2746 . |

Table 7 (continued).

| technology | parameter | year | value | unit | source |
|---|---|---|---|---|---|
| | | 2050 | 1.0 | | |
| Fischer-Tropsch | CAPEX | 2030 | 650711.26 | EUR/MW_FT | Agora Energiewende (2018): The Future Cost of Electricity-Based Synthetic Fuels (https://www.agora-energiewende.de/en/publications/the-future-cost-of-electricity-based-synthetic-fuels-1/), table 8: “Reference scenario”. |
| | | 2040 | 565647.83 | | |
| | | 2050 | 480584.39 | | |
| | lifetime | 2030 | 20.0 | years | Danish Energy Agency, Technology Data for Renewable Fuels (04/2022), Data sheet “Methanol to Power”. |
| | | 2040 | 20.0 | | |
| | | 2050 | 20.0 | | |
| | FOM | 2030 | 3.0 | %/year | Agora Energiewende (2018): The Future Cost of Electricity-Based Synthetic Fuels (https://www.agora-energiewende.de/en/publications/the-future-cost-of-electricity-based-synthetic-fuels-1/), section 6.3.2.1. |
| | | 2040 | 3.0 | | |
| | | 2050 | 3.0 | | |
| General liquid hydrocarbon storage (product) | CAPEX | 2030 | 169.79 | EUR/m3 | Stelter and Nishida 2013: https://webstore.iea.org/insights-series-2013-focus-on-energy-security , pg. 8F . |
| | | 2040 | 169.79 | | |
| | | 2050 | 169.79 | | |
| | lifetime | 2030 | 30.0 | years | Stelter and Nishida 2013: https://webstore.iea.org/insights-series-2013-focus-on-energy-security , pg. 11. |
| | | 2040 | 30.0 | | |
| | | 2050 | 30.0 | | |
| | FOM | 2030 | 6.25 | %/year | Stelter and Nishida 2013: https://webstore.iea.org/insights-series-2013-focus-on-energy-security , figure 7 and pg. 12 . |
| | | 2040 | 6.25 | | |
| | | 2050 | 6.25 | | |
| H2 (g) fill compressor station | CAPEX | 2030 | 4478.00 | EUR/MW(H2) | Danish Energy Agency, Technology Data for Energy Transport (2021), pg. 164, Figure 14 (Fill compressor). |
| | | 2040 | 4478.00 | | |
| | | 2050 | 4478.00 | | |
| | lifetime | 2030 | 20.0 | years | Danish Energy Agency, Technology Data for Energy Transport (2021), pg. 168, Figure 24 (Fill compressor). |
| | | 2040 | 20.0 | | |
| | | 2050 | 20.0 | | |
| | FOM | 2030 | 1.7 | %/year | Guidehouse 2020: European Hydrogen Backbone report, https://guidehouse.com/-/media/www/site/downloads/energy/2020/gh_european-hydrogen-backbone_report.pdf (table 3, table 5) |
| | | 2040 | 1.7 | | |
| | | 2050 | 1.7 | | |

Table 7 (continued).

| technology | parameter | year | value | unit | source |
|---|---|---|---|---|---|
| H2 (g) pipeline | CAPEX | 2030 | 226.47 | | |
| | | 2040 | 226.47 | EUR/MW/km | European Hydrogen Backbone Report (June 2021): https://gasforclimate2050.eu/wp-content/uploads/2021/06/EHB_Analysing-the-future-demand-supply-and-transport-of-hydrogen_June-2021.pdf. |
| | | 2050 | 226.47 | | |
| | lifetime | 2030 | 50.0 | | |
| | | 2040 | 50.0 | years | Danish Energy Agency, Technology Data for Energy Transport (2021), Excel datasheet: H2 140. |
| | | 2050 | 50.0 | | |
| | FOM | 2030 | 3.17 | | |
| | | 2040 | 2.33 | %/year | Danish Energy Agency, Technology Data for Energy Transport (2021), Excel datasheet: H2 140. |
| | | 2050 | 1.5 | | |
| H2 (g) submarine pipeline | CAPEX | 2030 | 329.37 | | |
| | | 2040 | 329.37 | EUR/MW/km | Assume similar cost as for CH4 (g) submarine pipeline but with the same factor as between onland CH4 (g) pipeline and H2 (g) pipeline (2.86). This estimate is comparable to a 36in diameter pipeline calaculated based on d'Amore-Domenech et al (2021): 10.1016/j.apenergy.2021.116625 , supplementary material (=251 EUR/MW/km). |
| | | 2050 | 329.37 | | |
| | lifetime | 2030 | 30.0 | | |
| | | 2040 | 30.0 | years | Assume same as for CH4 (g) submarine pipeline. |
| | | 2050 | 30.0 | | |
| | FOM | 2030 | 3.0 | | |
| | | 2040 | 3.0 | %/year | Assume same as for CH4 (g) submarine pipeline. |
| | | 2050 | 3.0 | | |
| H2 (l) storage tank | CAPEX | 2030 | 750.08 | | |
| | | 2040 | 750.08 | EUR/MWh(H2) | Reuß et al 2017, https://doi.org/10.1016/j.apenergy.2017.05.050 , Table 6. |
| | | 2050 | 750.08 | | |
| | lifetime | 2030 | 20.0 | | |
| | | 2040 | 20.0 | years | Reuß et al 2017, https://doi.org/10.1016/j.apenergy.2017.05.050 , Table 6. |
| | | 2050 | 20.0 | | |
| | FOM | 2030 | 2.0 | | |
| | | 2040 | 2.0 | %/year | Reuß et al 2017, https://doi.org/10.1016/j.apenergy.2017.05.050 , Table 6. |
| | | 2050 | 2.0 | | |
| H2 evaporation | CAPEX | 2030 | 143.64 | EUR/kW(H2) | IRENA (2022): Global Hydrogen Trade to Meet the 1.5° Climate Goal: Technology Review of Hydrogen Carriers, https://www.irena.org/publications/2022/Apr/Global-hydrogen-trade-Part-II , pg. 62f. |

Table 7 (continued).

| technology | parameter | year | value | unit | source |
|---|---|---|---|---|---|
| | | 2040 | 100.11 | | |
| | | 2050 | 56.59 | | |
| | lifetime | 2030 | 20.0 | years | Guesstimate. |
| | | 2040 | 20.0 | | |
| | | 2050 | 20.0 | | |
| | FOM | 2030 | 2.5 | %/year | DNV GL (2020): Study on the Import of Liquid Renewable Energy: Technology Cost Assessment, https://www.gie.eu/wp-content/uploads/filr/2598/DNV-GL_Study-GLE-Technologies-and-costs-analysis-on-imports-of-liquid-renewable-energy.pdf . |
| | | 2040 | 2.5 | | |
| | | 2050 | 2.5 | | |
| H2 liquefaction | CAPEX | 2030 | 870.56 | EUR/kW(H2) | IRENA (2022): Global Hydrogen Trade to Meet the 1.5° Climate Goal: Technology Review of Hydrogen Carriers, https://www.irena.org/publications/2022/Apr/Global-hydrogen-trade-Part-II , pg. 62f. |
| | | 2040 | 696.45 | | |
| | | 2050 | 522.34 | | |
| | lifetime | 2030 | 20.0 | years | Reuß et al 2017, https://doi.org/10.1016/j.apenergy.2017.05.050 , Table 9. |
| | | 2040 | 20.0 | | |
| | | 2050 | 20.0 | | |
| | FOM | 2030 | 2.5 | %/year | DNV GL (2020): Study on the Import of Liquid Renewable Energy: Technology Cost Assessment, https://www.gie.eu/wp-content/uploads/filr/2598/DNV-GL_Study-GLE-Technologies-and-costs-analysis-on-imports-of-liquid-renewable-energy.pdf . |
| | | 2040 | 2.5 | | |
| | | 2050 | 2.5 | | |
| HVDC inverter pair | CAPEX | 2030 | 162364.82 | EUR/MW | Hagspiel et al. (2014): doi:10.1016/j.energy.2014.01.025 , table A.2 . |
| | | 2040 | 162364.82 | | |
| | | 2050 | 162364.82 | | |
| | lifetime | 2030 | 40.0 | years | Hagspiel et al. (2014): doi:10.1016/j.energy.2014.01.025 , table A.2 . |
| | | 2040 | 40.0 | | |
| | | 2050 | 40.0 | | |
| | FOM | 2030 | 2.0 | %/year | Hagspiel et al. (2014): doi:10.1016/j.energy.2014.01.025 , table A.2 . |
| | | 2040 | 2.0 | | |
| | | 2050 | 2.0 | | |
| HVDC overhead | CAPEX | 2030 | 432.97 | EUR/MW/km | Hagspiel et al. (2014): doi:10.1016/j.energy.2014.01.025 , table A.2 . |
| | | 2040 | 432.97 | | |

Table 7 (continued).

| technology | parameter | year | value | unit | source |
|---|---|---|---|---|---|
| | | 2050 | 432.97 | | |
| | lifetime | 2030 | 40.0 | | |
| | | 2040 | 40.0 | years | Hagspiel et al. (2014): doi:10.1016/j.energy.2014.01.025 , table A.2 . |
| | | 2050 | 40.0 | | |
| | FOM | 2030 | 2.0 | | |
| | | 2040 | 2.0 | %/year | Hagspiel et al. (2014): doi:10.1016/j.energy.2014.01.025 , table A.2 . |
| | | 2050 | 2.0 | | |
| HVDC submarine | CAPEX | 2030 | 471.16 | | |
| | | 2040 | 471.16 | EUR/MW/km | Purvins et al. (2018): https://doi.org/10.1016/j.jclepro.2018.03.095 . |
| | | 2050 | 471.16 | | |
| | lifetime | 2030 | 40.0 | | |
| | | 2040 | 40.0 | years | Purvins et al. (2018): https://doi.org/10.1016/j.jclepro.2018.03.095 . |
| | | 2050 | 40.0 | | |
| | FOM | 2030 | 0.35 | | |
| | | 2040 | 0.35 | %/year | Purvins et al. (2018): https://doi.org/10.1016/j.jclepro.2018.03.095 . |
| | | 2050 | 0.35 | | |
| Haber-Bosch | CAPEX | 2030 | 1297.43 | | |
| | | 2040 | 1061.17 | EUR/kW__NH3 | Danish Energy Agency, data__sheets__for__renewable__fuels.xlsx |
| | | 2050 | 813.55 | | |
| | lifetime | 2030 | 30.0 | | |
| | | 2040 | 30.0 | years | Danish Energy Agency, data__sheets__for__renewable__fuels.xlsx |
| | | 2050 | 30.0 | | |
| | FOM | 2030 | 3.0 | | |
| | | 2040 | 3.0 | %/year | Danish Energy Agency, data__sheets__for__renewable__fuels.xlsx |
| | | 2050 | 3.0 | | |
| LNG storage tank | CAPEX | 2030 | 611.59 | | |
| | | 2040 | 611.59 | EUR/m3 | Hurskainen 2019, https://cris.vtt.fi/en/publications/liquid-organic-hydrogen-carriers-lohc-concept-evaluation-and-tech pg. 46 (59). |
| | | 2050 | 611.59 | | |

Table 7 (continued).

| technology | parameter | year | value | unit | source |
|---|---|---|---|---|---|
| | lifetime | 2030 | 20.0 | | |
| | | 2040 | 20.0 | years | Guesstimate, based on H2 (l) storage tank with comparable requirements. |
| | | 2050 | 20.0 | | |
| | FOM | 2030 | 2.0 | | |
| | | 2040 | 2.0 | %/year | Guesstimate, based on H2 (l) storage tank with comparable requirements. |
| | | 2050 | 2.0 | | |
| LOHC dehydrogenation | CAPEX | 2030 | 50728.03 | | |
| | | 2040 | 50728.03 | EUR/MW(H2) | Reuß et al 2017, https://doi.org/10.1016/j.apenergy.2017.05.050 , Table 9. |
| | | 2050 | 50728.03 | | |
| | lifetime | 2030 | 20.0 | | |
| | | 2040 | 20.0 | years | Reuß et al 2017, https://doi.org/10.1016/j.apenergy.2017.05.050 , Table 9. |
| | | 2050 | 20.0 | | |
| | FOM | 2030 | 3.0 | | |
| | | 2040 | 3.0 | %/year | Reuß et al 2017, https://doi.org/10.1016/j.apenergy.2017.05.050 , Table 9. |
| | | 2050 | 3.0 | | |
| LOHC hydrogenation | CAPEX | 2030 | 51259.54 | | |
| | | 2040 | 51259.54 | EUR/MW(H2) | Reuß et al 2017, https://doi.org/10.1016/j.apenergy.2017.05.050 , Table 9. |
| | | 2050 | 51259.54 | | |
| | lifetime | 2030 | 20.0 | | |
| | | 2040 | 20.0 | years | Reuß et al 2017, https://doi.org/10.1016/j.apenergy.2017.05.050 , Table 9. |
| | | 2050 | 20.0 | | |
| | FOM | 2030 | 3.0 | | |
| | | 2040 | 3.0 | %/year | Reuß et al 2017, https://doi.org/10.1016/j.apenergy.2017.05.050 , Table 9. |
| | | 2050 | 3.0 | | |
| LOHC loaded DBT storage | CAPEX | 2030 | 149.27 | | |
| | | 2040 | 149.27 | EUR/t | Density via Wissenschaftliche Dienste des Deutschen Bundestages 2020, https://www.bundestag.de/resource/blob/816048/454e182d5956d45a664da9eb85486f76/WD-8-058-20-pdf-data.pdf , pg. 11. |
| | | 2050 | 149.27 | | |
| | lifetime | 2030 | 30.0 | years | nan |

Table 7 (continued).

| technology | parameter | year | value | unit | source |
|---|---|---|---|---|---|
| | | 2040 | 30.0 | | |
| | | 2050 | 30.0 | | |
| | FOM | 2030 | 6.25 | | |
| | | 2040 | 6.25 | %/year | nan |
| | | 2050 | 6.25 | | |
| LOHC unloaded DBT storage | CAPEX | 2030 | 132.26 | | |
| | | 2040 | 132.26 | EUR/t | Density via Wissenschaftliche Dienste des Deutschen Bundestages 2020, https://www.bundestag.de/resource/blob/816048/454e182d5956d45a664da9eb85486f76/WD-8-058-20-pdf-data.pdf , pg. 11. |
| | | 2050 | 132.26 | | |
| | lifetime | 2030 | 30.0 | | |
| | | 2040 | 30.0 | years | nan |
| | | 2050 | 30.0 | | |
| | FOM | 2030 | 6.25 | | |
| | | 2040 | 6.25 | %/year | nan |
| | | 2050 | 6.25 | | |
| Methanol steam reforming | CAPEX | 2030 | 16318.43 | | |
| | | 2040 | 16318.43 | EUR/MW(H2) | Niermann et al (2021): 10.1016/j.rser.2020.110171 , table 4. |
| | | 2050 | 16318.43 | | |
| | lifetime | 2030 | 20.0 | | |
| | | 2040 | 20.0 | years | Niermann et al (2021): 10.1016/j.rser.2020.110171 , table 4. |
| | | 2050 | 20.0 | | |
| | FOM | 2030 | 4.0 | | |
| | | 2040 | 4.0 | %/year | Niermann et al (2021): 10.1016/j.rser.2020.110171 , table 4. |
| | | 2050 | 4.0 | | |
| NH3 (l) storage tank incl. liquefaction | CAPEX | 2030 | 161.93 | | |
| | | 2040 | 161.93 | EUR/MWh_NH3 | Calculated based on Morgan E. 2013: doi:10.7275/11KT-3F59 , Fig. 55, Fig 58. |
| | | 2050 | 161.93 | | |
| | lifetime | 2030 | 20.0 | | |
| | | 2040 | 20.0 | years | Morgan E. 2013: doi:10.7275/11KT-3F59 , pg. 290 |

Table 7 (continued).

| technology | parameter | year | value | unit | source |
|---|---|---|---|---|---|
| | | 2050 | 20.0 | | |
| | FOM | 2030 | 2.0 | | |
| | | 2040 | 2.0 | %/year | Guesstimate, based on H2 (l) storage tank. |
| | | 2050 | 2.0 | | |
| Steam methane reforming | CAPEX | 2030 | 470085.47 | | |
| | | 2040 | 470085.47 | EUR/MW(H2) | International Energy Agency (2015): Technology Roadmap Hydrogen and Fuel Cells , table 15. |
| | | 2050 | 470085.47 | | |
| | lifetime | 2030 | 30.0 | | |
| | | 2040 | 30.0 | years | International Energy Agency (2015): Technology Roadmap Hydrogen and Fuel Cells , table 15. |
| | | 2050 | 30.0 | | |
| | FOM | 2030 | 3.0 | | |
| | | 2040 | 3.0 | %/year | International Energy Agency (2015): Technology Roadmap Hydrogen and Fuel Cells , table 15. |
| | | 2050 | 3.0 | | |
| air separation unit | CAPEX | 2030 | 729306.18 | | |
| | | 2040 | 596501.02 | EUR/t(N2)/h | Danish Energy Agency, data_sheets_for_renewable_fuels.xlsx |
| | | 2050 | 457307.78 | | |
| | lifetime | 2030 | 30.0 | | |
| | | 2040 | 30.0 | years | Danish Energy Agency, data_sheets_for_renewable_fuels.xlsx |
| | | 2050 | 30.0 | | |
| | FOM | 2030 | 3.0 | | |
| | | 2040 | 3.0 | %/year | Danish Energy Agency, data_sheets_for_renewable_fuels.xlsx |
| | | 2050 | 3.0 | | |
| battery inverter | CAPEX | 2030 | 160.00 | | |
| | | 2040 | 100.00 | EUR/kW | Danish Energy Agency, technology_data_catalogue_for_energy_storage.xlsx |
| | | 2050 | 60.00 | | |
| | lifetime | 2030 | 10.0 | | |
| | | 2040 | 10.0 | years | Danish Energy Agency, technology_data_catalogue_for_energy_storage.xlsx, Note K. |
| | | 2050 | 10.0 | | |

Table 7 (continued).

| technology | parameter | year | value | unit | source |
|---|---|---|---|---|---|
| battery storage | FOM | 2030 | 0.34 | %/year | Danish Energy Agency, technology_data_catalogue_for_energy_storage.xlsx |
| | | 2040 | 0.54 | | |
| | | 2050 | 0.9 | | |
| | CAPEX | 2030 | 142.00 | EUR/kWh | Danish Energy Agency, technology_data_catalogue_for_energy_storage.xlsx |
| | | 2040 | 94.00 | | |
| | | 2050 | 75.00 | | |
| | lifetime | 2030 | 25.0 | years | Danish Energy Agency, technology_data_catalogue_for_energy_storage.xlsx |
| | | 2040 | 30.0 | | |
| | | 2050 | 30.0 | | |
| clean water tank storage | FOM | - | - | | |
| | CAPEX | 2030 | 67.63 | EUR/m3-H2O | Caldera et al 2016: Local cost of seawater RO desalination based on solar PV and windenergy: A global estimate. (https://doi.org/10.1016/j.desal.2016.02.004), Table 1. |
| | | 2040 | 67.63 | | |
| | | 2050 | 67.63 | | |
| | lifetime | 2030 | 30.0 | years | Caldera et al 2016: Local cost of seawater RO desalination based on solar PV and windenergy: A global estimate. (https://doi.org/10.1016/j.desal.2016.02.004), Table 1. |
| | | 2040 | 30.0 | | |
| | | 2050 | 30.0 | | |
| | FOM | 2030 | 2.0 | %/year | Caldera et al 2016: Local cost of seawater RO desalination based on solar PV and windenergy: A global estimate. (https://doi.org/10.1016/j.desal.2016.02.004), Table 1. |
| | | 2040 | 2.0 | | |
| | | 2050 | 2.0 | | |
| direct air capture | CAPEX | 2030 | 6000000.00 | EUR/(tCO2/h) | Danish Energy Agency, technology_data_for_carbon_capture_transport_storage.xlsx |
| | | 2040 | 5000000.00 | | |
| | | 2050 | 4000000.00 | | |
| | lifetime | 2030 | 20.0 | years | Danish Energy Agency, technology_data_for_carbon_capture_transport_storage.xlsx |
| | | 2040 | 20.0 | | |
| | | 2050 | 20.0 | | |
| | FOM | 2030 | 4.95 | %/year | Danish Energy Agency, technology_data_for_carbon_capture_transport_storage.xlsx |
| | | 2040 | 4.95 | | |
| | | 2050 | 4.95 | | |

Table 7 (continued).

| technology | parameter | year | value | unit | source |
|---|---|---|---|---|---|
| electrolysis | CAPEX | 2030 | 450.00 | EUR/kW_e | Danish Energy Agency, data_sheets_for_renewable_fuels.xlsx |
| | | 2040 | 300.00 | | |
| | | 2050 | 250.00 | | |
| | lifetime | 2030 | 30.0 | years | Danish Energy Agency, data_sheets_for_renewable_fuels.xlsx |
| | | 2040 | 32.0 | | |
| | | 2050 | 35.0 | | |
| | FOM | 2030 | 2.0 | %/year | Danish Energy Agency, data_sheets_for_renewable_fuels.xlsx |
| | | 2040 | 2.0 | | |
| | | 2050 | 2.0 | | |
| hydrogen storage tank incl. compressor | CAPEX | 2030 | 44.91 | EUR/kWh | Danish Energy Agency, technology_data_catalogue_for_energy_storage.xlsx |
| | | 2040 | 27.05 | | |
| | | 2050 | 21.00 | | |
| | lifetime | 2030 | 30.0 | years | Danish Energy Agency, technology_data_catalogue_for_energy_storage.xlsx |
| | | 2040 | 30.0 | | |
| | | 2050 | 30.0 | | |
| | FOM | 2030 | 1.11 | %/year | Danish Energy Agency, technology_data_catalogue_for_energy_storage.xlsx |
| | | 2040 | 1.85 | | |
| | | 2050 | 1.9 | | |
| industrial heat pump medium temperature | CAPEX | 2030 | 778.80 | EUR/kW | Danish Energy Agency, technology_data_for_industrial_process_heat.xlsx |
| | | 2040 | 730.00 | | |
| | | 2050 | 700.00 | | |
| | lifetime | 2030 | 20.0 | years | Danish Energy Agency, technology_data_for_industrial_process_heat.xlsx |
| | | 2040 | 20.0 | | |
| | | 2050 | 20.0 | | |
| | FOM | 2030 | 0.11 | %/year | Danish Energy Agency, technology_data_for_industrial_process_heat.xlsx |
| | | 2040 | 0.11 | | |
| | | 2050 | 0.1 | | |
| methanation | CAPEX | 2030 | 628.60 | EUR/kW(CH4) | Agora Energiewende (2018): The Future Cost of Electricity-Based Synthetic Fuels (https://www.agora-energiewende.de/en/publications/the-future-cost-of-electricity-based-synthetic-fuels-1/), table 6: “Reference scenario”. |

Table 7 (continued).

| technology | parameter | year | value | unit | source |
|---|---|---|---|---|---|
| | | 2040 | 554.59 | | |
| | | 2050 | 480.58 | | |
| | lifetime | 2030 | 20.0 | years | Guesstimate. |
| | | 2040 | 20.0 | | |
| | | 2050 | 20.0 | | |
| | FOM | 2030 | 3.0 | %/year | Agora Energiewende (2018): The Future Cost of Electricity-Based Synthetic Fuels (https://www.agora-energiewende.de/en/publications/the-future-cost-of-electricity-based-synthetic-fuels-1/), section 6.2.3.1 |
| | | 2040 | 3.0 | | |
| | | 2050 | 3.0 | | |
| methane storage tank incl. compressor | CAPEX | 2030 | 8629.20 | EUR/m3 | Storage costs per l: https://www.compositesworld.com/articles/pressure-vessels-for-alternative-fuels-2014-2023 (2021-02-10). |
| | | 2040 | 8629.20 | | |
| | | 2050 | 8629.20 | | |
| | lifetime | 2030 | 30.0 | years | Guesstimate, based on hydrogen storage tank by DEA. |
| | | 2040 | 30.0 | | |
| | | 2050 | 30.0 | | |
| | FOM | 2030 | 1.9 | %/year | Guesstimate, based on hydrogen storage tank by DEA. |
| | | 2040 | 1.9 | | |
| | | 2050 | 1.9 | | |
| methanolisation | CAPEX | 2030 | 650711.26 | EUR/MW_MeOH | Agora Energiewende (2018): The Future Cost of Electricity-Based Synthetic Fuels (https://www.agora-energiewende.de/en/publications/the-future-cost-of-electricity-based-synthetic-fuels-1/), table 8: "Reference scenario". |
| | | 2040 | 565647.83 | | |
| | | 2050 | 480584.39 | | |
| | lifetime | 2030 | 20.0 | years | Danish Energy Agency, Technology Data for Renewable Fuels (04/2022), Data sheet "Methanol to Power". |
| | | 2040 | 20.0 | | |
| | | 2050 | 20.0 | | |
| | FOM | 2030 | 3.0 | %/year | Agora Energiewende (2018): The Future Cost of Electricity-Based Synthetic Fuels (https://www.agora-energiewende.de/en/publications/the-future-cost-of-electricity-based-synthetic-fuels-1/), section 6.3.2.1. |
| | | 2040 | 3.0 | | |
| | | 2050 | 3.0 | | |
| seawater desalination | CAPEX | 2030 | 32882.05 | EUR/(m3-H2O/h) | Caldera et al 2017: Learning Curve for Seawater Reverse Osmosis Desalination Plants: Capital Cost Trend of the Past, Present, and Future (https://doi.org/10.1002/2017WR021402), Table 4. |
| | | 2040 | 26297.44 | | |

Table 7 (continued).

| technology | parameter | year | value | unit | source |
|---|---|---|---|---|---|
| | | 2050 | 21025.64 | | |
| | lifetime | 2030 | 30.0 | years | Caldera et al 2016: Local cost of seawater RO desalination based on solar PV and windenergy: A global estimate. (https://doi.org/10.1016/j.desal.2016.02.004), Table 1. |
| | | 2040 | 30.0 | | |
| | | 2050 | 30.0 | | |
| | FOM | 2030 | 4.0 | %/year | Caldera et al 2016: Local cost of seawater RO desalination based on solar PV and windenergy: A global estimate. (https://doi.org/10.1016/j.desal.2016.02.004), Table 1. |
| | | 2040 | 4.0 | | |
| | | 2050 | 4.0 | | |
